# Dynamics of clonal hematopoiesis and cellular responses to stress-induced toxicity in autologous stem cell transplantation

**DOI:** 10.1038/s41375-025-02823-z

**Published:** 2025-12-19

**Authors:** Catarina M. Stein, Raphael Hablesreiter, Friederike Christen, Pelle Löwe, Coral Fustero-Torre, Klara Kopp, Benjamin N. Locher, Lena Nitsch, Robert Altwasser, Johanna Franziska Kerschbaum, Lars Bullinger, Leif S. Ludwig, Paulina M. Strzelecka, Frederik Damm

**Affiliations:** 1 Charité – Universitätsmedizin Berlin, Corporate Member of Freie Universität Berlin and Humboldt-Universität zu Berlin, and Berlin Institute of Health, Department of Hematology, Oncology, and Cancer Immunology, Berlin, Germany; 2 Berlin School of Integrative Oncology (BSIO), Berlin, Germany; 3 Berlin Institute of Health at Charité—Universitätsmedizin Berlin, Berlin, Germany; 4 Max-Delbrück-Center for Molecular Medicine in the Helmholtz Association, Berlin Institute for Medical Systems Biology, Berlin, Germany; 5 Department of Biology, Chemistry, Pharmacy, Freie Universität Berlin, Berlin, Germany; 6 German Cancer Consortium (DKTK) and German Cancer Research Center (DKFZ), Heidelberg, Germany

**Keywords:** Cancer genetics, Stem-cell research, Myeloma

## Abstract

Autologous stem cell transplantation (ASCT) involves harvesting hematopoietic stem and progenitor cells (HSPCs) prior to chemotherapy and subsequent repopulation of the bone marrow. This process imposes a bottleneck, providing a framework to dissect the unresolved short- and long-term clonal dynamics during hematopoietic reconstitution. By integrating bulk error-corrected targeted sequencing of clonal hematopoiesis (CH)-associated genes with mitochondrial single-cell Assay for Transposase-Accessible Chromatin sequencing (mtscATAC-seq), we characterized mutational trajectories in frequently altered hematological genes and traced clonal evolution through somatic mitochondrial DNA variants, revealing post-transplant cellular heterogeneity and clonal architecture. Among 60 patients (multiple myeloma, *n* = 51; non-Hodgkin lymphoma, *n* = 6; Hodgkin lymphoma, *n* = 3), CH-associated mutations were identified in 53% pre-ASCT, predominantly involving *DNMT3A*. A transient increase in mutation counts and gene diversity occurred 10-25 days post-ASCT, with a gradual clonal expansion two years post-transplantation. Tandem ASCT amplified clonal complexity, with a twofold increase in mutation count and gene-level diversity, while preserving clonal trajectories across both transplant courses. Mitochondrial single-cell profiling in longitudinal samples of 3 patients showed patient-specific immune reconstitution and clonal dynamics, with balanced multilineage output from graft HSPCs. Collectively, our findings provide a firsthand comprehensive view of ASCT-induced clonal dynamics and immune reconstitution, paving the way for targeted gene-specific post-transplant monitoring.

## Introduction

Autologous stem cell transplantation (ASCT) is a key component in the first-line treatment of multiple myeloma (MM) and often used for relapsed/refractory Hodgkin and non-Hodgkin lymphomas, providing sustained remissions and prolonged progression-free survival [1]. Hematopoietic stem and progenitor cells (HSPCs) are harvested from the patient, and following high-dose chemotherapy (HDC), repopulated in the bone marrow (BM). This process initiates a potent yet constrained bottleneck, as only a limited pool of HSPC clones appear to drive hematopoietic regeneration [2].

However, hematopoiesis can be altered by the presence of somatically mutated hematopoietic clones, an age-related condition referred to as clonal hematopoiesis (CH) [3]. These mutations frequently occur in genes responsible for epigenetic regulation (*e.g., DNMT3A, TET2, ASXL1*) and DNA damage response (*e.g.*,* PPM1D, TP53*), and have been shown to confer a proliferation advantage for mutated HSPCs over their wild-type counterparts [4–6]. Consequently, CH has been associated with increased risks for therapy-related secondary malignancies and overall mortality in lymphoma and MM patients undergoing ASCT [7–9]. These findings, along with growing evidence that CH clones, even low-frequency ones, persist or expand during HDC and post-transplantation, highlight their potential to influence hematopoietic recovery and thus clinical outcomes [10–12]. Particularly *TP53* and *PPM1D* mutations, often associated with prior chemotherapy exposure, provide a selective advantage in the regenerating hematopoietic environment leading to impaired recovery and increased risk of therapy-related myeloid neoplasms [2, 13–15]. Yet, the impact of cytotoxic stress within the regenerative environment following ASCT on short- and long-term dynamics of CH clones remains insufficiently understood, underscoring the need to elucidate how selective pressures influence the persistence and expansion of genetically distinct HSPC populations.

While nuclear somatic mutations define CH, their low variant allele frequencies (VAFs) and sparse detectability in peripheral blood mononuclear cells (PBMCs) limit resolution of clonal hierarchies at single-cell level [16, 17]. Mitochondrial DNA (mtDNA) mutations, which accumulate with age, are associated with hematological malignancies and often co-occur with CH driver mutations, have therefore been proposed as markers for clonal expansion and CH [18, 19]. Leveraging this, mitochondrial single-cell Assay for Transposase-Accessible Chromatin with sequencing (mtscATAC-seq) integrates chromatin accessibility profiling with mtDNA genotyping to simultaneously infer clonal lineages and differentiation trajectories within the hematopoietic system [20, 21]. In contrast to somatic nuclear mutations, the mitochondrial genome’s compact size ( ~ 16.6 kb), high mutation rate and elevated copy number make mtDNA a robust genetic marker for lineage tracing and monitoring regenerative processes such as ASCT [22–24]. High levels of heteroplasmy, the proportion of mitochondrial genomes in a cell containing a specific mutation, further facilitate single-cell variant detection [21, 25, 26].

mtDNA-based lineage tracing has proven effective in physiological and malignant hematopoiesis, tracking HSPC output and resolving clonal hierarchies in MM and leukemias. These mutations remain stable in homeostasis but change markedly under selective pressures such as chemotherapy or allogeneic transplantation, uncovering dynamic changes in clonal composition and chromatin states [27–29]. However, how ASCT shapes regeneration remains unclear, and a comprehensive understanding of clonal trajectories under hematopoietic stress post-ASCT is crucial for anticipating complications, optimizing transplant strategies, and improving patient outcomes.

Building on these insights, we employed a multi-modal genomic approach, combining high-depth, error-corrected bulk targeted sequencing of 45 CH-associated genes with mtscATAC-seq to longitudinally profile nuclear and mitochondrial mutation dynamics and immune reconstitution in patients undergoing ASCT. This approach allowed us to: (i) assess prevalence and gene-specific characteristics of CH prior to ASCT, (ii) track clonal competition and fitness during hematopoietic recovery, and (iii) resolve cellular identities and lineage biases of mtDNA-defined clones at single-cell resolution. By integrating temporal, genetic, and epigenetic layers, our study provides a novel comprehensive view of clonal behavior in response to ASCT-induced cytotoxic stress, distinguishing early stress responses from long-term hematopoietic remodeling.

## Methods

### Patient cohort and study design

60 patients with MM (*n* = 51), relapsed/refractory non-Hodgkin lymphoma (*n* = 6), and Hodgkin lymphoma (*n* = 3) undergoing primary ASCT were recruited at Charité – Universitätsmedizin Berlin from February 2021 until November 2024.

Peripheral blood (PB) was collected preTx (median of 3 days (d) prior to transplantation) and at defined timepoints post-ASCT: Tx1_1 (10-25 d post-transplantation; *n* = 59), Tx1_2 (30-85 d; *n* = 52), and Tx1_3 (90-195 d; *n* = 47). In addition, the autologous graft product was collected on transplantation day (graft1; *n* = 58). Long-term follow-up samples were collected ~1 year (Tx1_4; *n* = 2) and ~2 years (Tx1_5; *n* = 5) post-transplantation, when available. 10 patients underwent tandem ASCT (Tx2), with graft and PB samples collected at analogous follow-up intervals: graft2 (*n* = 10), Tx2_1 (*n* = 10), Tx2_2 (*n* = 7), and Tx2_3 (*n* = 6) (Supplementary Fig. S1).

From a total of 304 collected samples, viable PBMCs were isolated by Ficoll density centrifugation and cryopreserved in liquid nitrogen. Graft cells were washed with phosphate-buffered saline, centrifuged at 400× *g* for 8 min at 4 °C, and cryopreserved.

Written informed consent was obtained in accordance with the Declaration of Helsinki and was approved by the Charité – Universitätsmedizin Berlin ethics review committee (EA1/286/20). Eligible patients were ≥ 18 years, of any sex, with no prior ASCT or active infectious disease (Supplementary Table S1).

### Error-corrected targeted sequencing

Genomic DNA was extracted using the QIAamp DNA Mini Kit (Qiagen, Hilden, Germany) according to the manufacturer’s protocol and subjected to a previously described error-corrected targeted sequencing workflow [30]. Libraries were generated using a library preparation kit with a custom-designed targeted sequencing panel (Twist BioScience, San Francisco, CA, USA) covering 45 genes frequently mutated in CH (Supplementary Table S2). Unique molecular identifiers (xGen UDI-UMI adapters, Integrated DNA Technologies, Coralville, IA, USA) allowed for bioinformatic error-correction. Libraries were sequenced on NovaSeq6000/X platforms (Illumina, San Diego, CA, USA) in paired-end mode (Supplementary Fig. S2). Somatic mutations with a VAF ≥ 0.5% were identified using our in-house Snakemake [31] pipeline and variants were filtered, as previously described [32–34] (Supplementary Fig. S3+4, Supplementary Table S3, Supplementary Methods). Patients with at least one mutation ≥ 0.5% VAF were defined as CH-positive (CH^+^).

### Mitochondrial single-cell ATAC library generation and sequencing

Samples were sorted to isolate viable (SYTOX^TM^ Blue-negative) and CD66b-negative cells (Supplementary Fig. S5). Adapted from the 10x Genomics Chromium single-cell Assay for Transposase-Accessible Chromatin (ATAC) solution user guide (CG000209, 10x Genomics, Pleasanton, CA, USA) and in accordance with Lareau et al. [20], libraries were generated using the Chromium Next GEM single-cell ATAC Kit v2 (1000406, 10x Genomics, Pleasanton, CA, USA). To enable simultaneous intranuclear and mitochondrial access of the Tn5 transposase, additional fixation and modified lysis steps were incorporated [20, 35] (Supplementary Table S4). All libraries were sequenced on Illumina NovaSeq6000/X platforms (Illumina, San Diego, CA, USA) in paired-end mode (Supplementary Fig. S6). Sequencing reads were processed using 10x Genomics CellRanger-ATAC (v2.1) [36] and the mitochondrial genome analysis toolkit (v0.6.1) [35] following Lareau et al. [20] (Supplementary Methods). Downstream analyses were conducted using Signac [37] on cells with ≥ 10× mtDNA depth, ≥ 2000 ATAC fragments, ≥ 35% of fragments mapped to accessibility peaks, > 2 transcription start sites, and a nucleosome score < 4 (Supplementary Fig. S7). mtDNA variant calling was performed jointly across timepoints, with high-confidence somatic heteroplasmic variants defined by a strand correlation > 0.65 and a variance-to-mean ratio > 0.01.

### Statistical analysis

All analyses were performed in R (R Foundation for Statistical Computing, Vienna, Austria, v4.2.3). Due to the exploratory nature of the study, no prior sample size calculation was conducted. Data was tested for normal distribution using the Shapiro-Wilk test. Pairwise comparisons were performed using Wilcoxon rank-sum tests or Fisher’s exact tests, indicated in figure legends. A 95% confidence interval was chosen and a two-sided *p* value < 0.05 was considered statistically significant, without multiple testing adjustment. For single-gene analyses, patients with any mutation in a given gene were classified as mutated, regardless of VAF or co-mutations. For patients with multiple mutations, the largest VAF was used to determine clone size.

## Results

### Detection of CH in patients receiving ASCT

**Fig. 1:**
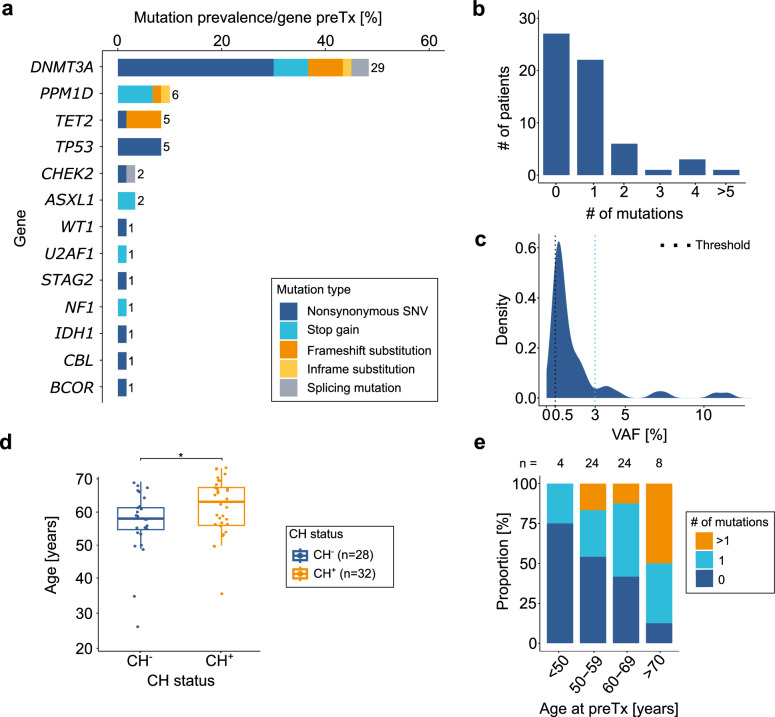
Comprehensive somatic mutation analysis of clonal hematopoiesis (CH)-associated genes of 60 patients prior to autologous stem cell transplantation. **a** Stacked bar plot showing the gene-specific prevalence of somatic mutations, colored by mutation type. The number of mutations per gene is indicated at the top of each bar. **b** Bar plot showing the number of patients carrying single or multiple mutations. **c** Density plot of the variant allele frequency (VAF) distribution across all mutations. The black dashed line indicates the VAF cutoff of 0.5%, the blue dashed line indicates a VAF of 3%. **d** Boxplot of patients age stratified by CH status. The number of patients per condition is given in brackets. *p < 0.05, Wilcoxon rank-sum test. **e** Stacked bar plot showing the number of mutations per patient across age bins, with patient counts indicated above bars. SNV Single-nucleotide variant.

To assess CH prevalence, we performed bulk error-corrected targeted sequencing of PB DNA from 60 patients preTx. 56 CH mutations in 32/60 patients, corresponding to a 53% CH prevalence (Supplementary Table S3), were identified. The most frequently mutated genes were *DNMT3A* (*n* = 29 in 18 patients), *PPM1D* (*n* = 6 in 5 patients), *TET2* (*n* = 5 in 5 patients) and *TP53* (*n* = 5 in 2 patients) (Fig. 1a). Among CH^+^ individuals, 70% carried mutations in *DNMT3A, TET2, ASXL1* genes, and 24% had mutations in DNA damage response genes. Most alterations were nonsynonymous single-nucleotide variants (*n* = 30, 54%), particularly within *DNMT3A* and *TP53*, while truncating mutations were predominantly in *PPM1D* (*n* = 4, 67%) and *ASXL1* (*n* = 2) (Fig. 1a**)**. 66% of patients (21/32) harbored a single mutation, while 34% (11/32) carried multiple mutations, with up to seven mutations in one individual (Fig. 1b). The overall median VAF (VAF_med_) was 0.94% (range: 0.5-11.5%) with most mutations (48/56, 86%) below 3%. Among these, *TET2* mutations showed the highest VAF_med_ (2.1%) (Fig. 1c). *DNMT3A*/*DNMT3A* was the most common mutation pair (*n* = 9 co-mutations) (Supplementary Fig. S8).

CH^+^ patients were significantly older than CH-negative (CH^-^) patients preTx (median age: 63 vs. 58 years, *p* = 0.03) (Fig. 1d, Supplementary Table S1). Additionally, mutation counts per patient increased with age (Fig. 1e). CH^+^ patients exhibited significantly lower serum C-reactive protein levels preTx vs. CH^-^ patients (3 mg/l vs. 1.15 mg/l, *p* = 0.004) but no further differences were observed in transplantation-related clinical characteristics, including harvested CD34^+^ cell levels, hospitalization duration, leukocyte/thrombocyte recovery, or post-transplantation complications (Supplementary Fig. S9, Supplementary Tables S1+S5).

### CH dynamics following ASCT

**Fig. 2:**
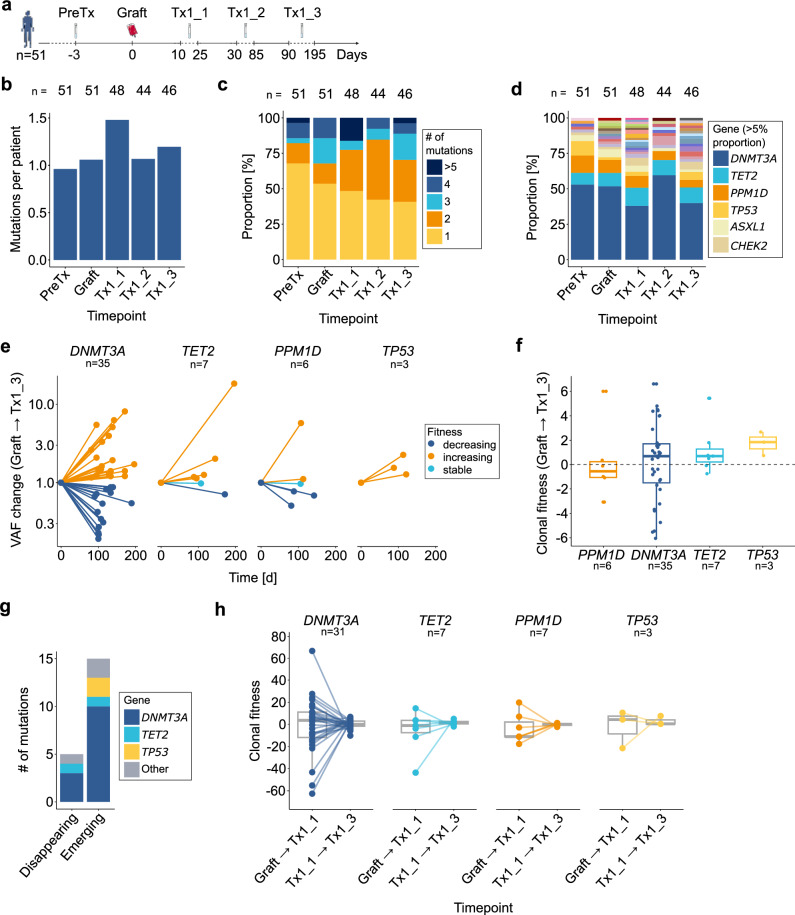
Longitudinal dynamics of clonal hematopoietic (CH) mutations in 51 patients following autologous stem cell transplantation. **a** Schematic overview of collected samples relative to the graft infusion (d0). Tx1_1 = 10-25 days (d), Tx1_2 = 30–85 d, Tx1_3 = 90–195 d post-transplantation. **b** Bar plot of normalized mutation counts per patient across timepoints. PreTx was used as reference. **c** Stacked bar plot displaying the number of mutations per patient for each timepoint. Patient counts are indicated above the bars. **d** Stacked bar plot showing the distribution of mutated genes by timepoint. Genes with a proportion > 5% are highlighted and color-coded in the legend. Patient counts are indicated above the bars. **e** Relative variant allele frequency (VAF) changes from the most prevalent genes (*DNMT3A*, *TET2*, *PPM1D*, and *TP53*) over time, from graft to Tx1_3. Colors represent clonal fitness (*s*) categories: increasing (*s* > 0.25/year), decreasing (*s* < -0.25/year), and stable (-0.25 ≥ *s* ≤ 0.25/year). Mutation counts are indicated above the plot. **f** Boxplot of clonal fitness for the most prevalent mutated genes with mutation counts indicated. **g** Bar plot showing the emergence and disappearance of mutations with VAF ≥ 0.5% from graft to Tx1_3, with colors indicating the mutated genes. **h** Clonal fitness estimates comparison between graft/Tx1_1 and Tx1_1/Tx1_3 for the most frequently mutated genes. Mutation counts are indicated.

To investigate the effects of cytotoxic stress associated with ASCT on CH, we longitudinally profiled CH-associated mutations in graft samples and at defined post-transplantation timepoints (Tx1_1, Tx1_2, and Tx1_3). Longitudinal sequencing data were available for 51 patients, with a median follow-up of 113 d (range: 75-198 d) (Fig. 2a, Supplementary Fig. S10).

A temporary shift in CH status was observed at Tx1_1, characterized by an increased proportion of CH^+^ patients (Supplementary Fig. S11). Although overall CH prevalence increased only slightly, clonal complexity peaked at this timepoint with the median number of mutations per patient increasing from 1 to 1.5, with individuals harboring up to nine mutations (Fig. 2b+c). Concomitantly, gene-level clonal diversity exhibited a mean fold increase of 1.6 × relative to preTx/graft samples (range: 0.5×-7×, Fig. 2d). Over time, mutation counts and VAFs progressively returned to preTx/graft levels by Tx1_3 (0.93% vs. 1.10% at preTx and Tx1_3, respectively).

Clinical consequences of CH during engraftment showed no significant differences in PB counts or leukopenia between CH^+^ and CH^-^ patients, though patients without leukopenia presented slightly higher clonal fitness (1.13 vs. 0.63) and more strongly expanding clones at Tx1_3 (Supplementary Fig. S12+S13).

We next evaluated gene-specific VAF trajectories of recurrent CH drivers, *DNMT3A* (*n* = 35), *TET2* (*n* = 7), *PPM1D* (*n* = 6), and *TP53* (*n* = 3), classifying clones as increasing, decreasing, or stable (Fig. 2e, Supplementary Fig. S14). While *TP53*-mutated clones exhibited consistent expansion from graft to Tx1_3, mutations in *DNMT3A*, *TET2*, and *PPM1D* exhibited balanced dynamics. To estimate clonal fitness, we modeled longitudinal VAF changes using a sigmoid growth model [33] (Supplementary Methods). Fitness estimates clustered around zero, suggesting no strong gene-specific selective advantage across the cohort. *PPM1D* mutations showed slightly negative fitness, whereas *DNMT3A*, *TET2*, and *TP53* exhibited modestly positive trends (Fig. 2f). Remarkably, 15 previously undetectable mutations (VAF < 0.5%) emerged during follow-up, including 10 *DNMT3A* and 2 *TP53* variants, while 5 clones regressed below detection threshold, further underscoring the dynamic nature of clonal competition in early reconstitution (Fig. 2g). Temporal resolution of clonal fitness revealed transient clonal instability with evident fluctuations between graft and Tx1_1, followed by a stabilization toward Tx1_3 consistent across all genes (Fig. 2h, Supplementary Fig. S15).

### Long-term mutational dynamics and the impact of tandem ASCT

To explore long-term dynamics of CH, we analyzed two clinically relevant post-ASCT trajectories: patients undergoing tandem ASCT and those receiving maintenance therapy with extended follow-up.

**Fig. 3:**
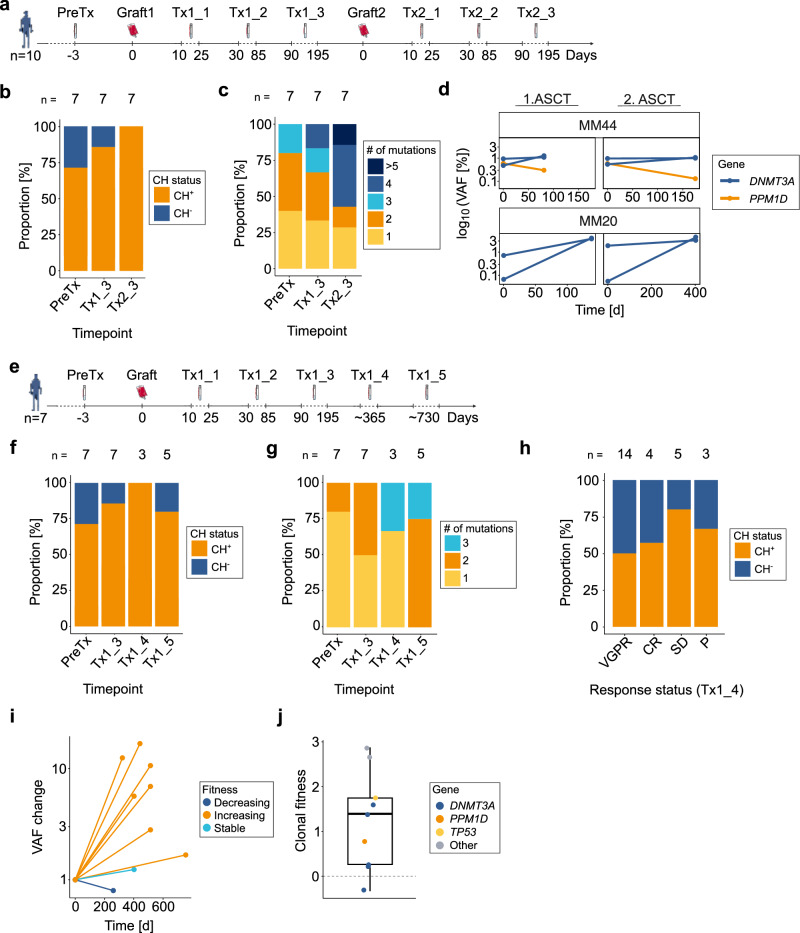
Long-term mutational dynamics and the impact of tandem autologous stem cell transplantation (ASCT). **a** Schematic overview of collected samples relative to the first graft infusion (d0), including 1^st^ ASCT (Tx1) and 2^nd^ ASCT (Tx2). Tx1/2_1 = 10-25 days (d), Tx1/2_2 = 30-85 d, Tx1/2_3 = 90-195 d post-transplantation. **b** Bar plot showing the proportion of clonal hematopoiesis positive (CH^+^) vs. negative (CH^-^) patients at preTx, Tx1_3, and Tx2_3, with patient counts indicated above the bars. **c** Stacked bar plot displaying the proportion of mutation counts per patient at preTx, Tx1_3 and Tx2_3. Patient counts are indicated above the bars. **d** Longitudinal clonal dynamics of exemplary patients MM44 and MM20 who underwent 1^st^ and 2^nd^ ASCT showing variant allele frequency (VAF) changes of *DNMT3A* and *PPM1D* mutations. **e** Schematic overview of the collected samples relative to the 1^st^ graft infusion (d0), including Tx1_4 ( ~ 1 year post-transplantation) and Tx1_5 ( ~ 2 years post-transplantation). **f** Bar plot showing the proportion of CH^+^ vs. CH^-^ patients at preTx, Tx1_3, Tx1_4, and Tx1_5, with number of patients at each timepoint indicated above the bars. **g** Stacked bar plot displaying the distribution of mutation counts per patient at preTx, Tx1_3, Tx1_4, and Tx1_5. Patient counts are indicated above the bars. **h** Stacked bar plot showing the proportion of CH status across treatment responses Tx1_4. **i** Relative VAF changes over time, from graft to Tx1_5. Colors represent clonal fitness (*s*) categories: increasing (*s* > 0.25/year), decreasing (*s* < -0.25/year), and stable (- 0.25 ≥ *s* ≤ 0.25/year). **j** Boxplot of clonal fitness from graft to Tx1_5, colored by mutated gene. CR Complete remission, PD Progressive disease, SD Stable disease, VGPR Very good partial remission.

We first investigated the impact of tandem ASCT by evaluating paired grafts (graft1/graft2) and serial PB samples collected after each transplant (Tx1_1-Tx1_3 and Tx2_1-Tx2_3) in ten MM patients, seven of whom had longitudinal sampling (median follow-up: 209 d; range:148-276 d) (Fig. 3a, Supplementary Fig. S1+S10). Among these, CH prevalence increased progressively from 71% preTx to 86% at Tx1_3 and reaching 100% by Tx2_3 (Fig. 3b). This trend was accompanied by an increase in clonal complexity, with the median number of CH mutations per patient doubling (from 2 to 4 mutations/patient) between Tx1_3 and Tx2_3, and mean gene-level clonal diversity rising 1.8-fold and twofold, respectively (Fig. 3c, Supplementary Fig. S16). Mutational trajectories were remarkably stable across both transplant courses: 81% (13/16) of mutations showed similar VAF patterns between 1^st^ and 2^nd^ ASCT, suggesting consistent clonal behavior under repeated cytotoxic pressure (Fig. 3d, Supplementary Fig. S16). A minority (19%, 3/16) of mutations demonstrated discordant behavior between tandem transplants.

Clinically, considering limited sample size, there were no significant differences in engraftment-related parameters between the two transplants (Supplementary Table S6).

To investigate whether post-ASCT dynamics reflected pre-existing clonal composition, we compared paired grafts. 88% (22/25) of mutations identified in graft1 were found in graft2 when applying a lowered VAF threshold of 0.1%, capturing three additional clones. Overall VAF_med_ were comparable between paired graft samples (0.7% vs. 0.6%, Supplementary Fig. S17).

In a complementary approach, we followed a second sub-cohort of seven patients (MM = 6; Hodgkin lymphoma = 1) up to 2 years post-ASCT (median follow-up: 514 d; range:340-755 d), with PB samples collected at Tx1_4 and Tx1_5 (Fig. 3e). Among these, two received lenalidomide maintenance therapy, exhibiting modest *DNMT3A* expansions (0.63/0.68% at graft2 to 1.5/1.3% at Tx2_3) and marked *ASXL1* growth (1.0% at graft2 to 16.8% at Tx2_3). Two CH^+^ patients lost their CH mutations over time, an *ATM* (0.73% at Tx1_3) and a *DNMT3A* mutation (0.72% at Tx1_3) (Fig. 3f). However, in the remaining CH^+^ individuals, the median number of mutations per patient continued to rise (1 vs. 1 vs. 2 at preTx, Tx1_4, and Tx1_5, respectively), accompanied by sustained gene-level diversity increases (1.6-fold relative to preTx) (Fig. 3g, Supplementary Fig. S18). At Tx1_4, CH^+^ patients exhibited higher rates of stable disease and progression in treatment response (Fig. 3h). Finally, long-term VAF trajectories revealed an upward trend in most clones (Fig. 3i+ j, Supplementary Fig. S18), suggesting that while CH clones may not exhibit aggressive expansion, they persist and gradually expand over time.

### Single-cell chromatin profiling reveals patient-specific cell type dynamics and gene activity following ASCT

**Fig. 4:**
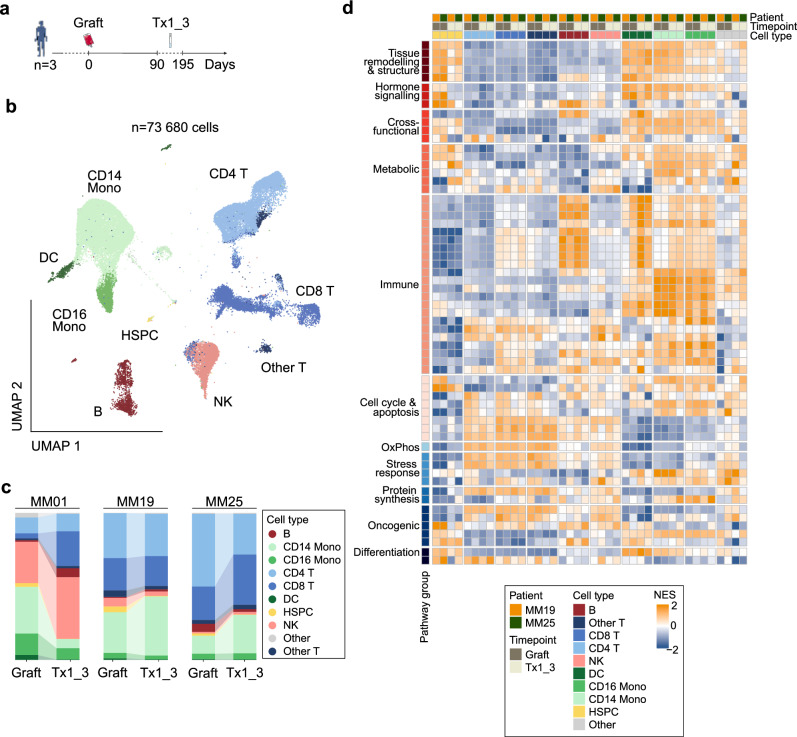
Single-cell chromatin profiling reveals cell type dynamics and gene activity following autologous stem cell transplantation. **a** Schematic overview of collected samples for patients with distinct clonal hematopoiesis (CH) mutational profiles (MM01: CH^-^ female; MM19: CH^+^ male with stable *DNMT3A* mutation; MM25: CH^+^ female with expanding *ASXL1* mutation). Peripheral blood mononuclear cells from the graft product and from peripheral blood at Tx1_3. **b** 2D Uniform manifold approximation and projection (UMAP) of in total 73 680 cells showing annotated cell types based on chromatin accessibility profiles from six integrated samples. **c** Stacked bar plot showing changes in cell type proportions between graft and Tx1_3 for each patient. **d** Heatmap from single sample gene set enrichment analysis showing normalized enrichment scores (NES) for pathways significantly altered (*p* < 0.01) in CH^+^ patients, MM19, and MM25. B B-cell, CD4 T CD4^+^ T-cell, CD8 T CD8^+^ T-cell, CD14 Mono CD14^+^ monocyte, CD16 Mono CD16^+^ monocyte, DC Dendritic cell, HSPC Hematopoietic stem and progenitor cell, NK Natural killer cell, Other T Other T-cell.

To extend the insights from bulk CH analysis to single-cell resolution, we used mtscATAC-seq to investigate chromatin accessibility in graft and Tx1_3 samples from three patients with distinct CH mutational profiles (MM01: CH^-^ female; MM19: CH^+^ male with stable *DNMT3A* mutation; MM25: CH^+^ female with expanding *ASXL1* mutation) and characterize cellular and regulatory heterogeneity during hematopoietic recovery (Fig. 4a, Supplementary Fig. S19, Supplementary Table S7). Across the three paired sample timepoints, we profiled 73 680 high-quality PBMCs (mean: 12 280 cells/sample; range: 7 149-17 652) with a mean per-cell read depth of 8 052× (range: 6 089×-11 603×) (Supplementary Fig. S7, Supplementary Tables S8+S10). Reference-based bridge integration enabled consistent annotation across all samples and revealed well-defined clusters corresponding to canonical immune populations (Fig. 4b, Supplementary Fig. S20+S21).

Longitudinal tracking of immune composition from graft to Tx1_3 revealed patient-specific immunological trajectories of hematopoietic recovery (Fig. 4c). Despite all individuals demonstrating multilineage recovery, the relative abundance of immune subsets exhibited substantial inter-patient heterogeneity. MM01 exhibited a pronounced presence of natural killer (NK) cells in the graft, with a post-transplant expansion from 27% to 42%, alongside a marked increase in CD8⁺ T-cells (3.6% vs. 23.8%) and a sharp decline in monocytes (48% vs. 14%). In contrast, MM19 maintained a balanced T-cell compartment (52% vs. 47%) with a moderate monocyte increase (31% vs. 42%). MM25 showed a monocyte expansion (16% vs. 31%) and CD4^+^ T-cell reduction (49% vs. 28%). As anticipated, HSPCs were enriched in graft products across all patients (*e.g.*, MM01: 2.5%, 435/17 152 cells) in comparison to Tx1_3 (*e.g*., MM01: 0.1%, 9/7 149 cells) (Fig. 4c, Supplementary Table S7).

To assess changes in gene regulatory activity, we performed pseudo-bulk differential peak accessibility analysis between graft and Tx1_3 samples stratified by cell types. We computed gene-level z-scores comparing graft and Tx1_3 to assess transcriptional consistency across patients (Supplementary Fig. S22, Supplementary Table S10). MM01 clustered distinctly from MM19 and MM25, exhibiting a unique gene activity signature, particularly in monocytes and NK cells, prompting separate downstream analyses to account for its divergent regulatory trajectory. To identify biologically relevant pathway changes, we conducted single-sample gene set enrichment analysis on chromatin accessibility profiles. Significant pathways between timepoints stratified by cell types across MM19 and MM25 were visualized in Fig. 4d, with MM01-specific dynamics depicted in Supplementary Fig. S23 with significant gene sets listed in Supplementary Table S11. Notably, clustering of pathway enrichment scores demonstrated that cell type were more pronounced than timepoint differences. Particularly in monocytes, MM19 and MM25 showed significant upregulation of cell cycle and apoptosis pathways post-ASCT, which was not observed in MM01.

### Single-cell profiling of mitochondrial mutation dynamics following ASCT

mtscATAC-seq simultaneously captures mtDNA variants in single-cells enabling clonal inference based on heteroplasmy, an independent and complementary clonal marker, and allowing us to investigate its clonal architectures and lineage relationships in hematopoietic cells post-ASCT [20, 35].

**Fig. 5:**
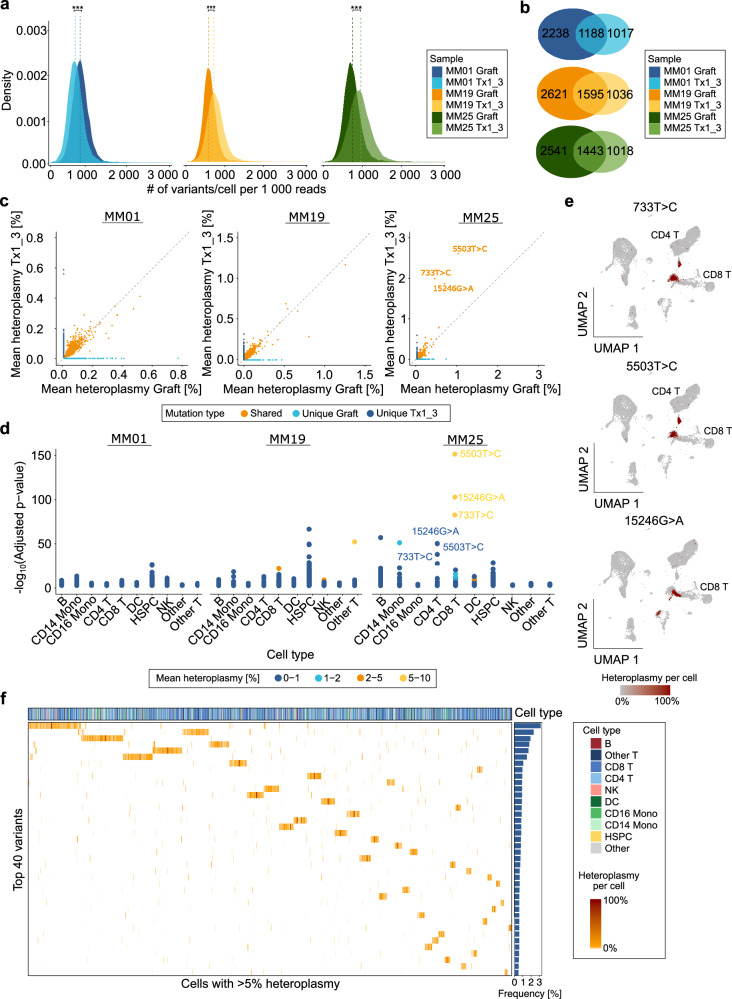
Single-cell profiling of mitochondrial mutation dynamics following autologous stem cell transplantation. **a** Density plot showing the distribution of mitochondrial mutations per cell at graft and Tx1_3 normalized to mitochondrial DNA (mtDNA) depth across all patients. ***p < 0.001, Wilcoxon rank-sum test. **b** Venn diagram depicting the overlap and sample-specific uniqueness of mtDNA mutations among the three patients from graft and Tx1_3. **c** Scatter plot comparing mean heteroplasmy of shared and unique mtDNA mutations between graft and Tx1_3 across all patients. The diagonal dashed line indicates perfect correlation. Note: Axes limits differ for patients to enhance visualization. **d** Stacked point plot showing adjusted *p *values from Kruskal-Wallis test of cell type-specific enrichment of mtDNA mutations across all patients. Mutations are colored by heteroplasmy bins. Only statistically significant (*p* < 0.05) mutations are displayed. **e** 2D Uniform manifold approximation and projection (UMAP) highlighting three mitochondrial mutations (733 T > C, 5 503 T > C and 15 246 G > A) expanding in patient MM25 in CD8^+^ and CD4^+^ T-cells. **f** Heatmap with hierarchical clustering of single cells showing the 40 most frequent heteroplasmic mtDNA mutations with a mean heteroplasmy > 5% in MM25 at Tx1_3. Only post-transplant cells harboring mtDNA mutations originating from graft hematopoietic stem and progenitor cells (HSPCs) are shown. Percentage of affected cells is displayed as a bar plot on the right. B B-cell, CD4 T CD4^+^ T-cell, CD8 T CD8^+^ T-cell, CD14 Mono CD14^+^ monocyte, CD16 Mono CD16^+^ monocyte, DC Dendritic cell, NK Natural killer cell, Other T Other T-cell.

Coverage across the mitochondrial genome was robust, with a mean depth of 38× (range: 22-50×), ensuring comprehensive variant detection (Supplementary Fig. S24). Across all samples, an average of 3 154 somatic mtDNA mutations/sample (range: 2 205-4 216) was observed (Supplementary Fig. S25). The substitution landscape revealed a characteristic enrichment of C > T and T > C transitions, a profile well-documented in mtDNA and reflective of replication-associated processes [38] (Supplementary Fig. S26). When stratifying mtDNA mutations/cell by patient and timepoint, normalized to mtDNA depth, the number of mutations per 1 000 reads consistently differed between samples, with MM01 showing a decrease and MM19/MM25 showing increases post-transplant (*p* < 0.001 for all; Fig. 5a). Correspondingly, mean heteroplasmy increased in Tx1_3 relative to the graft in MM19/MM25 but remained largely stable in MM01 (Supplementary Fig. S27).

To explore clonal persistence and evolution, we assessed the proportion of shared mtDNA variants between the two timepoints per patient. We found that 21% (1 188/5 631) of mtDNA mutations in MM01, 23% (1 595/6 847) in MM19, and 22% (1 443/6 445) in MM25 were retained post-transplantation (Fig. 5b).

Heteroplasmy levels revealed stable mutations in MM01 and MM19 from graft to Tx1_3, while MM25 showed a heteroplasmy increase for three variants: 733 T > C, 5 503 T > C, and 15 246 G > A (Fig. 5c). These mutations exhibited strong cell type specificity, with enrichment in CD8^+^ and CD4^+^ T-cells and heteroplasmy levels ranging from 5-10%, distinguishing them as unique features of MM25 (Fig. 5d). Uniform manifold approximation and projection further confirmed that these variants were restricted to the T-cell compartment (Fig. 5e). Specifically, 5 503 T > C and 733 T > C variants marked effector memory and central memory T-cells, respectively, showing high *SELL*, *IL7R* and *CCR7* gene activity. 5 503 > C positive cells additionally exhibited cytotoxic markers (*e.g*.*, GZMB, PRF1*). In contrast, 15 246 G > A defined an early exhausted T-cell subset by increased *TIGIT* and *TOX* gene activity (Supplementary Fig. S28).

We next investigated the trajectory of hematopoietic reconstitution by tracing the fate of graft-derived HSPC clones. Using variants identified in graft HSPCs, we filtered the Tx1_3 dataset to track cells originating from these progenitors and identified additional mutations acquired in these clones to infer their differentiation trajectory. The distribution of these derived mutations appeared largely uniform across cell types, with no dominant lineage bias (Fig. 5f, Supplementary Fig. S29+S30). Interestingly, in MM25, six distinct variants contributed to a substantial proportion of the reconstituted cell population, while in MM01 and MM19, the clonal architecture was simpler, with three and one dominant clone, respectively (Supplementary Fig. S29). In MM01, the expansion trajectory favored NK cells, in MM19 and MM25 T-cells, aligning with independent cell annotation data (Supplementary Fig. S30).

## Discussion

Our study presents a comprehensive, multi-modal characterization of clonal dynamics in response to cytotoxic stress following ASCT, revealing the dynamic nature of post-transplant reconstitution, shaped by the pre-existing clonal architecture of HSPCs.

CH was identified in 53% of patients undergoing ASCT, a prevalence surpassing reports in healthy and malignancy-based population studies [11, 14, 39], reflecting the synergistic mutational spectrum of age-related (*e.g., DNMT3A, TET2*) and therapy-associated genes (*e.g., PPM1D, TP53*) [13, 40]. Our cohort exhibited consistently low VAFs (VAF_med _= 0.94%), reflecting low-frequency CH clones that are not previously described using similar sequencing approaches but detectable with sensitive digital droplet polymerase chain reaction assays, which might explain the high overall prevalence at least partially [9, 11, 12]. Our technical approach using error-corrected sequencing enabled the detection of low-frequency clones (VAF ≥ 0.5%), uncovering a complex CH landscape with elevated prevalence [41].

Longitudinal analysis of CH clones showed that ASCT induced a transient, previously unreported, reshaping of the hematopoietic clonal architecture, marked by a short-lived clonal expansion driven by hematopoietic stress and temporary BM niche remodeling during engraftment, without evidence of long-term clonal dominance [42, 43]. Three months post-ASCT, *DNMT3A*, *TET2*, and *PPM1D* mutations showed no fitness advantage, while *TP53*-mutant clones expanded, reflecting their established role in therapy-related clonal evolution and resistance to DNA damage [7, 11]. The observed stability of *PPM1D* mutations aligns with previous findings, emphasizing that their survival advantage and clonal expansion emerge under DNA-damaging therapies like cisplatin, but not during BM transplant recovery [33, 44]. These findings highlight the importance of mutation-treatment interactions in CH and support a gene-specific risk model, where post-ASCT monitoring focuses on high-risk clones such as *TP53*, while most other clones remain stable and do not influence transplant decisions [8, 45].

Long-term follow-up, on the other hand, exhibited an expansion of pre-existing HSPC clones, and an increased clonal complexity, consistent with the gradual clonal evolution observed in aging and previous ASCT-exposed populations [3, 11]. Notably, CH^+^ individuals exhibited a trend toward increased disease progression at Tx1_4, reinforcing evidence that mutations, especially in *TP53* and *PPM1D*, are linked to adverse outcomes [7, 13, 14]. Additionally, our findings revealed that mutational dynamics are shaped by therapeutic exposure and pre-existing clonal architecture within graft HSPCs. Clones present in HSPCs in tandem transplants show highly similar trajectories post-transplantations, demonstrating that ASCT exerts a selective pressure favoring the expansion of existing CH clones rather than the emergence of new ones [7, 11, 13]. The strong mutational concordance between paired grafts supports the hypothesis that ASCT acts as a clonal bottleneck, enriching for fitter HSPCs capable of withstanding repeated cytotoxic stress [41].

While nuclear mutations have been central to lineage tracing, mtDNA mutations have emerged as novel powerful barcodes for clonal inference in hematopoietic contexts [21, 27, 35]. We effectively captured clonal mtDNA dynamics in patients exhibiting distinct CH trajectories, demonstrating increased heteroplasmy and mutational burden post-ASCT in CH^+^ patients, aligning with evidence that elevated heteroplasmy is associated with clonal expansion of CH clones and, when combined with CH status, enhances prediction of myeloid malignancy risk [18]. By tracking hematopoietic output through mtDNA mutations post-ASCT, we show patient-specific immune subset expansion, such as NK cells in MM01 or T-cells in MM25, despite a broad multilineage reconstitution. In MM01, NK cell expansion could reflect autoimmune priming due to pre-existing Hashimoto’s thyroiditis [46], while the T-cell bias aligns with prior observations in allogeneic settings [47], though its exact immunologic trigger remains elusive. These patterns support the concept of “clonal memory” where intrinsic HSPC programming and extrinsic factors, like inflammation, or prior therapy, shape early immune recovery and can predetermine lineage fate [48].

Chromatin accessibility profiling provided additional insight into early lineage regulation post-ASCT. In MM01 (CH^-^), monocytes showed downregulation of cell cycle and apoptosis pathways, while in MM19/MM25 (CH^+^), these programs were upregulated and accompanied by a monocyte expansion. In MM19, this pattern may reflect *DNMT3A*-driven self-renewal and myeloid expansion, whereas the T-cell bias in MM25 coincided with outgrowth of an *ASXL1*-mutant clone, in line with its role in disrupting epigenetic control of myeloid and lymphoid differentiation [49, 50]. While based on single cases, these findings suggest lineage-specific effects of CH mutations on immune recovery post-ASCT that warrant further investigation.

While our study provides valuable high-resolution insights, the limited sample size, particularly for long-term follow-up, limits broader applicability of our findings and hinders deeper comprehension between clonal evolution and clinical outcomes. In addition, mtDNA-based lineage tracing does not capture the complete epigenomic and genomic spectrum that governs cellular behavior post-ASCT, constraining our ability to fully assess functional dynamics of expanding clones. Future studies integrating multi-omics approaches, combining CH and mitochondrial data at single-cell resolution, as shown by Velten et al. in distinguishing healthy, and leukemic clones, will be essential to offer complementary insights and validate the functional and clinical implications of clonal hierarchies across post-transplant hematopoiesis [16, 17, 51].

Collectively, our integrated profiling of mtDNA and CH trajectories post-ASCT reveals a dynamic, and individualized hematopoietic regeneration shaped by HSPC architecture. Translating these clonal insights into clinical practice will be essential for advancing personalized care and improving long-term transplant outcomes.

## Supplementary information

Supplemental Material

Supplemental TableS3

Supplemental TableS9

Supplemental TableS10

## Data Availability

Raw sequencing data generated during the current study have been deposited in the European Nucleotide Archive (ENA) at EMBL-EBI under accession number PRJEB90540 (https://www.ebi.ac.uk/ena/browser/view/PRJEB90540).
