# Supplementary material for: Dynamics of clonal hematopoiesis and cellular responses to stress-induced toxicity in autologous stem cell transplantation
Source: Leukemia. 2025 Dec 19;40(2):314–24. doi: 10.1038/s41375-025-02823-z (PMC12875865; doi:10.1038/s41375-025-02823-z)
Supplement: Supplementary file 1 — Supplemental Material [file 41375_2025_2823_MOESM1_ESM.docx]

**Dynamics of Clonal Hematopoiesis and Cellular Responses to Stress-Induced Toxicity in Autologous Stem Cell Transplantation**

Catarina M. Stein^1,2^, Raphael Hablesreiter^1^, Friederike Christen^1^, Pelle Löwe^1,2^, Coral Fustero-Torre^1^, Klara Kopp^1^, Benjamin N. Locher^1^, Lena Nitsch^3,4,5^, Robert Altwasser^1^, Johanna Franziska Kerschbaum^1^, Lars Bullinger^1,6^, Leif S. Ludwig^3,4^, Paulina M. Strzelecka^1^ and Frederik Damm^1,6*^

^1^Charité – Universitätsmedizin Berlin, Corporate Member of Freie Universität Berlin and Humboldt-Universität zu Berlin, and Berlin Institute of Health, Department of Hematology, Oncology, and Cancer Immunology, Berlin, Germany.

^2^Berlin School of Integrative Oncology (BSIO), Berlin, Germany.

^3^Berlin Institute of Health at Charité – Universitätsmedizin Berlin, Berlin, Germany.

^4^Max-Delbrück-Center for Molecular Medicine in the Helmholtz Association, Berlin Institute for Medical Systems Biology, Berlin, Germany.

^5^Department of Biology, Chemistry, Pharmacy, Freie Universität Berlin, Berlin, Germany.

^6^German Cancer Consortium (DKTK) and German Cancer Research Center (DKFZ), Heidelberg, Germany.

# Supplementary Methods

## Targeted sequencing: Library construction

Library preparation and sequencing were performed according to the Twist Custom Panel Hybridization Capture of DNA Libraries (Twist BioScience, San Francisco, CA, USA) protocol as previously described^1–3^. Pre-capture processing was performed using 14 ng/μl genomic DNA, which was fragmented for 17 min at 32°C and subsequently ligated to xGen™ UDI-UMI Adapters (Integrated DNA Technologies, Coralville, IA, USA) at 20°C for 15 min. Indexed libraries were purified using pre-equilibrated DNA purification beads, then subjected to a pre-capture polymerase chain reaction amplification. The amplified libraries were further purified, and quality control (QC) was performed with TapeStation D1000 ScreenTape and reagents (Agilent Technologies, Santa Clara, CA, USA) to determine fragment size. Subsequently, eight samples that passed QC (fragment size between 350-450 base pairs (bp)), with each 100 ng input, were pooled for hybridization with a Twist custom panel (**Supplementary Table S2**) for 16 h at 70°C, followed by target capture using washed streptavidin beads. Post-capture polymerase chain reaction amplification was performed, and amplified libraries were purified using pre-equilibrated DNA purification beads. QC assessment was conducted using TapeStation High Sensitivity D1000 (Agilent Technologies, Santa Clara, CA, USA), ensuring fragment size between 350 and 450 bp. Libraries were sequenced in paired-end mode (148bp+17bp+8bp+148bp) on the NovaSeq6000/X Illumina platforms (Illumina, San Diego, CA, USA).

## Targeted Sequencing: Bioinformatic analysis

The sequenced paired-end reads were processed using our in-house Snakemake^4^ pipeline^3,5–7^. Unmapped BAMs with unique molecular identifiers (UMIs) were extracted from base call files using bcl2fastq (v2.20.0.422)^8^ and fgbio’s FastqToBam (v2.0.1)^9^. The raw reads, extracted from unmapped BAMs using fgbio’s SamToFastq, were aligned to a modified GRCh38 reference genome with U2AF1 region fix^10^ using bwa mem (v0.7.18)^11^ and, subsequently, the UMI information was incorporated using picard MergeBamAlignment (v3.3.0)^12^. Consensus reads were generated using fgbio’s GroupReadsByUmi with the “-s adjacency” option and CallMolecularConsensusReads “‑M 3”. These consensus reads were again aligned to the reference genome^13^ (using bwa mem (v0.7.18)^11^) and picard MergeBamAlignment (v3.3.0)^12^) and quality filtering was performed with fgbio FilterConsensusReadsQuality, to retain reads with at least three consensus reads with default parameters. Local realignment was conducted using GATK’s (v3.8) RealignerTargetCreator and IndelRealigner^14,15^. Variant calling was executed using VarDictJava (v1.8.3)^16^ in single mode with a minimum allele frequency of 0.1%. Finally, variant calls were annotated using ANNOVAR^17^ referencing key public databases including refGen^18^, cytoBand, clinvar_20200316^19^, dbsnfp35c^20^, gnomad30_genome^21^, avsnp156^22^, cosmic92_noncoding^23^, revel^24^, nci60^25^.

## Targeted Sequencing: Somatic variant detection

Subsequent variant filtering was implemented to ensure the identification of high-confidence variants (illustrated in **Supplementary Fig. S3**). Synonymous single nucleotide variants and intronic variants were excluded for further analysis. High-quality variants were defined as followed: (i) a VAF ≥0.5%, (ii) ≥10 consensus reads supporting the alternate allele, (iii) a read depth of ≥50, (iv) a Fisher-score ≤20, and (v) a strand balance for forward and reverse reads in the range of 0.3-0.7. Variants detected in ≥20% of samples of the same sequencing lane were filtered out as likely sequencing artifacts. Germline variants and known single nucleotide polymorphisms (SNPs) were excluded based on the following criteria: (i) a population-based allele frequency ≥0.001 in the gnomAD database^21^ and a VAF between 40-60% or >90%, (ii) variants annotated in the dbSNP-database (avsnp156^22^) and a VAF between 40-60% or >90%. However, variants linked to diseases of interest (lymphoma, leukemia, myeloma, cardiovascular) were retained if they had relevant annotations (CLNDN or CLNSIG^26^) or were listed in the COSMIC (v66)^23^ database. Highly recurrent single nucleotide variants were rescued using a curated hotspot list, adapted from Feusier *et al.*^27^, with ≥3 consensus reads supporting the alternate allele. Special attention was given to variants in *ASXL1* codon G646 and G645, using a more stringent VAF cut-off of 10%, due to known artifact susceptibility^28^.

For longitudinal analyses, variants identified at any one timepoint using standard filters were subsequently tracked across all other timepoints for that patient. If a variant was not detected in filtered calls at a given timepoint, it was recovered either from raw variant calls (if VAF ≥0.1%) or by extracting base count data directly from the BAM file. Finally, all somatic mutation candidates passing the filter criteria were manually revised in the Integrative Genomics Viewer (IGV, Broad Institute, v2.11.3)^29^. Analysis was performed in R (R Foundation for Statistical Computing, Vienna, Austria) v4.2.3 using the packages tidyverse (v2.0.0)^30^, readxl (v1.4.5)^31^, writexl (v1.5.1)^32^, dplyr (v1.1.4)^33^, GenomicRanges (v1.50.2)^34^ and stringr (v1.5.1)^35^.

## Targeted sequencing: Variant validation

We validated a total of 92 samples (30% of all sequenced samples), selected randomly and without bias, by resequencing to ensure accuracy and reliability of the identified variants. All identified variants (n=81) were successfully validated demonstrating a significant strong correlation (R=0.98, p<0.001) (**Supplementary Fig. S4**). Insertions and deletions (n=12) were excluded from the validation analysis due to their inherently higher susceptibility to sequencing and alignment errors^36^.

## Targeted sequencing: Clonal fitness analysis

Clonal fitness was assessed using paired samples, as previously described^5,6,37^. Mutations with a VAF ≥0.5% at any timepoint were included in the analysis. Clonal growth over time was modeled using a sigmoid function and clones with a fitness (*s*) <−0.25/year were categorized as decreasing, clones with *s* >0.25/year as increasing, and all others as stable.

## Fluorescence activated cell sorting and analysis for single-cell experiments

Cryopreserved peripheral blood mononuclear cell (PBMC) samples were thawed, gradually diluted with Roswell Park Memorial Institute Medium + 10% fetal bovine serum (FBS) and washed with Fluorescence-activated cell sorting (FACS) buffer (1% FBS in phosphate buffered saline (PBS)) before antibody staining. Dead cells were excluded with 1:1 000 SYTOX^TM^ Blue dead cell stain (S34857, ThermoFisher Scientific, Waltham, MA, USA), residual granulocytes were excluded by staining with 1:50 PE-conjugated CD66b (305102, BioLegend, San Diego, CA, USA). Following the staining, cells were sorted directly into chilled PBS. FACS analyses were conducted on BD FACS Aria flow cytometers at the Berlin Institute of Health (**Supplementary Fig. S5**). The data was analyzed using FlowJo software v10.6.1 (BD, Franklin Lakes, NJ, USA).

## Mitochondrial single-cell ATAC sequencing (mtscATAC seq): Library construction

Adapted from the 10x Genomics scATAC-seq platform and in accordance with the protocol described by Lareau *et al.*^38^, mitochondrial single-cell Assay for Transposase-Accessible Chromatin (mtscATAC) libraries were generated using the Chromium Next GEM Single Cell ATAC Kit v2 (1000406, 10x Genomics, Pleasanton, CA, USA). To enable simultaneous intranuclear and mitochondrial access of the Tn5 transposase, additional fixation and lysis steps were incorporated^38^. First, cells were fixed with 1% formaldehyde (ThermoFisher Scientific, Waltham, MA, USA) in PBS for 10 min at room temperature. Fixation was subsequently quenched with a glycine solution (Boston Bioproducts, Milford, MA, USA) to a final concentration of 0.125 M, followed by two washes with PBS. Cells were then incubated with chilled lysis buffer for 3 min on ice, after which chilled wash buffer was added (**Supplementary Table S4**). After centrifugation, the supernatant was discarded and the cells were resuspended in 1x diluted nuclei buffer (10x Genomics, Pleasanton, CA, USA) according to the manufacturers protocol. Cells were counted and sequencing libraries were generated following the steps outlined in the Chromium Single Cell ATAC Solution user guide (CG000209, 10x Genomics, Pleasanton, CA, USA). Before, sequencing, libraries were quantified using a Qubit dsDNA HS Assay Kit (Invitrogen, Waltham, MA, USA) and a high-sensitivity DNA chip run on a Bioanalyzer 2100 system (Agilent, Santa Clara, CA, USA) (**Supplementary Fig. S6**). All libraries were sequenced on Illumina NovaSeq6000/X sequencing platforms (Illumina, San Diego, CA, USA) in a paired-end mode (100bp+8bp+16bp+100bp).

## Mitochondrial single-cell ATAC sequencing (mtscATAC seq): Preprocessing and quality control

Raw mtscATAC seq base calls were demultiplexed and aligned to a nuclear mitochondrial DNA (mtDNA) segment (NUMT)-masked hg38 reference genome using CellRanger-ATAC (v2.1.0)^39^, mkfastq and count, subsequently. Mitochondrial genotype calling was performed using the tenx-mode of the mitochondrial genome analysis toolkit (mgatk) (v0.6.1)^40^ followed by the mgatk-del-find tool of mgatk to detect possible deletion junctions in the CellRanger output. Additionally, potential multiplets were identified from the CellRanger output using Amulet (v1.1)^41^. Signac (v1.14.0)^42^ was used to create quantified matrices files for downstream analysis with Seurat (v5.1.0)^43^, derived from a common peak set (fragment length ≥20 bp and <10 000 bp).

Cells included in the analysis met the following quality control criteria: (i) mtDNA sequencing depth ≥10x, (ii) ≥2 000 ATAC-fragments, (iii) ≥50% of fragments mapped to accessibility peaks, (iv) >2 transcription start sites, and (v) a nucleosome score of <4 (**Supplementary Fig. S7**). Additionally, multiplets identified by Amulet^41^ were excluded from further analysis. Chromatin peak annotation and visualization across the human genome were performed with ChIPSeeker (v1.44.0)^44^ (**Supplementary Fig. S20**). Latent semantic indexing (LSI) was conducted by first normalizing the data using Term Frequency-Inverse Document Frequency (TF-IDF) and then applying linear dimensionality reduction through Singular Value Decomposition (SVD). Uniform manifold approximation and projections (UMAPs) using components 2 through 30 and PBMC cell-type annotations were obtained following the Seurat Dictionary Learning method^45^ for cross-modality integration. To achieve this, we utilized the Azimuth CITE-seq reference dataset labels^46^ along with publicly available 10x Genomics multiome RNA- and ATAC-seq PBMC data, which served as a bridge reference for integrating data across modalities. Gene activity scores were computed and normalized according to the Signac workflow^42^.

## Mitochondrial single-cell ATAC sequencing (mtscATAC seq): Differential expression analysis

For pseudo bulk differential peak accessibility, we used AggregateExpression() by Seurat (v5.1.0)^43^. Genes on chromosomes X and Y, hemoglobin genes, cell cycle-related genes, and commonly biased genes were excluded, and only those with more than ten counts were retained. Normalized enrichment scores (NES) are provided in **Supplementary Table S10**. Differential peak analysis was performed using DESeq2 (v1.38.3)^47^, with multiple hypothesis testing corrected using the Benjamini-Hochberg false discovery rate (FDR) adjustment. Genes with an FDR ≤0.05 were considered as differentially expressed genes (DEGs). In addition, we conducted single sample Gene Set Enrichment Analysis (ssGSEA) using GSVA (v1.46.0)^48^ to identify significantly enriched pathways across different cell types and conditions using predefined gene sets^49,50^. DEGs were ranked according to their t-statistics, and only gene sets with an FDR <0.01 were considered significantly enriched.

## Mitochondrial single-cell ATAC sequencing (mtscATAC seq): mtDNA variant calling and filtering

mtDNA variants were identified with Signac^42^ and were classified as high-confidence heteroplasmic variants if they exhibited a strand correlation >0.65 and a variance-mean ratio (VMR) >0.01. mtDNA variant calling was performed jointly across timepoints to identify shared and unique variants. Variants commonly considered artifacts (310T>C, 301A>C, 302A>C, 309C>T, 316G>C, 3109T>C, 513G>A, 3244G>A, 16126T>C, and 204T>C) were excluded from the analysis. Single cell heteroplasmy data from mgatk^40^ enabled multi-modal analysis of mtDNA genotypes and their distribution across chromatin profiles. The combined quality metrics and cell type annotation per cell can be found in **Supplementary Table S9**. The data handling was performed in R (R Foundation for Statistical Computing, Vienna, Austria) v4.2.3 with the libraries GenomicRanges (v1.50.0)^34^, future (v1.34.0)^51^, purr (v1.0.2)^52^, SeuratDisk (v0.0.0.9021)^53^, EnsDb.Hsapiens.v86 (v2.99.0)^54^, ggplot2 (v3.5.1)^55^, ggh4x (v0.2.8)^56^, lsa (v0.73.3)^57^, zoo (v1.8-12)^58^, SummarizedExperiment (v1.28.0)^59^, Matrix (v1.6-5), dplyr (v1.1.4)^33^, data.table (v1.15.4)^60^, patchwork (v1.3.0)^61^, ggalluvial (v0.12.5)^62^, reshape2 (v1.4.4)^63^, stringr (v1.5.1)^35^, ggrepel (v0.9.6)^64^, UpSetR (v1.4.0)^65^, circlize (v0.4.16)^66^, tibble (v3.2.1)^67^, tidyr (v1.3.1)^68^, fgsea (v1.24.0)^48^, ComplexHeatmap (v2.14.0)^69^ and matrixstats (v1.1.0)^70^.

# Supplementary Tables

**Supplementary Table S1:** Demographic and clinical characteristics preTx (prior to high dose chemotherapy (HDC)) of 60 patients undergoing autologous stem cell transplantation (ASCT) stratified by clonal hematopoiesis positive (CH^+^) and negative (CH^-^) patients. Continuous variables were compared using Wilcoxon rank-sum test. Categorical variables were analyzed using Fisher’s exact test or Pearson’s Chi-squared test, as appropriate.

|  | **CH^-^** n=28 | **CH^+^** n=32 | **p-value*** |
| --- | --- | --- | --- |
| **Age [years]** |  |  | **0.032** |
| Median (Q1, Q3) | 58 (55, 62) | 63 (56, 68) |  |
| Min, Max | 26, 69 | 36, 73 |  |
| **Sex** |  |  | 0.5 |
| Female | 8 (29%) | 12 (38%) |  |
| Male | 20 (71%) | 20 (63%) |  |
| **Diagnosis** |  |  | 0.6 |
| Hodgkin Lymphoma | 2 (7.1%) | 1 (3.1%) |  |
| Multiple Myeloma | 24 (86%) | 27 (84%) |  |
| Non-Hodgkin Lymphoma | 2 (7.1%) | 4 (13%) |  |
| **Disease stage [ISS/Lugano]** |  |  | 0.8 |
| I | 5 (22%) | 7 (25%) |  |
| II | 7 (30%) | 11 (39%) |  |
| III | 10 (43%) | 8 (29%) |  |
| IV | 1 (4.3%) | 2 (7.1%) |  |
| Missing data | 5 | 4 |  |
| **Cytogenetic risk** |  |  | >0.9 |
| High | 7 (39%) | 8 (40%) |  |
| Standard | 11 (61%) | 12 (60%) |  |
| Missing data | 10 | 12 |  |
| **Nr. of treatment lines received prior to HDC** |  |  | 0.9 |
| >1 | 7 (25%) | 6 (19%) |  |
| 1 | 21 (75%) | 25 (78%) |  |
| **Treatment received prior to HDC** |  |  | 0.074 |
| D-VTD | 8 (29%) | 16 (50%) |  |
| VCD | 16 (57%) | 9 (28%) |  |
| Other | 4 (14%) | 7 (22%) |  |
| **Mobilization regimen** |  |  | >0.9 |
| CP+G-CSF | 23 (82%) | 25 (78%) |  |
| G-CSF only | 1 (3.6%) | 1 (3.1%) |  |
| Missing data | 0 (0%) | 1 (3.1%) |  |
| Other chemotherapy+G-CSF | 4 (14%) | 5 (16%) |  |
| **Mobilization Plerixafor** |  |  | 0.9 |
| No Plerixafor | 18 (64%) | 21 (66%) |  |
| Plerixafor | 10 (36%) | 10 (31%) |  |
| **Mobilization status** |  |  | 0.9 |
| Good mobilizer | 18 (64%) | 21 (66%) |  |
| Poor mobilizer | 10 (36%) | 10 (31%) |  |
| **Nr. of apheresis days** |  |  | 0.6 |
| >1 | 18 (64%) | 21 (70%) |  |
| 1 | 10 (36%) | 9 (30%) |  |
| Missing data | 0 | 2 |  |
| **Total nr. of collected CD34^+^ cells [×10^6^/kgKG]** |  |  | 0.2 |
| Median (Q1, Q3) | 9.3 (7.4, 15.2) | 8.1 (6.3, 12.0) |  |
| Min, Max | 4.4, 28.4 | 4.3, 25.6 |  |
| Missing data | 0 | 2 |  |
| **HDC regimen** |  |  | >0.9 |
| BCNU/TT | 1 (3.6%) | 0 (0%) |  |
| BEAM | 3 (11%) | 4 (13%) |  |
| Melphalan only | 24 (86%) | 26 (81%) |  |
| TEAM | 0 (0%) | 1 (3.1%) |  |
| Missing data | 0 (0%) | 1 (3.1%) |  |
| **Transplanted CD34^+^ cells [in /kgKG]** |  |  | 0.3 |
| Median (Q1, Q3) | 3.67 (2.93, 4.90) | 3.21 (2.88, 4.00) |  |
| Min, Max | 1.59, 14.19 | 1.60, 12.76 |  |
| Missing data | 2 | 2 |  |
| **Hemoglobin [in g/dl]** |  |  | 0.9 |
| Median (Q1, Q3) | 11.45 (10.30, 12.35) | 11.60 (9.95, 12.65) |  |
| Min, Max | 8.10, 13.80 | 7.10, 16.20 |  |
| **Leucocytes [in T/µl]** |  |  | 0.5 |
| Median (Q1, Q3) | 6.8 (5.0, 10.4) | 6.5 (4.4, 8.9) |  |
| Min, Max | 2.8, 40.9 | 2.8, 17.1 |  |
| **Thrombocytes [in T/µl]** |  |  | 0.9 |
| Median (Q1, Q3) | 210 (173, 261) | 214 (164, 271) |  |
| Min, Max | 41, 329 | 23, 390 |  |
| **Calcium [in mmol/l]** |  |  | >0.9 |
| Median (Q1, Q3) | 2.22 (2.08, 2.37) | 2.28 (1.99, 2.37) |  |
| Min, Max | 1.94, 2.40 | 1.76, 2.50 |  |
| Missing data | 9 | 17 |  |
| **Creatinine [in mg/dl]** |  |  | 0.3 |
| Median (Q1, Q3) | 0.93 (0.77, 1.41) | 0.87 (0.75, 1.09) |  |
| Min, Max | 0.58, 3.33 | 0.59, 2.21 |  |
| Missing data | 1 | 4 |  |
| **eGFR [in ml/min]** |  |  | 0.4 |
| Median (Q1, Q3) | 79 (54, 95) | 86 (66, 97) |  |
| Min, Max | 15, 125 | 30, 119 |  |
| Missing data | 1 | 4 |  |
| **CRP [in mg/l]** |  |  | **0.004** |
| Median (Q1, Q3) | 3.0 (1.8, 9.9) | 1.2 (0.9, 2.4) |  |
| Min, Max | 0.6, 51.6 | 0.6, 11.1 |  |
| Missing data | 6 | 10 |  |
| **Hospitalization post-ASCT [days]** |  |  | 0.4 |
| Median (Q1, Q3) | 15.0 (14.0, 20.0) | 17.0 (15.0, 23.0) |  |
| Min, Max | 13.0, 46.0 | 13.0, 30.0 |  |
| Missing data | 0 | 1 |  |
| **Leucocyte recovery [days]** |  |  | 0.6 |
| Median (Q1, Q3) | 13.0 (12.0, 14.0) | 12.0 (11.0, 15.0) |  |
| Min, Max | 9.0, 24.0 | 7.0, 20.0 |  |
| Missing data | 0 | 1 |  |
| **Thrombocyte recovery [days]** |  |  | 0.7 |
| Median (Q1, Q3) | 15.0 (14.0, 18.0) | 15.0 (13.0, 17.0) |  |
| Min, Max | 11.0, 27.0 | 9.0, 28.0 |  |
| Missing data | 1 | 1 |  |
| **Response to treatment** |  |  | 0.7 |
| CR | 12 (63%) | 13 (54%) |  |
| PD | 3 (16%) | 2 (8.3%) |  |
| SD | 2 (11%) | 5 (21%) |  |
| VGPR | 2 (11%) | 4 (17%) |  |
| Missing data | 9 | 8 |  |
| *Wilcoxon rank-sum test, Pearson's Chi-squared test, Fisher's exact test, Wilcoxon rank-sum exact test  BCNU/TT= Carmustine and Thiotepa. BEAM=Carmustine, Etoposide, Cytarabine and Melphalan. CH=Clonal hematopoiesis. CP=Cyclophosphamide. CR=Complete remission. CRP=C-reactive protein. D-VTD=Daratumumab, Bortezomib, Thalidomide and Dexamethasone. eGFR=Estimated glomerular filtration rate. G-CSF=Granulocyte Colony-Stimulating Factor. IQR=Interquartile range. ISS=International staging system. PD=Progressive disease. TEAM=Thiotepa, Etoposide, Cytarabine and Melphalan. VCD=Bortezomib, Cyclophosphamide and Dexamethasone. VGPR=Very good partial remission. | | | |

**Supplementary Table S2:** List of genes and target regions covered by the custom sequencing panel (TWIST Bioscience).

| **Gene** | **Region** | **Gene** | **Region** | **Gene** | **Region** |
| --- | --- | --- | --- | --- | --- |
| *DNMT3A* | Full | *RAD21* | Full | *ETV6* | Full |
| *TET2* | Full | *STAG2* | Full | *FLT3* | Exon 6, 14-15, 20 |
| *JAK2* | Full | *CHEK2* | Full | *GATA1* | Exon 2 |
| *ASXL1* | Full | *GNAS* | Full | *GATA2* | Full |
| *SF3B1* | Full | *GNB1* | Full | *KIT* | Exon 8-11, 17 |
| *SRSF2* | Full | *ATM* | Full | *MPL* | Exon 10 |
| *TP53* | Full | *KRAS* | Full | *NPM1* | Exon 11 |
| *U2AF1* | Full | *NRAS* | Full | *PTPN11* | Full |
| *PPM1D* | Full | *WT1* | Full | *RUNX1* | Full |
| *CBL* | Full | *MYD88* | Full | *SETBP1* | Exon 4-9 |
| *IDH1* | Full | *STAT3* | Full | *NF1* | Exon 28-38 |
| *IDH2* | Full | *BRCC3* | Full | *PHF6* | Exon 3-5, 7-9 |
| *BCOR* | Full | *CALR* | Exon 8-9 | *BRAF* | Exon 15 |
| *BCORL1* | Full | *CEBPA* | Full | *NOTCH1* | Exon 26, 27, 34 |
| *EZH2* | Full | *CSF3R* | Exon 14,17 | *XPO1* | Exon 14 |

**Supplementary Table S3**: Somatic mutations in clonal hematopoiesis (CH)-associated genes detected with custom-designed targeted sequencing panels in 60 patients undergoing autologous stem cell transplantation (ASCT). VAF=Variant allele frequency.

Please see file SupplementaryTableS3.xlsx.

**Supplementary Table S4:** Composition of lysis and wash buffer used for mitochondrial single-cell ATAC sequencing.

| **Buffer** | **Concentration** | **Composition** | **Comment** | **Manufacturer** |
| --- | --- | --- | --- | --- |
| Lysis buffer | 10 mM | Tris-HCl | pH 7.4 | Sigma-Aldrich (T2194) |
|  | 10 mM | NaCl |  | Sigma-Aldrich (59222C) |
|  | 3 mM | MgCl_2_ |  | Sigma-Aldrich (M1028) |
|  | 0.1% | NP-40 |  | Thermo Fisher Scientific (28342) |
|  | 1% | BSA |  | Miltenyi Biotech (130-091-376) |
| Wash buffer | 10 mM | Tris-HCl | pH 7.4 | Sigma-Aldrich (T2194) |
|  | 10 mM | NaCl |  | Sigma-Aldrich (59222C) |
|  | 3 mM | MgCl_2_ |  | Sigma-Aldrich (M1028) |
|  | 1% | BSA |  | Miltenyi Biotech (130-091-376) |

BSA=Bovine serum albumin. MgCl_2_=Magnesium chloride. NaCl=Sodium chloride. NP-40=Nonidet P-40. pH=Potential of hydrogen. Tris-HCl=Tris(hydroxymethyl) aminomethane hydrochloride.

**Supplementary Table S5:** Occurrence of adverse events post-autologous stem cell transplantation (ASCT) stratified by clonal hematopoiesis positive (CH^+^) and negative (CH^-^) patients. Comparisons between groups were performed using Fisher’s exact test. No correction for multiple testing was applied.

|  | **CH^-^** n=28 | **CH^+^** n=32 | **p-value*** |
| --- | --- | --- | --- |
| **Complications post-ASCT** |  |  | >0.9 |
| No | 6 (21%) | 6 (19%) |  |
| Yes | 22 (79%) | 25 (78%) |  |
| **Cardiovascular complications** |  |  | 0.10 |
| No | 25 (89%) | 32 (100%) |  |
| Yes | 3 (11%) | 0 (0%) |  |
| **Infections** |  |  | >0.9 |
| No | 9 (32%) | 10 (31%) |  |
| Yes | 19 (68%) | 22 (69%) |  |
| **Kidney failure** |  |  | 0.6 |
| No | 26 (93%) | 31 (97%) |  |
| Yes | 2 (7.1%) | 1 (3.1%) |  |
| **Mucositis** |  |  | 0.6 |
| No | 22 (79%) | 23 (72%) |  |
| Yes | 6 (21%) | 9 (28%) |  |
| **Severe outcomes** |  |  | 0.3 |
| No | 25 (89%) | 31 (97%) |  |
| Yes | 3 (11%) | 1 (3.1%) |  |
| **Skin rash** |  |  | 0.3 |
| No | 25 (89%) | 31 (97%) |  |
| Yes | 3 (11%) | 1 (3.1%) |  |
| *Fisher's exact test. | | | |

**Supplementary Table S6:** Clinical characteristics of patients undergoing 1^st^ vs. 2^nd^ autologous stem cell transplantation (ASCT). Continuous variables were compared using Wilcoxon rank-sum test; categorical variables were analyzed using Fisher’s exact test.

|  | **1^st^ ASCT** n=10 | **2^nd^ ASCT** n=10 | **p-value*** |
| --- | --- | --- | --- |
| **Transplanted CD34^+^ cells [in /kgKG]** |  |  | >0.9 |
| Median (Q1, Q3) | 2.97 (2.52, 3.16) | 2.92 (2.50, 3.16) |  |
| Min, Max | 1.60, 4,90 | 2.10, 4.90 |  |
| **Hospitalization [days]** |  |  | >0.9 |
| Median (Q1, Q3) | 17.00 (15.00, 18.00) | 16.50 (16.00, 18.00) |  |
| Min, Max | 14, 23 | 15, 21 |  |
| **Leucocytes recovery [days]** |  |  | 0.2 |
| Median (Q1, Q3) | 13.00 (12.00, 15.00) | 13.00 (12.00, 13.00) |  |
| Min, Max | 12, 20 | 10, 19 |  |
| **Thrombocyte recovery [days]** |  |  | 0.5 |
| Median (Q1, Q3) | 15.50 (14.00, 18.00) | 15.00 (12.00, 17.00) |  |
| Min, Max | 14,19 | 11,21 |  |
| **Infections post-ASCT** |  |  | >0.9 |
| No | 2 (20%) | 2 (20%) |  |
| Yes | 8 (80%) | 8 (80%) |  |
| **Mucositis post-ASCT** |  |  | 0.6 |
| No | 6 (60%) | 4 (40%) |  |
| Yes | 7 (70%) | 3 (30%) |  |
| *Wilcoxon rank-sum test, Fisher's exact test. | | | |

**Supplementary Table S7:** Clinical and sample characteristics of three multiple myeloma (MM) patients undergoing autologous stem cell transplantation (ASCT) used for mitochondrial single-cell ATAC sequencing, each sampled at two timepoints (graft and Tx1_3).

|  | **MM01**  **Graft/Tx1_3** | **MM19**  **Graft/Tx1_3** | **MM25**  **Graft/Tx1_3** |
| --- | --- | --- | --- |
| **Metadata** |  |  |  |
| Age [years] | 50 | 58 | 59 |
| Sex | Female | Male | Female |
| Diagnosis | MM | MM | MM |
| Disease stage [ISS] | I | III | III |
| Other diseases | Artery hypertony/ Hypothyroidism | Artery hypertony/ Kidney deficiency/ Asthma | Kidney deficiency |
| Disease status preTx | NA | VGPR | CR |
| Previous treatment | VCD/ Radiation | VCD | D-VTD |
| Mobilization status | Good | Poor | Poor |
| Transplanted CD34^+^ cells [in /kgKG] | 2.89 | 3.01 | 2.97 |
| CH status preTx | negative | positive | positive |
| **Blood counts** |  |  |  |
| Hemoglobin [in g/dl] | 12.6/13.7 | 8.6/11.8 | 12.8/11.3 |
| Leucocytes [in T/µl] | 2.8/1.8 | 3.8/6.3 | 5.6/6.6 |
| Thrombocytes [in T/µl] | 165/99 | 255/NA | 284/257 |
| **Sample collection** |  |  |  |
| Origin | Graft/PB | Graft/PB | Graft/PB |
| Collection post-ASCT [days] | 0/161 | 0/116 | 0/122 |
| CH=Clonal hematopoiesis. CR=Complete remission. D-VTD=Daratumumab, Bortezomib, Thalidomide, Dexamethasone. ISS=International staging system. NA=Not available. PB=Peripheral blood. preTx=prior to high-dose chemotherapy. VCD=Bortezomib, Cyclophosphamide and Dexamethasone. VGPR=Very good partial remission. | | | |

**Supplementary Table S8:** Mitochondrial single-cell ATAC sequencing cell numbers and quality metrics for three multiple myeloma (MM) patients, across graft and Tx1_3 samples.

| **Parameter** | **MM01** | | **MM19** | | **MM25** | |
| --- | --- | --- | --- | --- | --- | --- |
|  | **Graft** | **Tx1_3** | **Graft** | **Tx1_3** | **Graft** | **Tx1_3** |
| Un-filtered cell numbers | 27 160 | 8 240 | 22 394 | 10 452 | 16 879 | 14 222 |
| Filtered cell numbers | 17 152 | 7 149 | 17 652 | 8 554 | 13 573 | 9 600 |
| Median read count | 11 603 | 8 476 | 8 652 | 6 501 | 6 988 | 6 089 |
| Median TSS Enrichment | 5.96 | 6.44 | 5.75 | 6.04 | 5.86 | 5.83 |
| Median Nucleosome signal | 0.38 | 0.50 | 0.31 | 0.26 | 0.41 | 0.44 |
| Median % reads in peaks | 74.46 | 76.92 | 77.72 | 79.18 | 75.44 | 72.98 |
| Median mtDNA depth | 38.51 | 35.44 | 42.72 | 28.13 | 34.01 | 20.76 |

mtDNA=mitochondrial DNA. TSS=Transcription start site.

**Supplementary Table S9:** Comprehensive metadata for each filtered sample processed with mitochondrial single-cell ATAC sequencing, including sample identifiers, quality control metrics and cell annotation data.

Please see file SupplementaryTableS9.xlsx.

**Supplementary Table S10:** Normalized gene activity counts used in pseudo-bulk Differentiated Peak Analysis (DPA), comparing graft vs. Tx1_3 samples processed with mitochondrial single-cell ATAC sequencing. Data are shown both as combined analysis across all cell types and separately for each individual cell type.

Please see file SupplementaryTableS10.xlsx.

**Supplementary Table S11:** Classification of significant enriched gene sets (p<0.01) into functional pathway groups. Gene sets were curated from KEGG, HALLMARK, and BIOCARTA databases and grouped into thematic categories based on shared biological functions.

| **Pathway Groups** | **Pathway** |
| --- | --- |
| **Tissue remodeling & structure** | KEGG_GAP_JUNCTION |
|  | HALLMARK_EPITHELIAL_MESENCHYMAL_TRANSITION |
|  | KEGG_ECM_RECEPTOR_INTERACTION |
|  | HALLMARK_APICAL_JUNCTION |
|  | KEGG_AXON_GUIDANCE |
| **Hormone signaling** | HALLMARK_ESTROGEN_RESPONSE_LATE |
|  | HALLMARK_ESTROGEN_RESPONSE_EARLY |
|  | KEGG_MATURITY_ONSET_DIABETES_OF_THE_YOUNG |
| **Cross-functional** | KEGG_VIRAL_MYOCARDITIS |
|  | KEGG_NEUROACTIVE_LIGAND_RECEPTOR_INTERACTION |
|  | HALLMARK_ANGIOGENESIS |
|  | HALLMARK_WNT_BETA_CATENIN_SIGNALING |
| **Metabolic** | KEGG_ALPHA_LINOLENIC_ACID_METABOLISM |
|  | KEGG_LINOLEIC_ACID_METABOLISM |
|  | KEGG_STARCH_AND_SUCROSE_METABOLISM |
|  | KEGG_ADIPOCYTOKINE_SIGNALING_PATHWAY |
|  | KEGG_PORPHYRIN_AND_CHLOROPHYLL_METABOLISM |
|  | HALLMARK_FATTY_ACID_METABOLISM |
| **Immune** | KEGG_SYSTEMIC_LUPUS_ERYTHEMATOSUS |
|  | KEGG_ASTHMA |
|  | KEGG_INTESTINAL_IMMUNE_NETWORK_FOR_IGA_PRODUCTION |
|  | KEGG_LEISHMANIA_INFECTION |
|  | KEGG_ANTIGEN_PROCESSING_AND_PRESENTATION |
|  | KEGG_TYPE_I_DIABETES_MELLITUS |
|  | KEGG_ALLOGRAFT_REJECTION |
|  | KEGG_GRAFT_VERSUS_HOST_DISEASE |
|  | KEGG_AUTOIMMUNE_THYROID_DISEASE |
|  | KEGG_TOLL_LIKE_RECEPTOR_SIGNALING_PATHWAY |
|  | HALLMARK_INFLAMMATORY_RESPONSE |
|  | HALLMARK_TNFA_SIGNALING_VIA_NFKB |
|  | HALLMARK_COMPLEMENT |
|  | KEGG_CYTOKINE_CYTOKINE_RECEPTOR_INTERACTION |
|  | HALLMARK_IL2_STAT5_SIGNALING |
|  | HALLMARK_INTERFERON_ALPHA_RESPONSE |
|  | KEGG_RIG_I_LIKE_RECEPTOR_SIGNALING_PATHWAY |
|  | KEGG_JAK_STAT_SIGNALING_PATHWAY |
|  | HALLMARK_INTERFERON_GAMMA_RESPONSE |
|  | HALLMARK_ALLOGRAFT_REJECTION |
|  | KEGG_NATURAL_KILLER_CELL_MEDIATED_CYTOTOXICITY |
|  | BIOCARTA_MHC_PATHWAY |
| **Cell cycle & apoptosis** | KEGG_MAPK_SIGNALING_PATHWAY |
|  | HALLMARK_MITOTIC_SPINDLE |
|  | KEGG_P53_SIGNALING_PATHWAY |
|  | HALLMARK_APOPTOSIS |
|  | HALLMARK_P53_PATHWAY |
|  | KEGG_CELL_CYCLE |
|  | HALLMARK_G2M_CHECKPOINT |
|  | HALLMARK_E2F_TARGETS |
| **OxPhos** | HALLMARK_OXIDATIVE_PHOSPHORYLATION |
| **Stress response** | HALLMARK_UNFOLDED_PROTEIN_RESPONSE |
|  | HALLMARK_DNA_REPAIR |
|  | HALLMARK_HYPOXIA |
|  | HALLMARK_UV_RESPONSE_UP |
| **Protein synthesis** | KEGG_RIBOSOME |
|  | HALLMARK_PROTEIN_SECRETION |
| **Oncogenic** | HALLMARK_MYC_TARGETS_V1 |
|  | HALLMARK_MTORC1_SIGNALING |
|  | KEGG_HEDGEHOG_SIGNALING_PATHWAY |
|  | HALLMARK_NOTCH_SIGNALING |
|  | HALLMARK_KRAS_SIGNALING_DN |
| **Differentiation** | HALLMARK_MYOGENESIS |
|  | KEGG_BASAL_CELL_CARCINOMA |

# Supplementary Figures

**Supplementary Figure S1:** Flow diagram of inclusion and exclusion of patients undergoing autologous stem cell transplantation (ASCT).


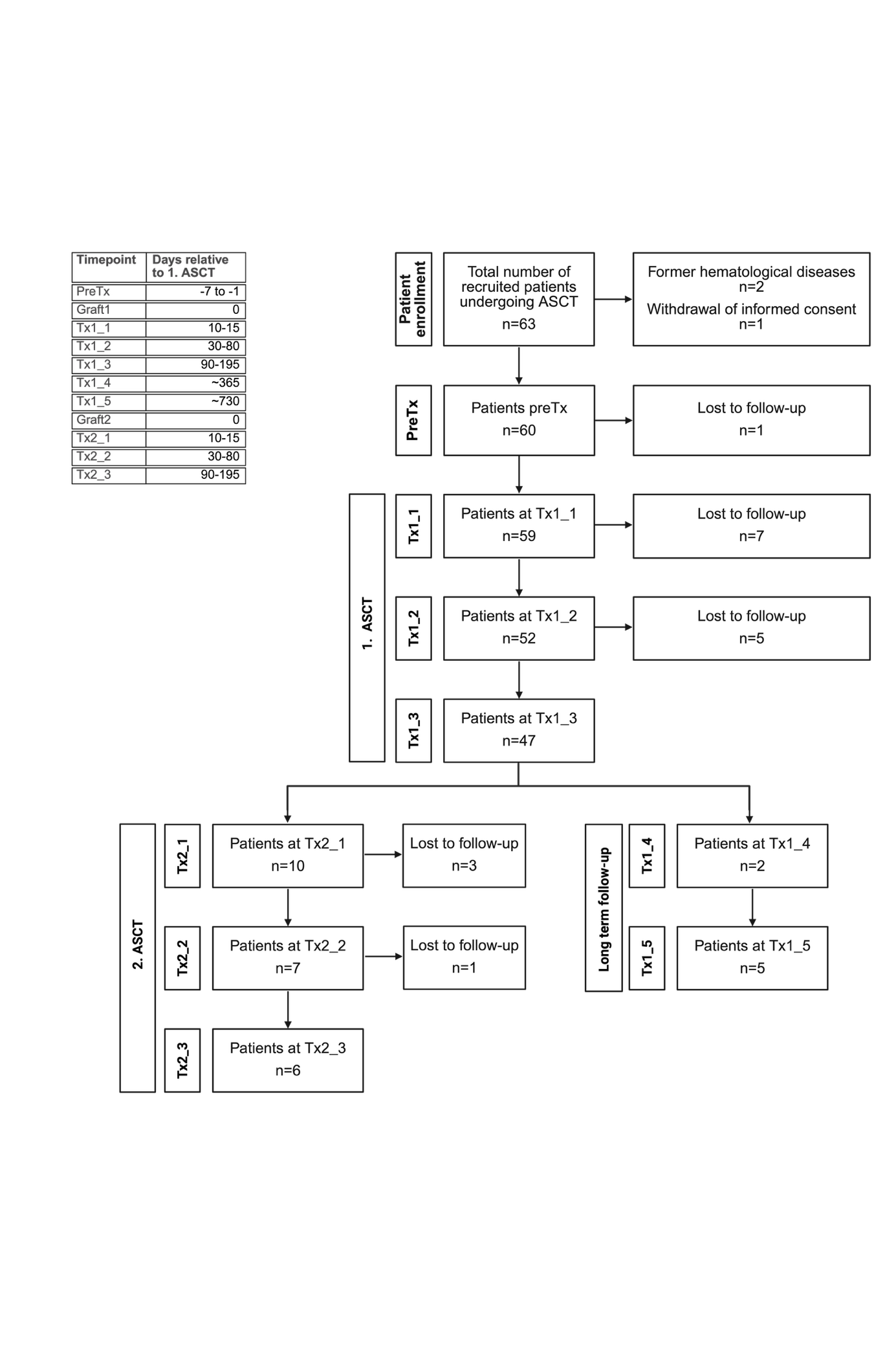


**Supplementary Figure S2:** Density diagram for targeted sequencing samples across sequencing runs (median target coverage=1,796x). Sample counts/run are shown in grey.

**
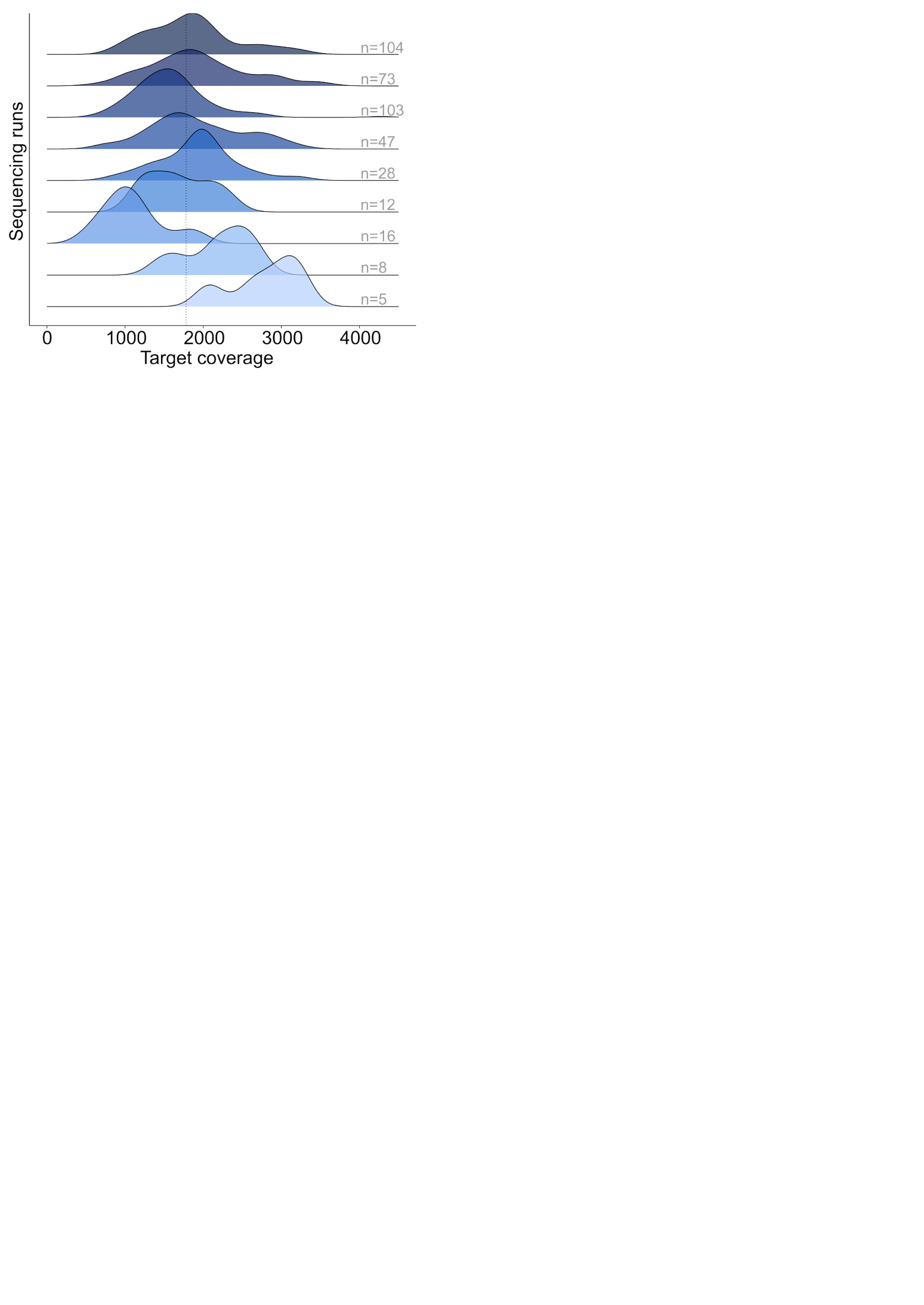
**

**Supplementary Figure S3:** Schematic overview of the filtering strategy applied to somatic genomic DNA (gDNA) variants. AF=Allele frequency. IGV=Integrative Genomics Viewer. SNP=Single nucleotide polymorphism. SNV=Single nucleotide variant. VAF=Variant allele frequency.

**
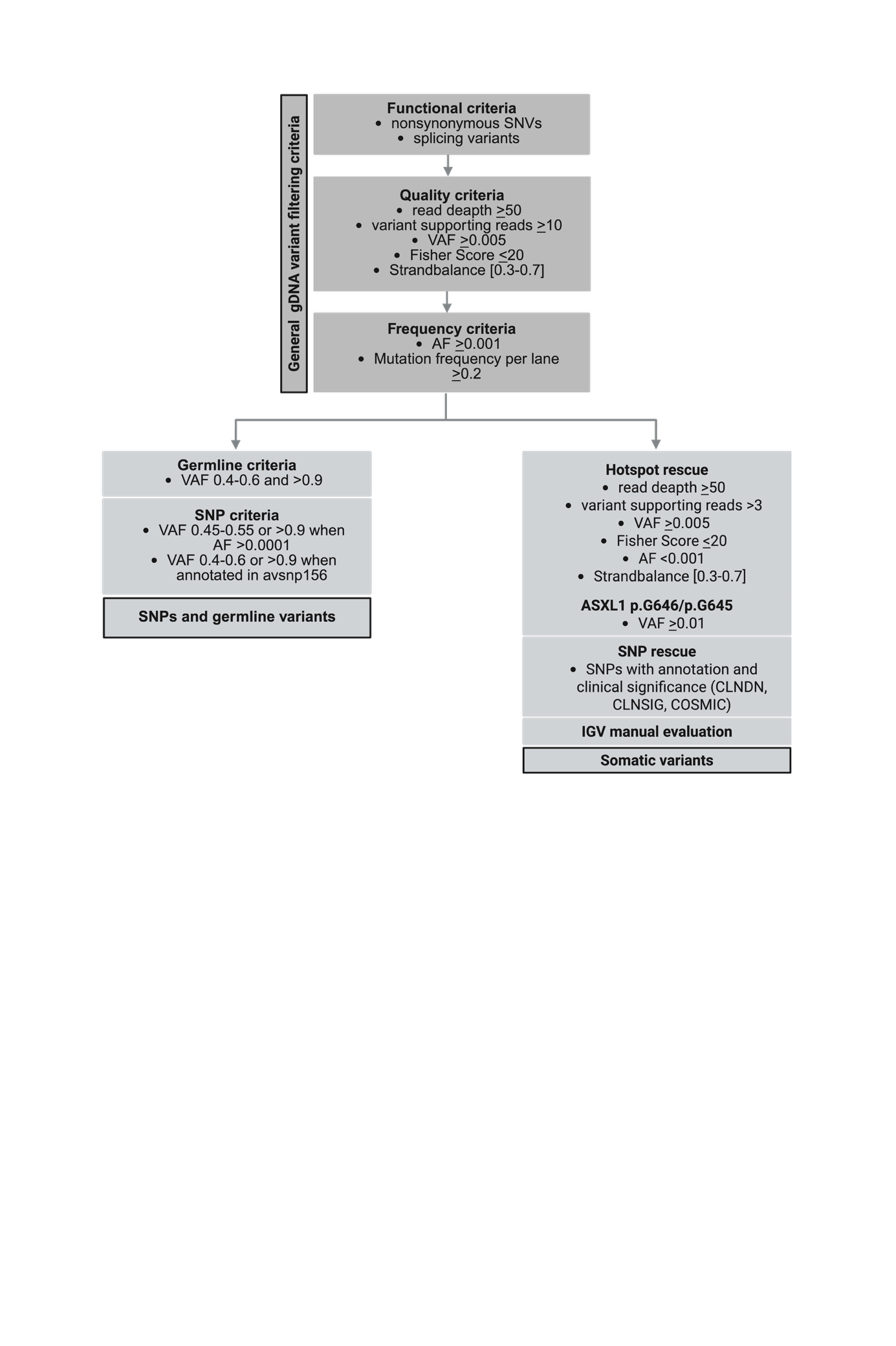
**

**Supplementary Figure S4:** Correlation plot of initial vs. validated variant allele frequencies (VAFs) (n=81) across 47 samples. Insertions and deletions (n=12) and mutation-free samples (n=45) were excluded. Pearson correlation coefficient (R) and p-value were calculated using a two-sided Pearson correlation test.

**
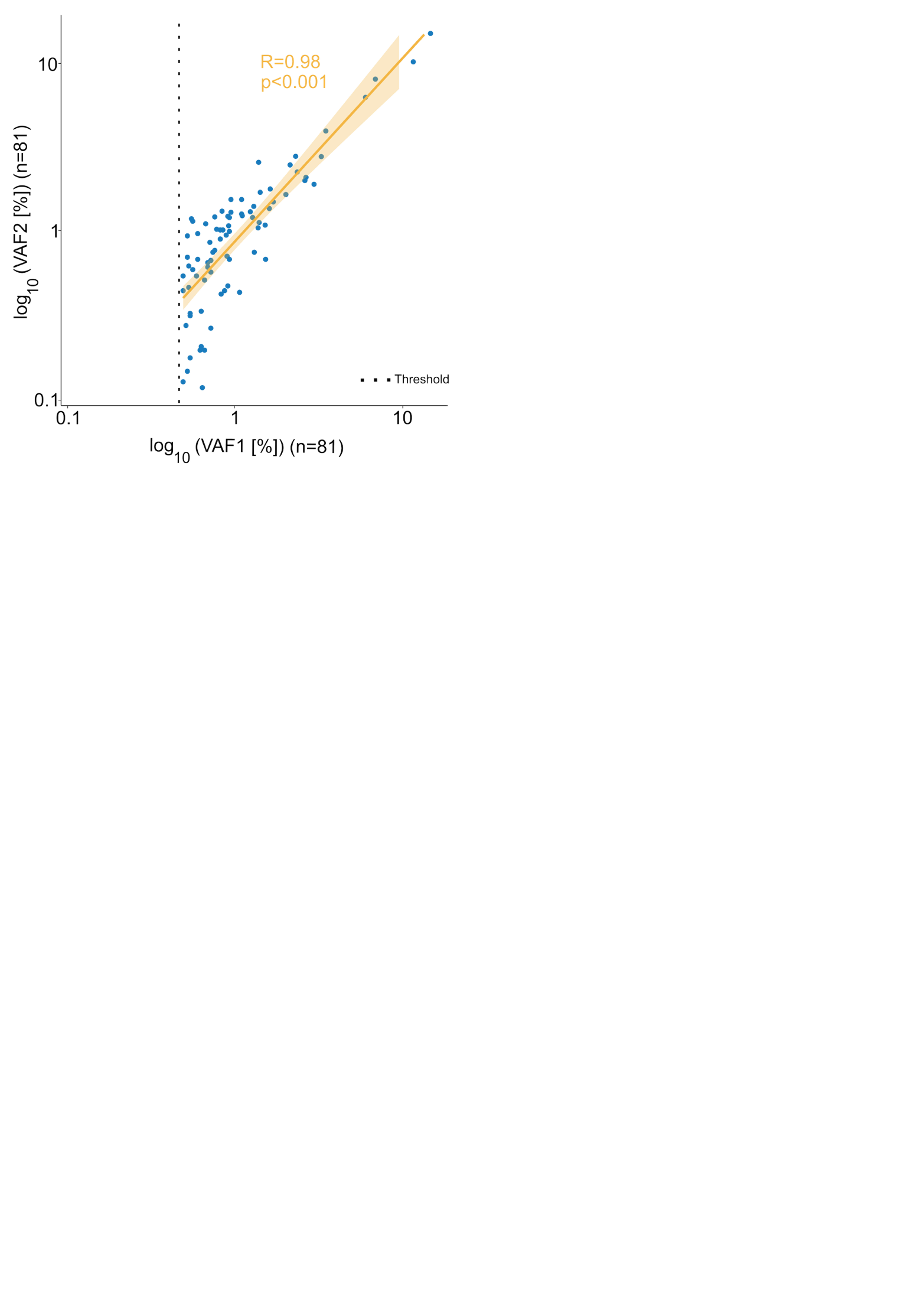
**

**Supplementary Figure S5:** Flow cytometry cell sorting strategy for mitochondrial single-cell ATAC sequencing. **a)** Gating scheme for Fluorescence activated cell sorting (FACS). Human peripheral blood mononuclear cells are stained with a live or dead cell marker (SYTOX^TM^ Blue) and anti-CD66b to exclude granulocytes. **b-g)** FACS panels from three multiple myeloma (MM) patients, each at two timepoints: graft and Tx1_3; MM01 Graft (b); MM01 Tx1_3 (c); MM19 Graft (d); MM19 Tx1_3 (e); MM25 Graft (f); MM25 Tx1_3 (g). FSC=Forward scatter. PE=Phycoerythrin. SSC=Side scatter.

**
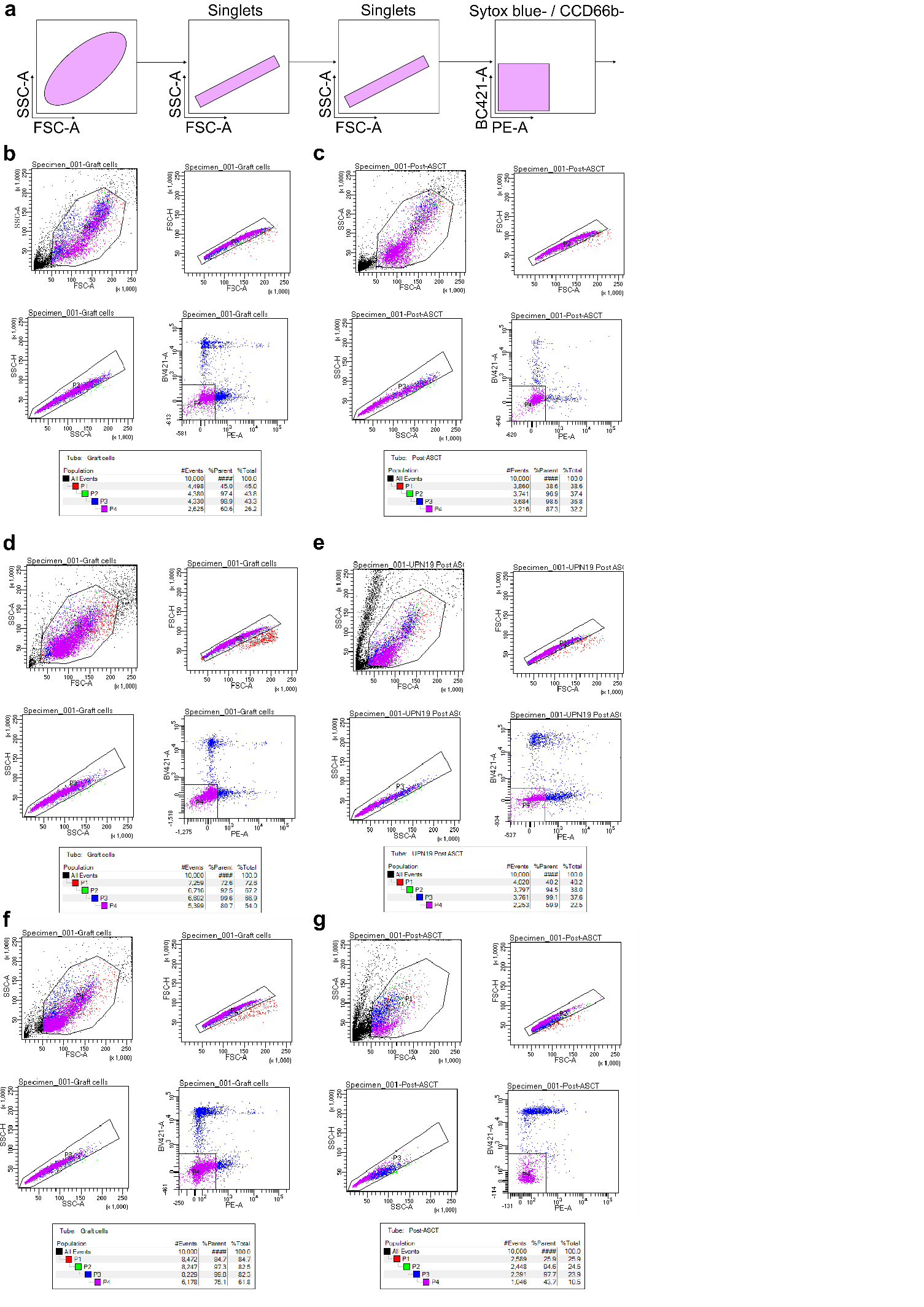
**

**Supplementary Figure S6:** Fragment size distribution of mitochondrial single-cell ATAC sequencing libraries from three multiple myeloma (MM) patients, each at two timepoints: graft and Tx1_3. Libraries were run on a high-sensitivity DNA chip and the Agilent Bioanalyzer 2100 system. bp=base pairs. FU=Fluorescence units.

**
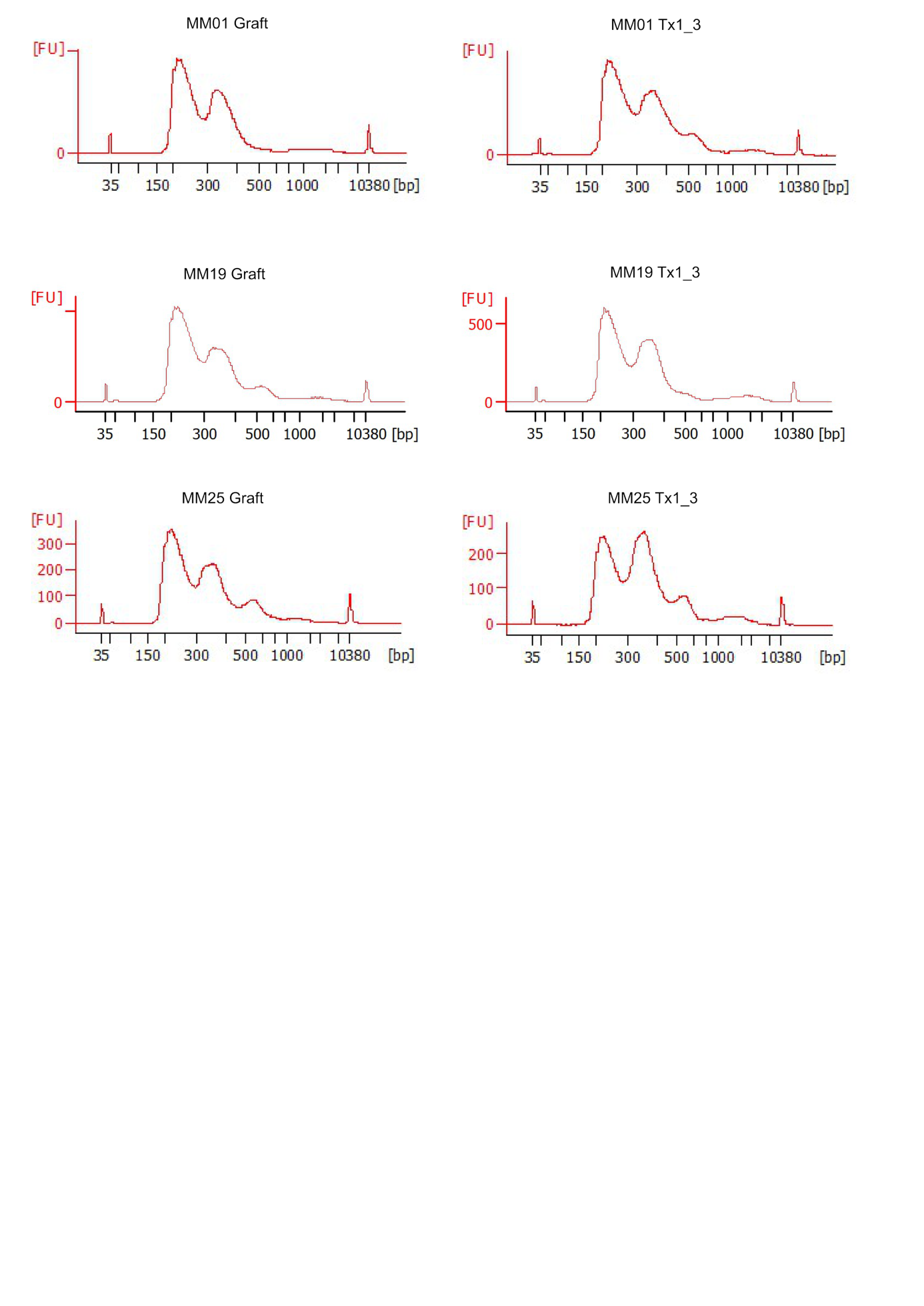
**

**Supplementary Figure S7:** Mitochondrial single-cell ATAC sequencing quality metrics from three multiple myeloma (MM) patients, each at two timepoints: graft and Tx1_3. **a)** Scatter plots showing the relationship between transcription start site (TSS) enrichment and number of fragments per cell across the different samples. Summary quality metrics are stated above. **b)** Histogram displaying the fragment length distribution across all samples. **c)** Line plot illustrating mean TSS enrichment as a function of distance from the TSS. bp=Base pairs. Frags=Fragments.


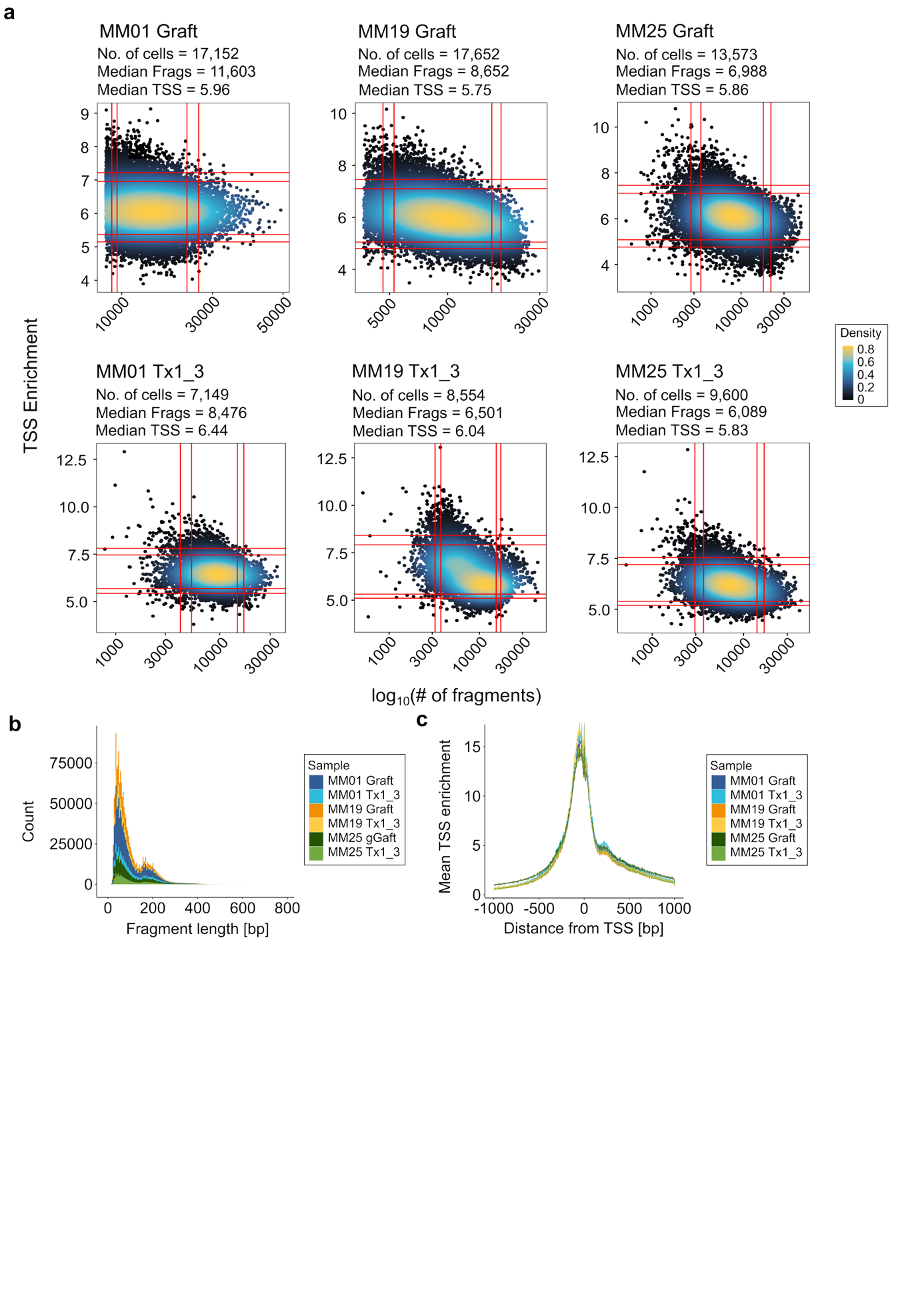


**Supplementary Figure S8:** Mutation co-occurrence in patients with multiple mutations. The size of each square reflects the frequency of co-occurrences.


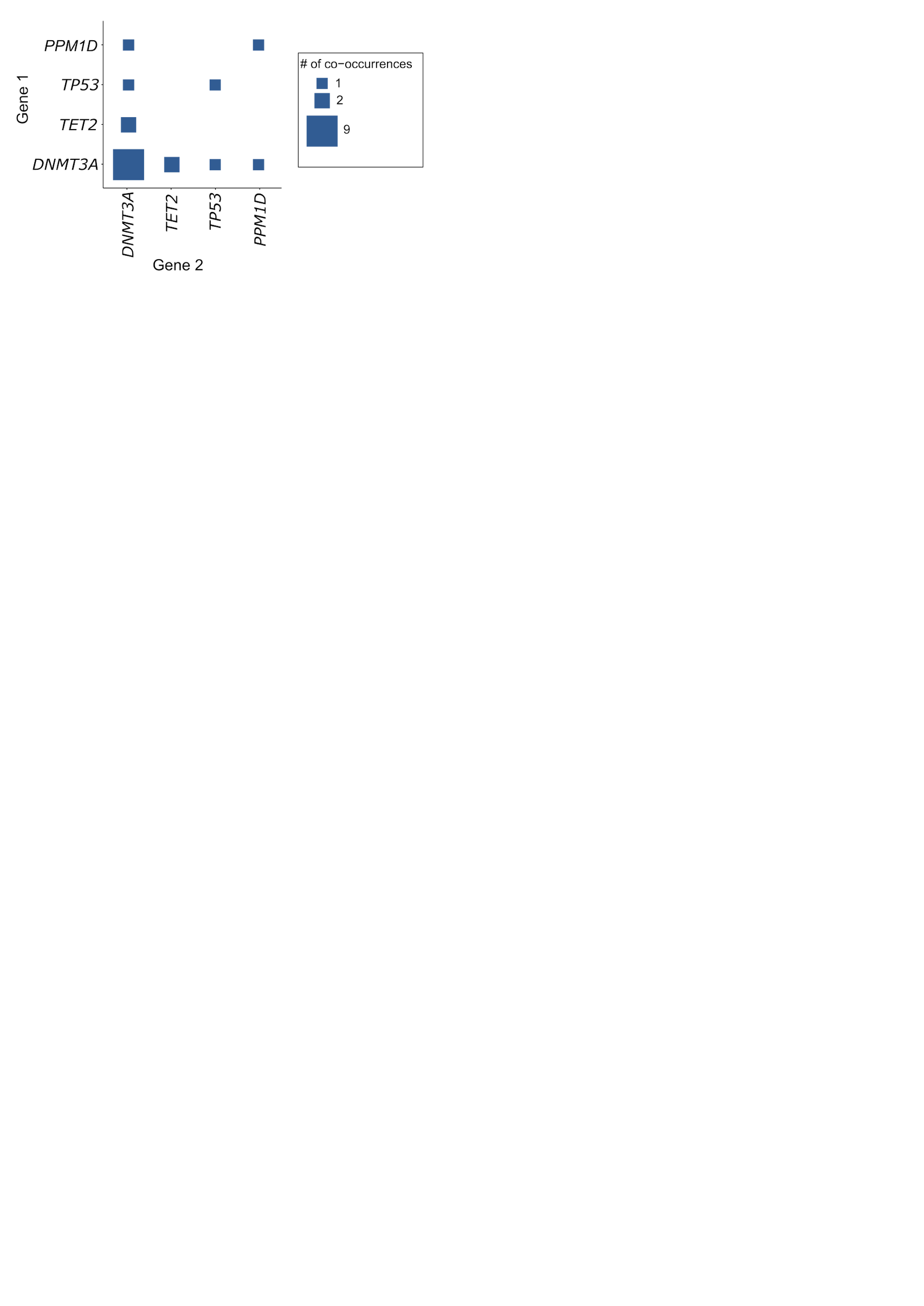


**Supplementary Figure S9:** Clinical parameters collected preTx for 60 patients undergoing autologous stem cell transplantation stratified by clonal hematopoiesis positive (CH^+^) and negative (CH^-^) patients. Violin plots comparing **a)** hospitalization duration, **b)** time to leukocyte recover >1/nl, **c)** time to thrombocyte recovery >20/nl between CH^+^ (orange) and CH^-^ (blue) patients. **d)** Violin plots of selected laboratory parameters comparing CH^+^ (orange) and CH^-^ (blue) patients. **p<0.01, Wilcoxon rank-sum test. The number of patients per condition is given in brackets. CRP=C-reactive protein. d=Day. eGFR=Estimated glomerular filtration rate.


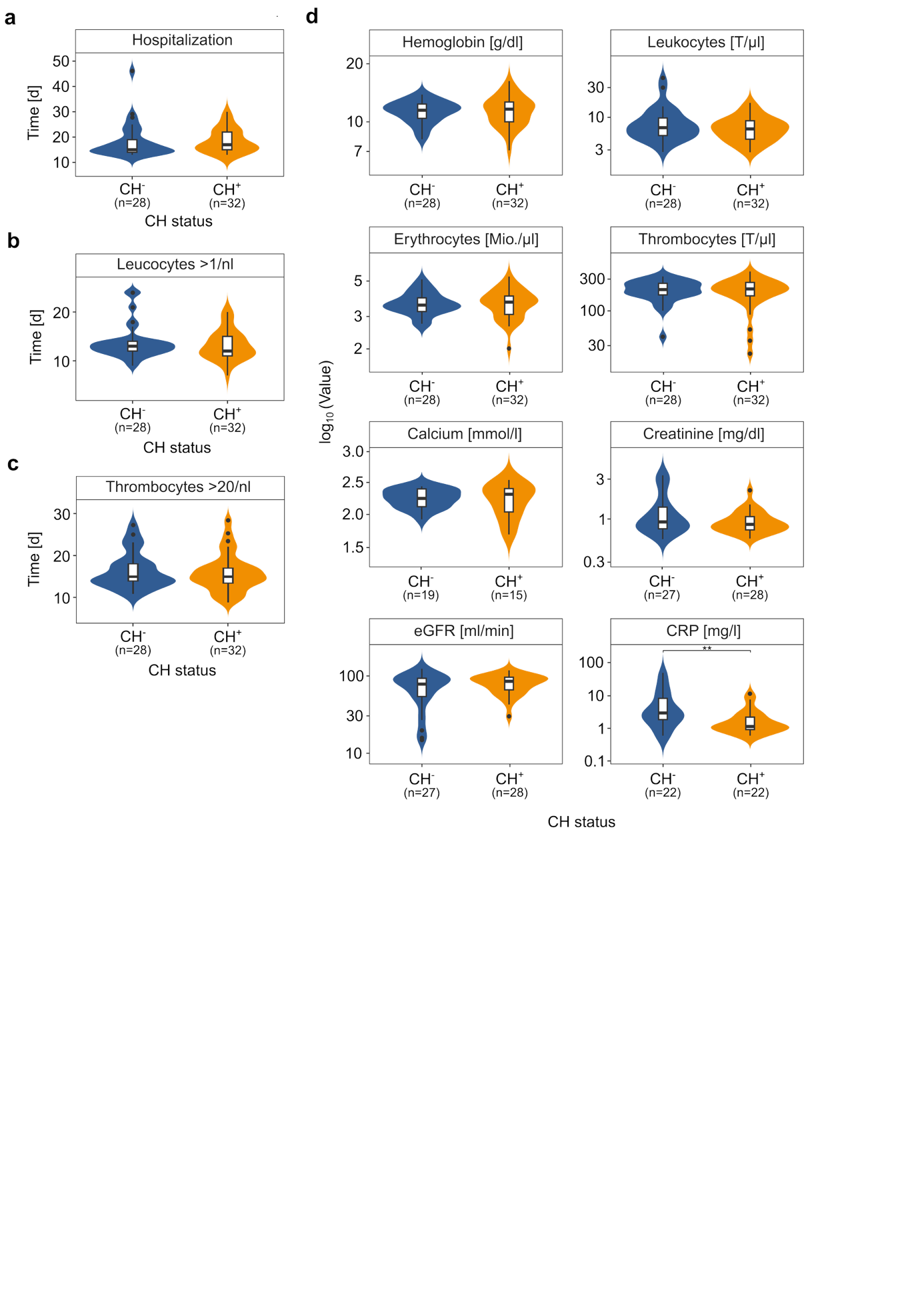


**Supplementary Figure S10:** Longitudinal sampling timeline of 51 patients following autologous stem cell transplantation (ASCT). Patient ID is represented on the y-axis, while the x-axis denotes collection time relative to ASCT (d0). The different timepoints include preTx, and sequential post-treatment samples for the 1^st^ ASCT (Graft1, Tx1_1, Tx1_2, and Tx1_3), 2^nd^ ASCT (Graft2, Tx2_1, Tx2_2, and Tx2_3), and long-term follow up (Tx1_4, Tx1_5). d=Day. PB=Peripheral blood.


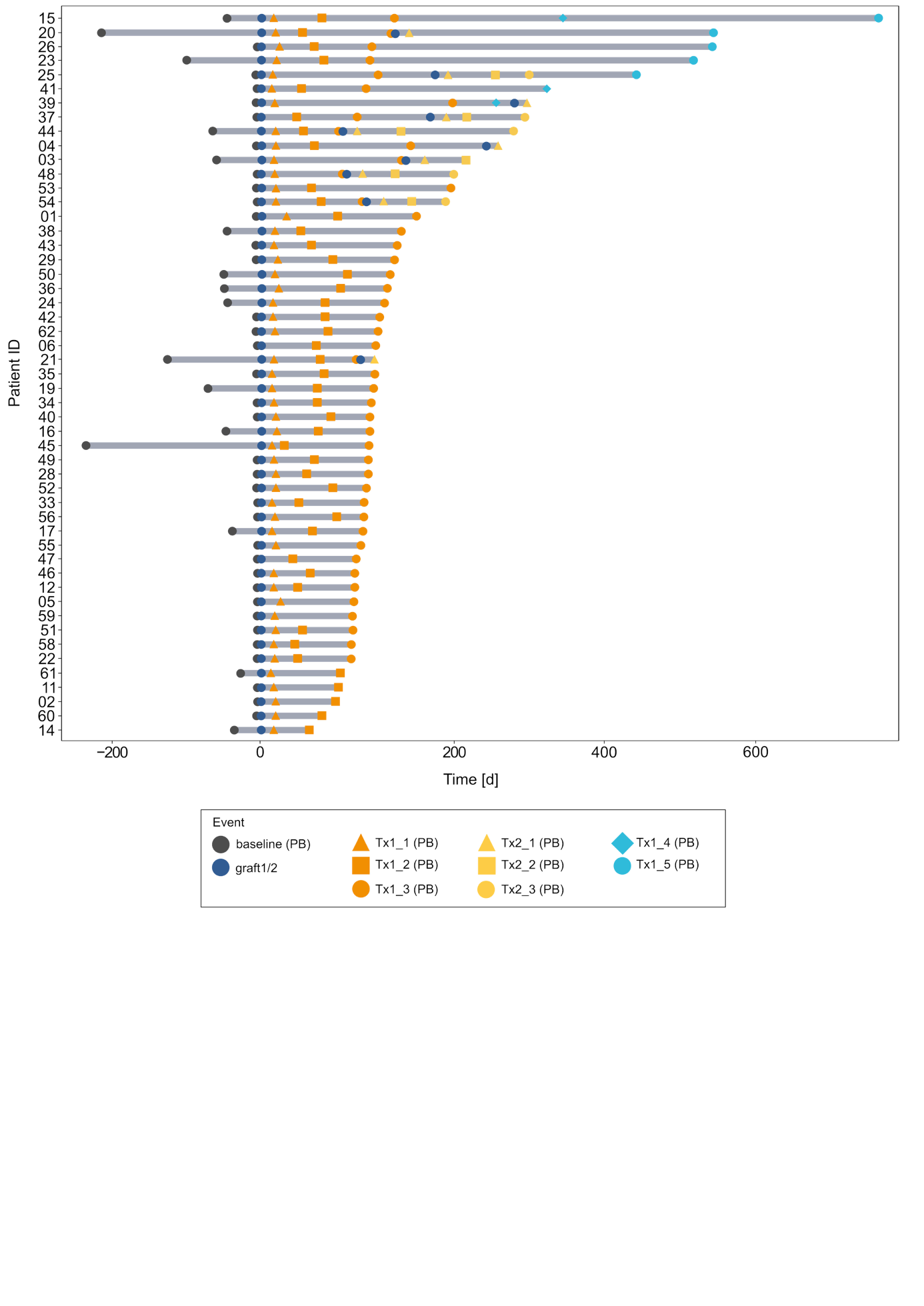


**Supplementary Figure S11:** Bar plot showing the proportion of CH-positive (CH^+^, orange) and negative (CH^-^, blue) patients undergoing autologous stem cell transplantation at each timepoint, with patient counts indicated above the bars.

**
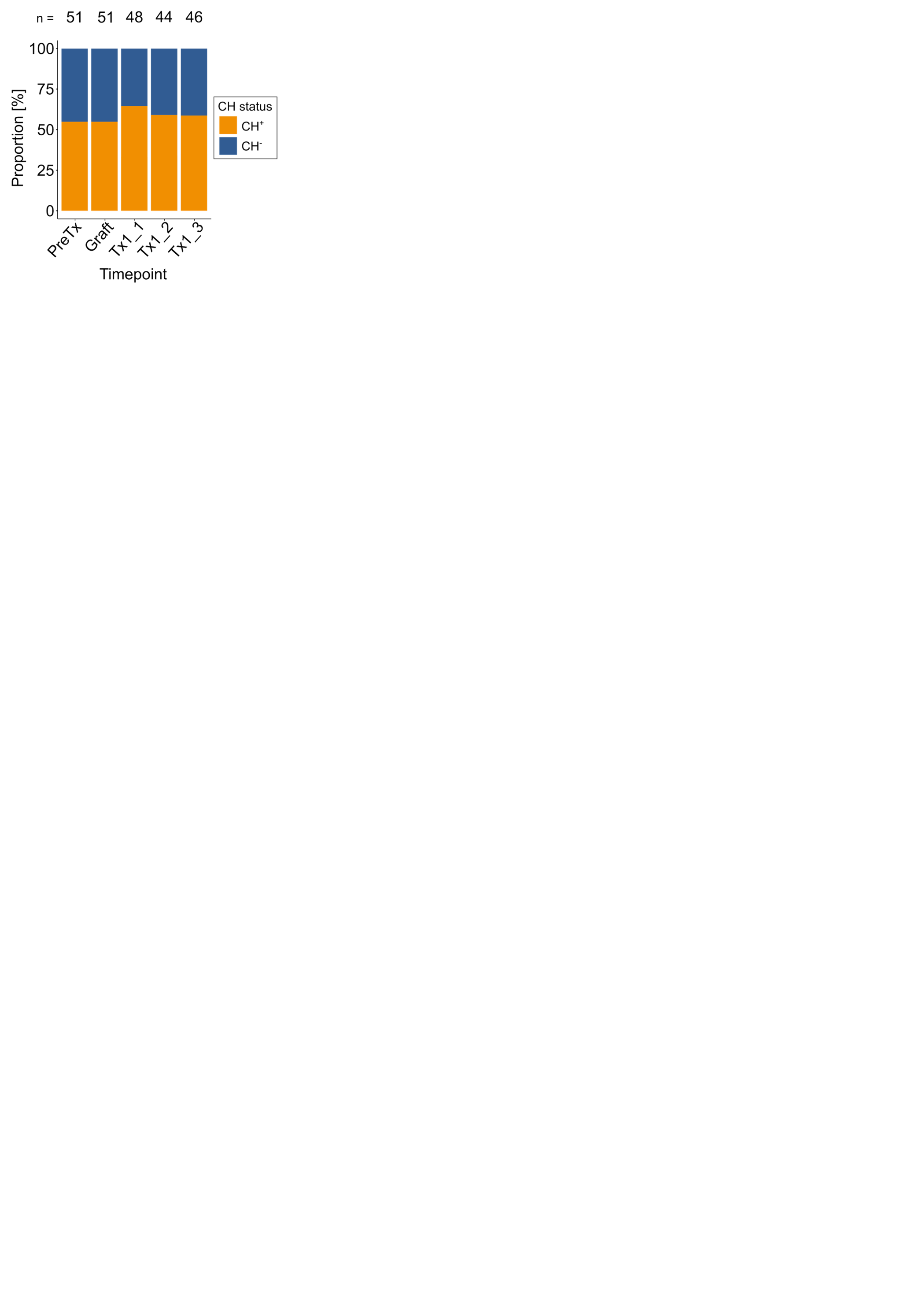
**

**Supplementary Figure S12:** Comparison of selected blood counts between clonal hematopoiesis positive (CH^+^, orange) and negative (CH^-^, blue) patients undergoing autologous stem cell transplantation across timepoints from graft to Tx1_3. CRP=C-reactive protein. eGFR=Estimated glomerular filtration rate.

**
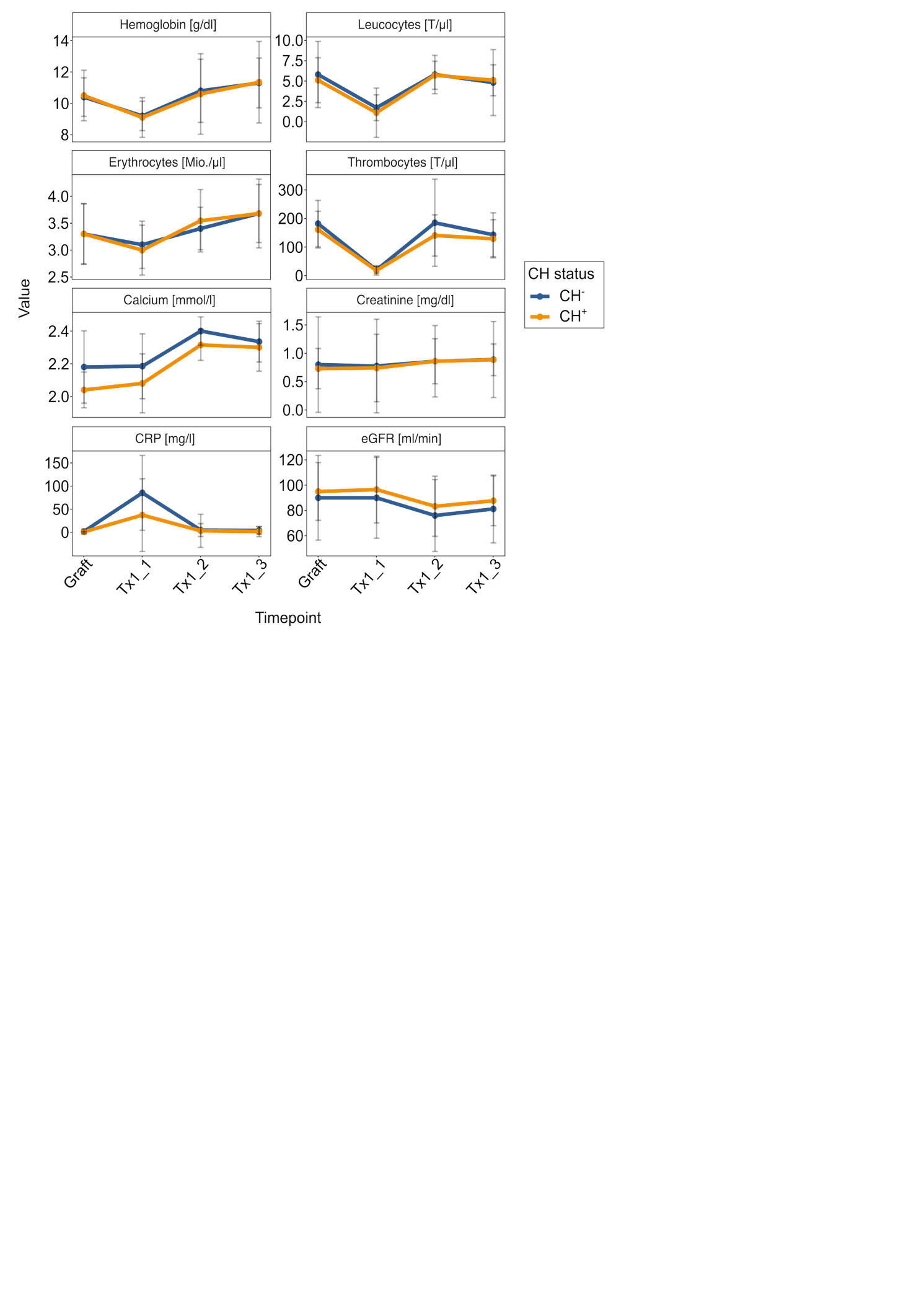
**

**Supplementary Figure S13:** Clonal dynamics in patients with and without leukopenia. **a)** Relative VAF changes over time in patients without leukopenia, from graft to Tx1_3. Colors represent clonal fitness (*s*) categories: increasing (*s* >0.25/year), decreasing (*s* <-0.25/year), and stable (−0.25≥ *s* ≤0.25/year). **b)** Boxplot of clonal fitness of patient without leukopenia from graft to Tx1_3, colored by mutated gene. **c)** Relative VAF changes over time in patients with leukopenia, from graft to Tx1_3. Colors represent clonal fitness (*s*) categories: increasing (*s* >0.25/year), decreasing (*s* <-0.25/year), and stable (−0.25≥ *s* ≤0.25/year). **d)** Boxplot of clonal fitness of patient with leukopenia from graft to Tx1_3, colored by mutated gene. d=Day. VAF=Variant allele frequency.

**
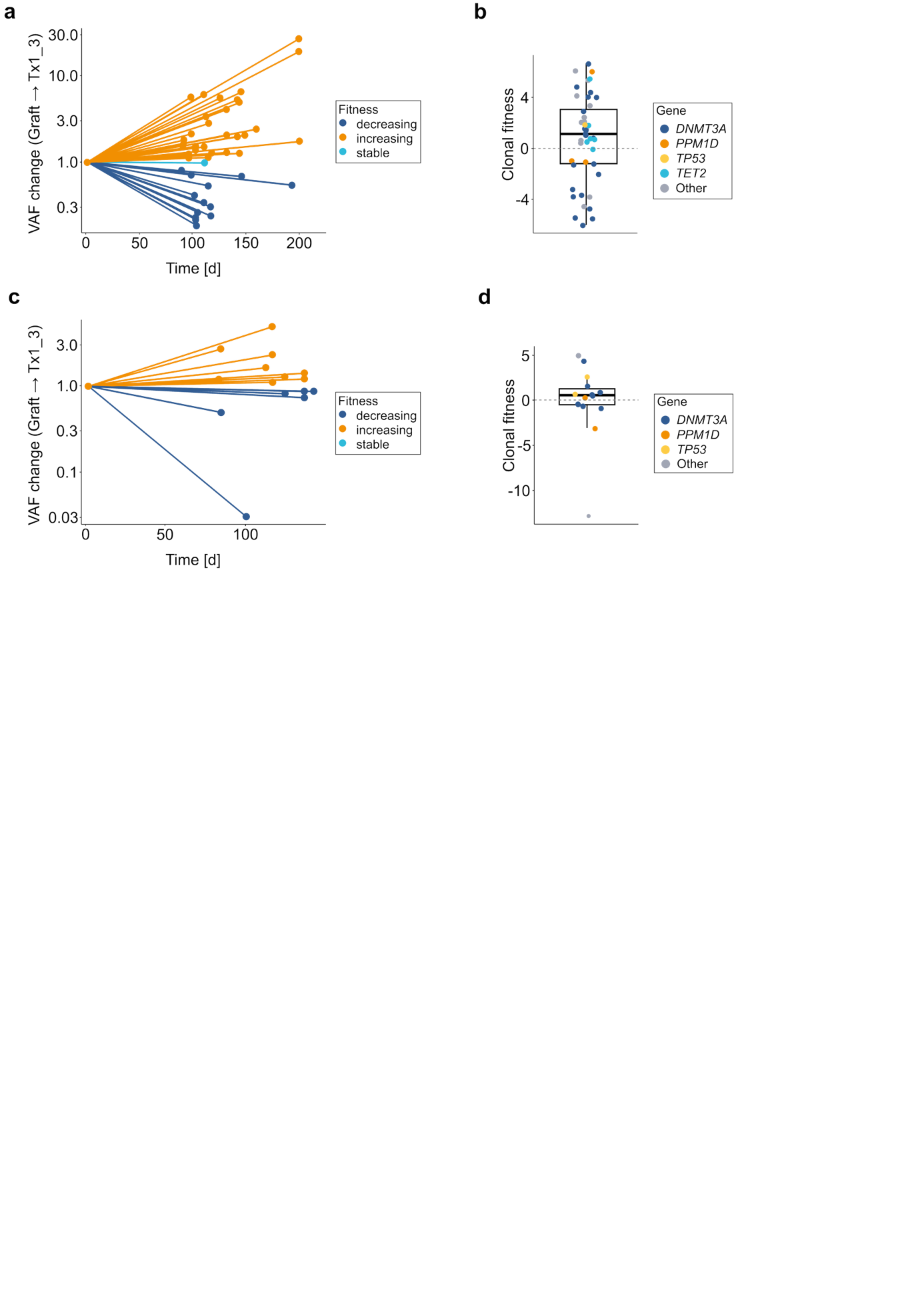
**

**Supplementary Figure S14:** Clonal dynamics of somatic mutations post-autologous stem cell transplantation per patient. Variant allele frequencies (VAFs) of somatic mutations detected in both graft and Tx1_3 samples are shown for each patient. Lines represent individual mutations and are color-coded by the corresponding mutated gene. d=Day.

**
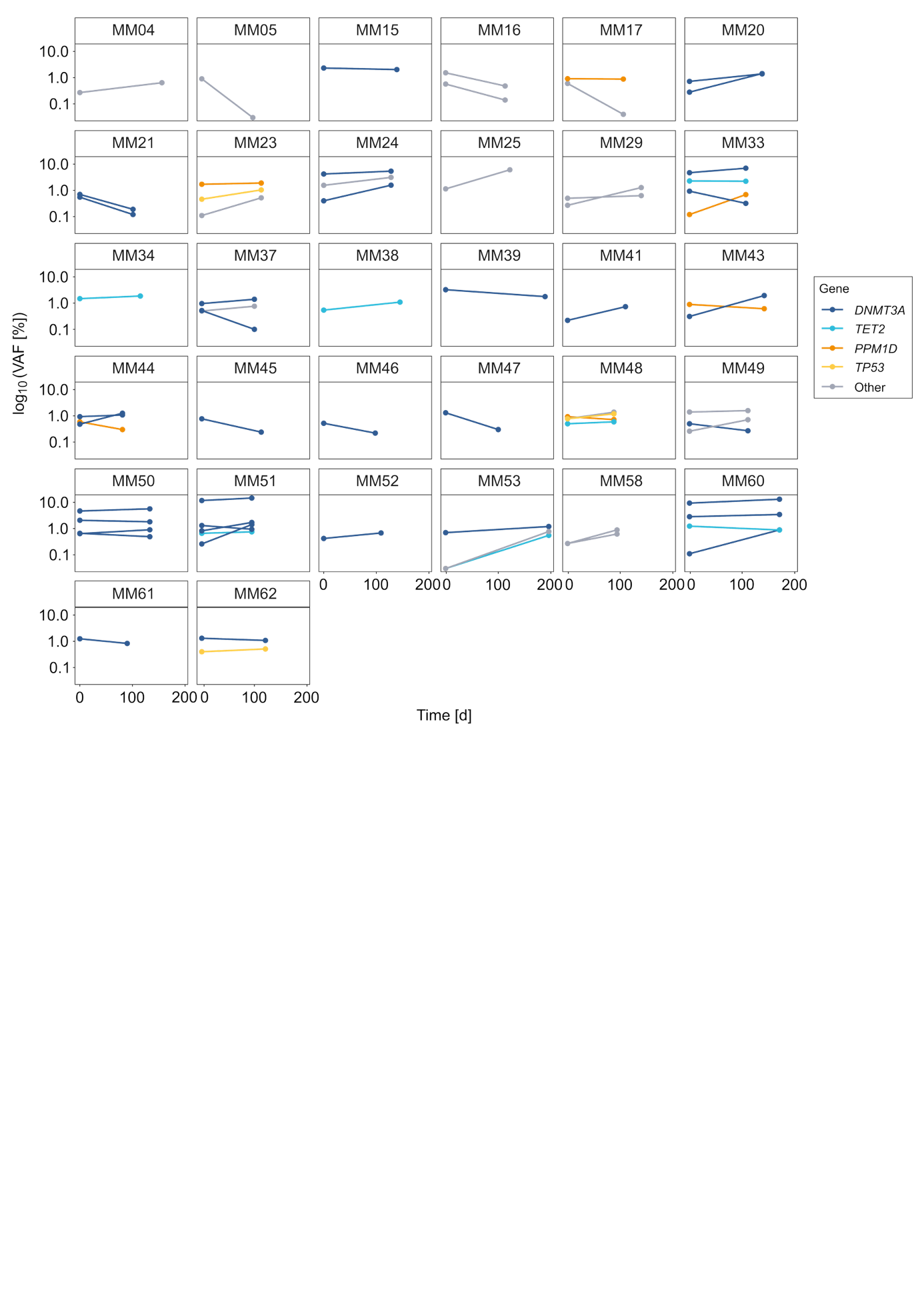
**

**Supplementary Figure S15:** Clonal dynamics of somatic mutations post-autologous stem cell transplantation per patient. Variant allele frequencies (VAFs) of somatic mutations detected in graft, Tx1_1 and Tx1_3 samples are shown for each patient. Lines represent individual mutations and are color-coded by the corresponding mutated gene. d=Day.

**
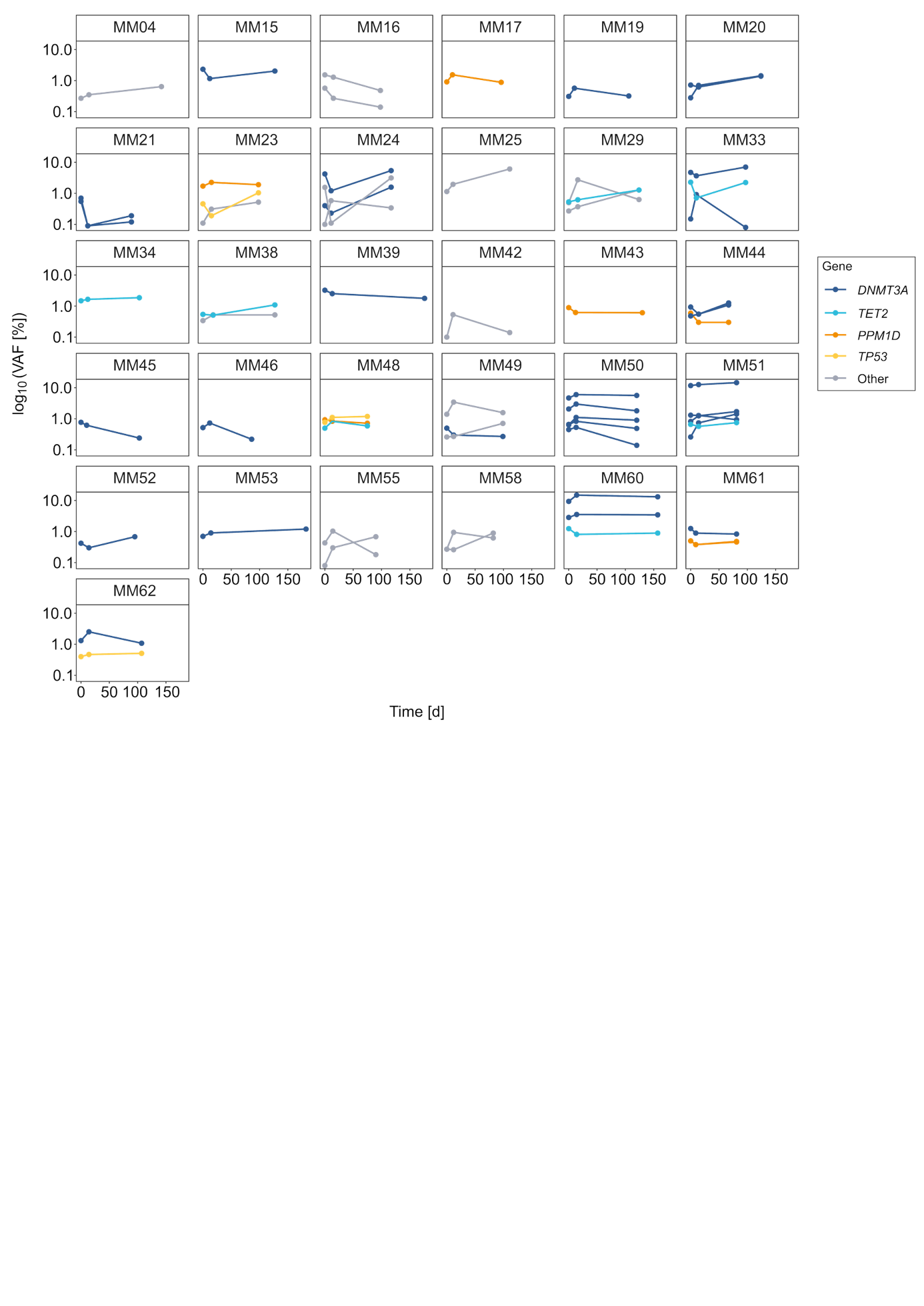
**

**Supplementary Figure S16:** Clonal dynamics between 1^st^ and 2^nd^ autologous stem cell transplantation (ASCT). **a)** Stacked bar plots showing the distribution of mutated genes at preTx, Tx1_3 and Tx2_3 in seven patients. Genes with a proportion >10% are highlighted and color-coded in the legend. **b)** Clonal dynamics of somatic mutations in 5/7 patients undergoing 1^st^ and 2^nd^ ASCT. Variant allele frequencies (VAFs) of detected somatic mutations are shown per patient. Lines represent individual mutations and are color-coded by the corresponding mutated gene. d=Day.


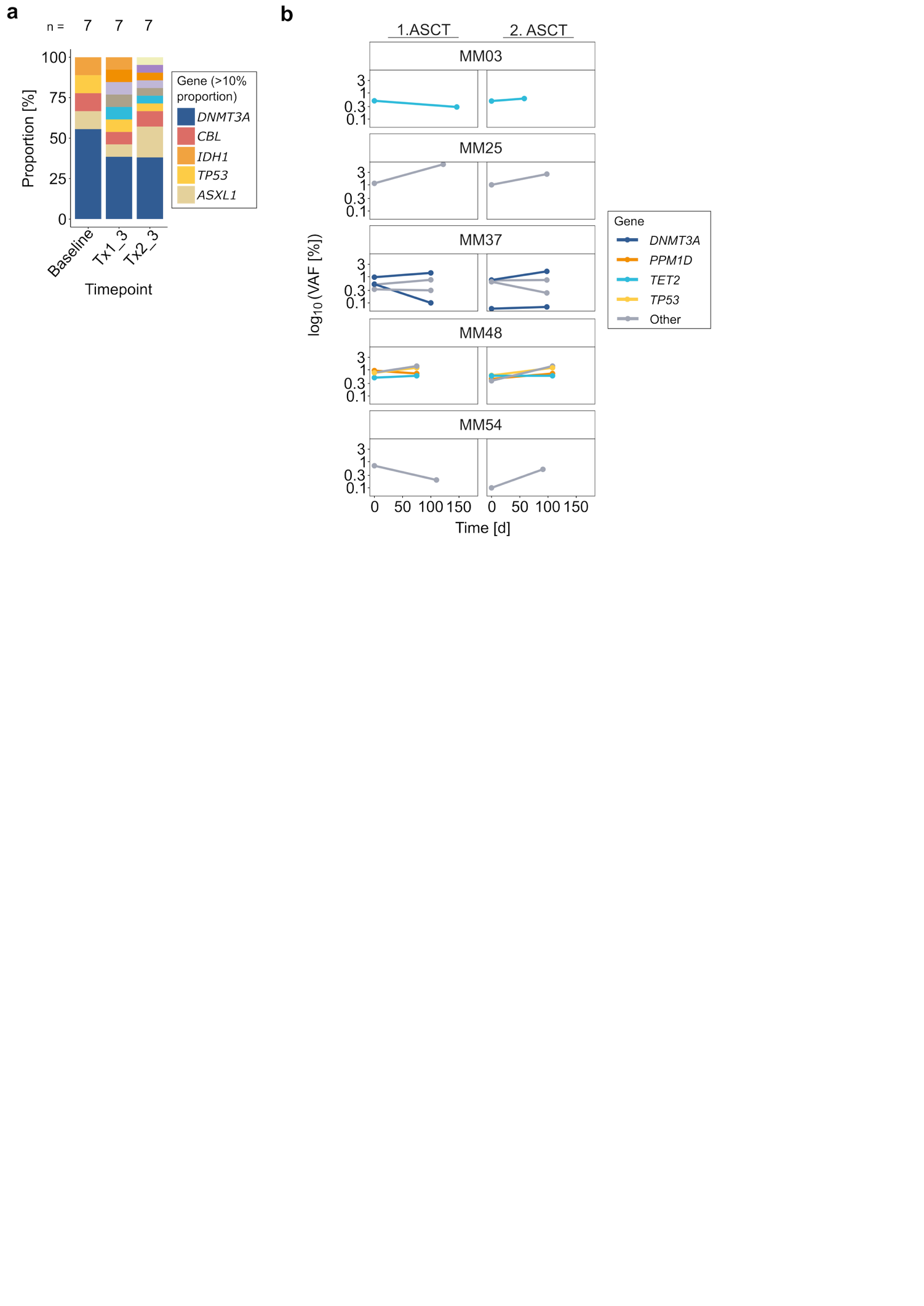


**Supplementary Figure S17:** Correlation of somatic mutations found in graft products from 1^st^ (graft1) and 2^nd^ (graft2) autologous stem cell transplant. Encircled squares indicate rescued mutations in the respective sample. VAF=Variant allele frequency.


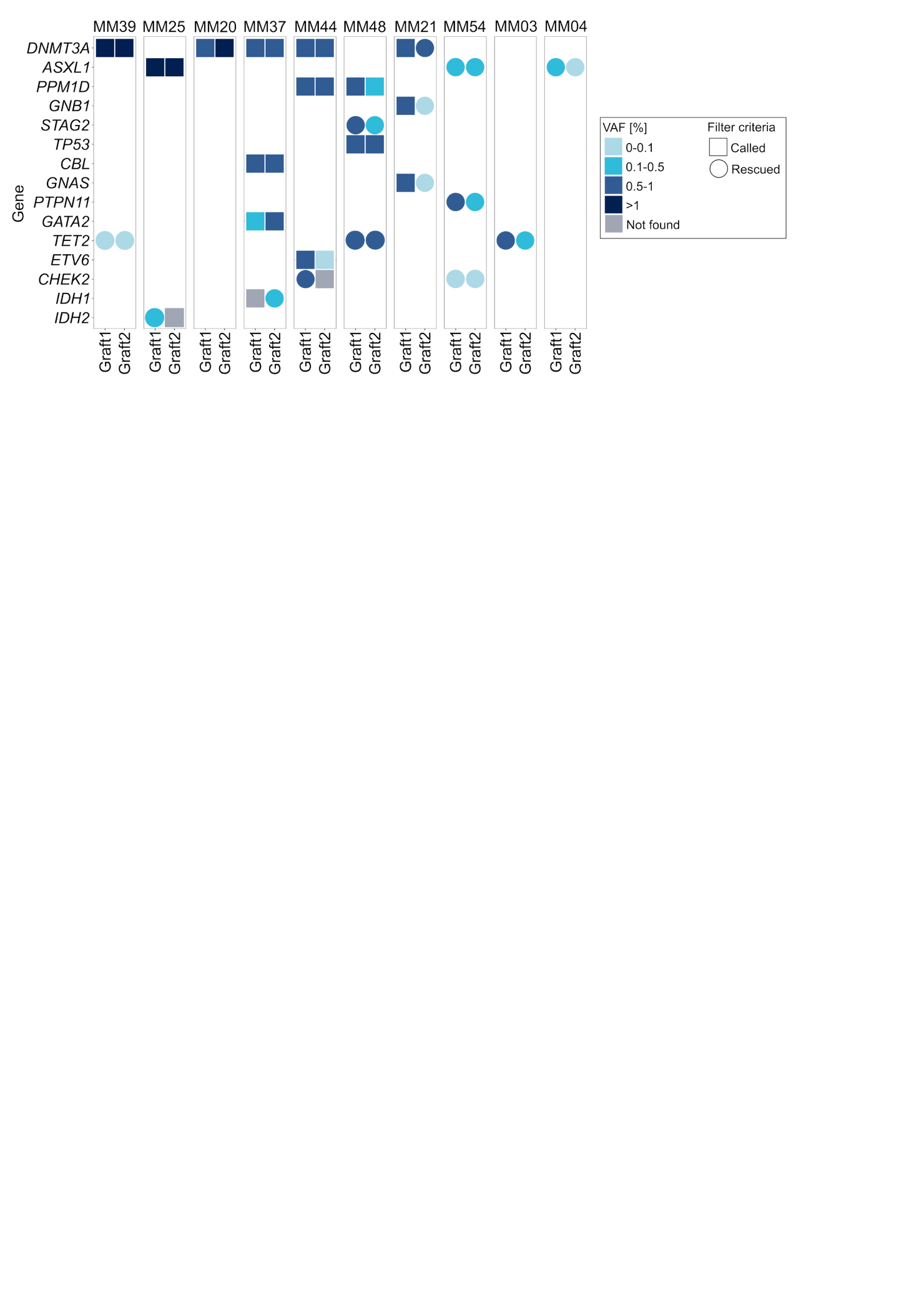


**Supplementary Figure S18:** Long-term clonal dynamics post-autologous stem cell transplantation (ASCT). **a)** Stacked bar plots showing the distribution of mutated genes at preTx, Tx1_3, Tx1_4 and Tx1_5 in seven patients. **b)** Long-term clonal dynamics of somatic mutations post-ASCT (preTx to Tx1_4/Tx1_5). Variant allele frequencies (VAFs) of somatic mutations detected are shown per patient. Lines represent individual mutations and are color-coded by the corresponding mutated gene. d=Day.

**
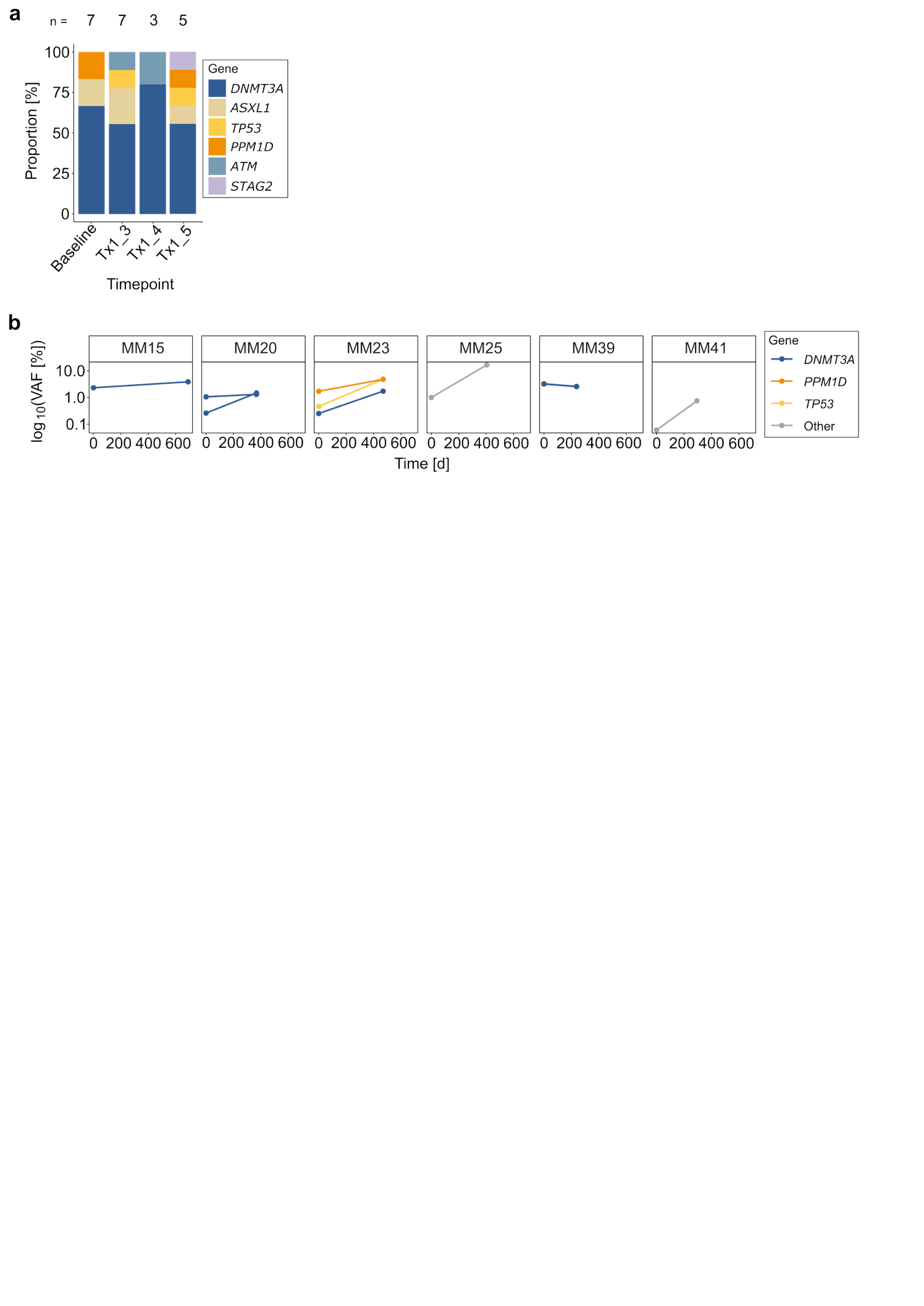
**

**Supplementary Figure S19:** Clonal dynamics of somatic clonal hematopoiesis-associated mutations post-autologous stem cell transplantation for MM19 and MM25. Variant allele frequencies (VAFs) of somatic mutations detected are shown for each patient. Lines represent individual mutations and are color-coded by the corresponding mutated gene.


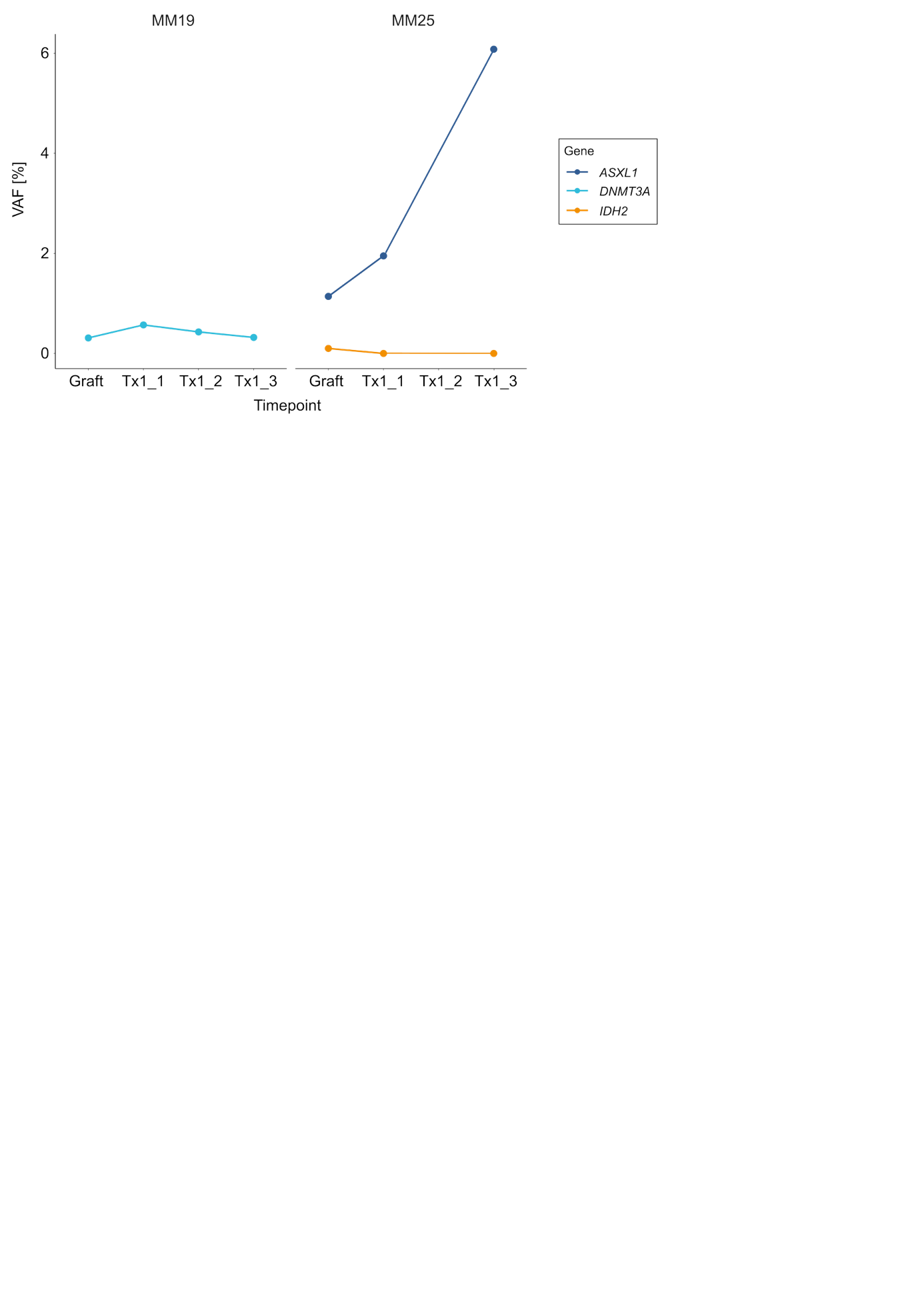


**Supplementary Figure S20:** Chromatin peak annotation in mitochondrial single-cell ATAC sequencing samples. Note that due to the bridge transfer cell type annotation all samples exhibit a uniform chromatin profile. **a)** Chromatin peak coverage across the human genome divided by chromosome. **b)** Upset plot displaying the annotation overlap and intersection size of genomic regions and features. **c)** Stacked bar plot illustrating the proportion of peaks assigned to different genomic features. bp=Base pairs. UTR=Untranslated region.

**
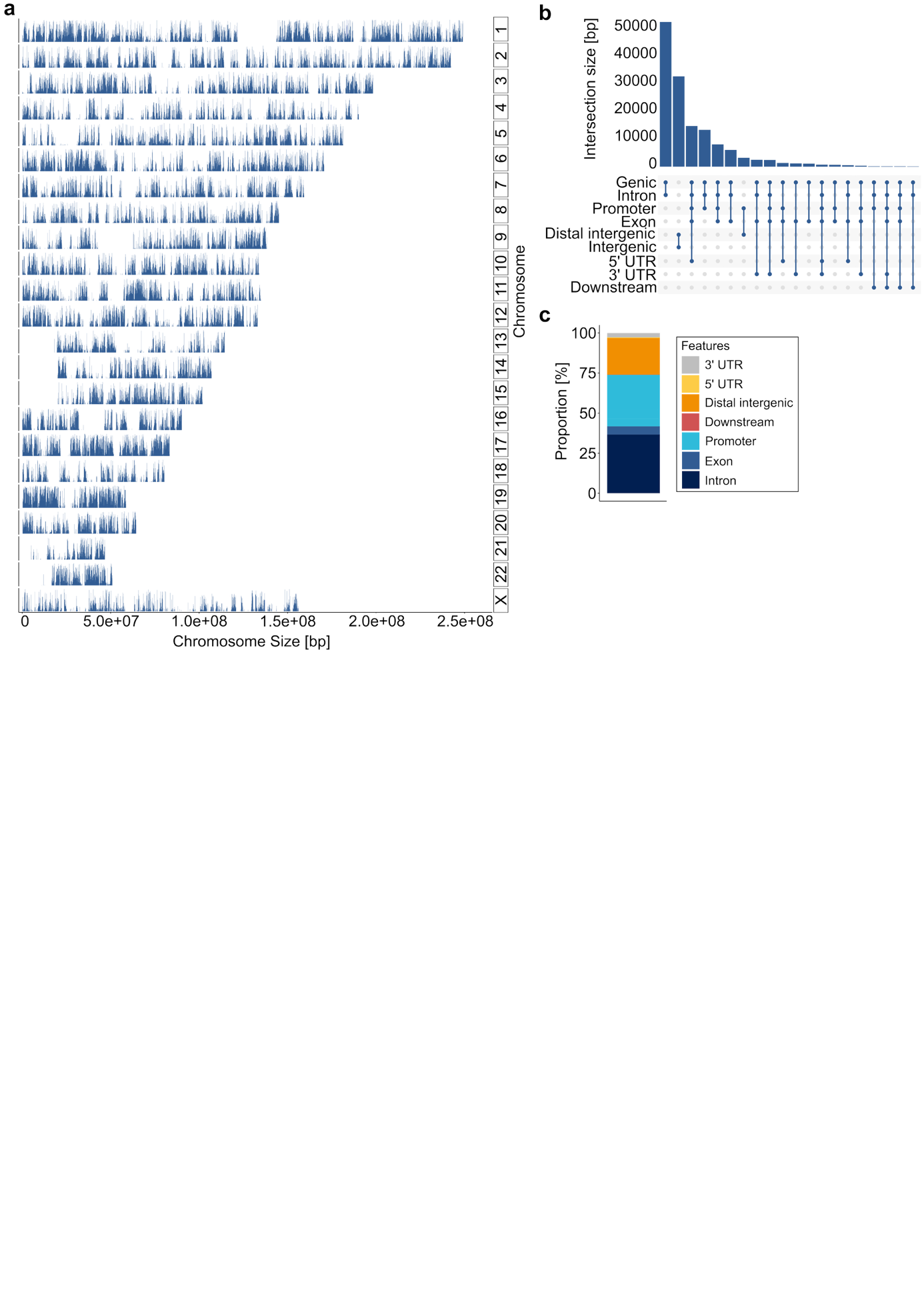
**

**Supplementary Figure S21:** Integrative analysis of chromatin accessibility landscapes in peripheral blood mononuclear cells (PBMCs) of graft and Tx1_3 samples from three multiple myeloma (MM) patients undergoing autologous stem cell transplantation. **a)** Reduced dimensionality projections according to the bridge PBMC projection reference embedding, split by sample and colored by cell types. Cell numbers are indicated above each panel. **b)** Dot plot showing mean gene activity of key marker genes across identified cell types. Dot size reflects the fraction of cells expressing each marker gene. B=B-cell. CD4 T=CD4^+^ T-cell. CD8 T=CD8^+^ T-cell. CD14 Mono=CD14^+^ monocyte. CD16 Mono=CD16^+^ monocyte. DC=Dendritic cell. HSPC=Hematopoietic stem and progenitor cell. NK=Natural killer cell. Other T=Other T-cell. Uniform manifold approximation and projection.


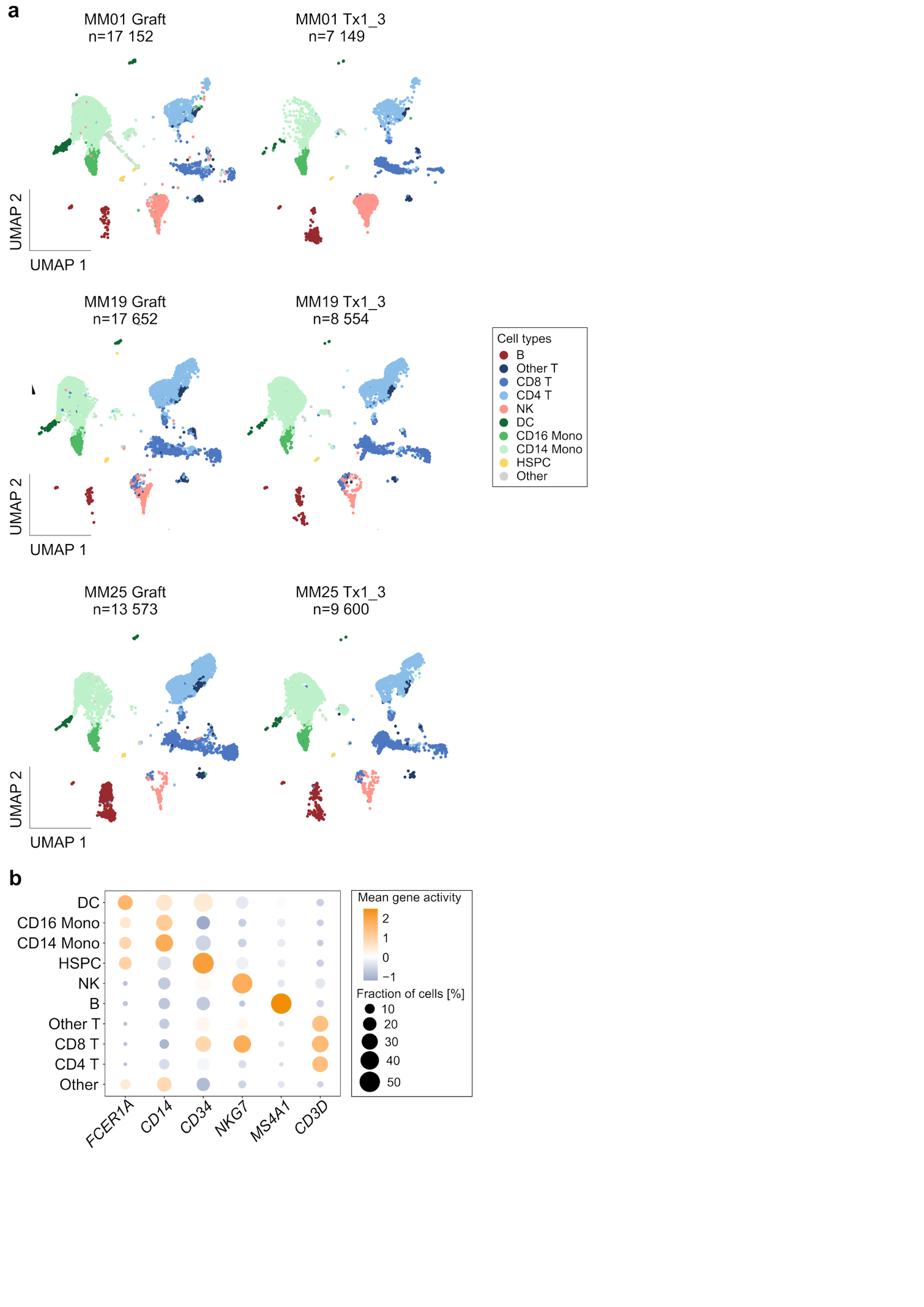


**Supplementary Figure S22:** Heatmap showing Z-scores of gene activity of differentially expressed genes by cell type to emphasize relative gene activity variation across patients and timepoints (graft and Tx1_3). Each row represents a gene, and each column corresponds to a sample. B=B-cell. CD4 T=CD4^+^ T-cell. CD8 T=CD8^+^ T-cell. CD14 Mono=CD14^+^ monocyte. CD16 Mono=CD16^+^ monocyte. DC=Dendritic cell. HSPC=Hematopoietic stem and progenitor cell. NK=Natural killer cell. Other T=Other T-cell.

**
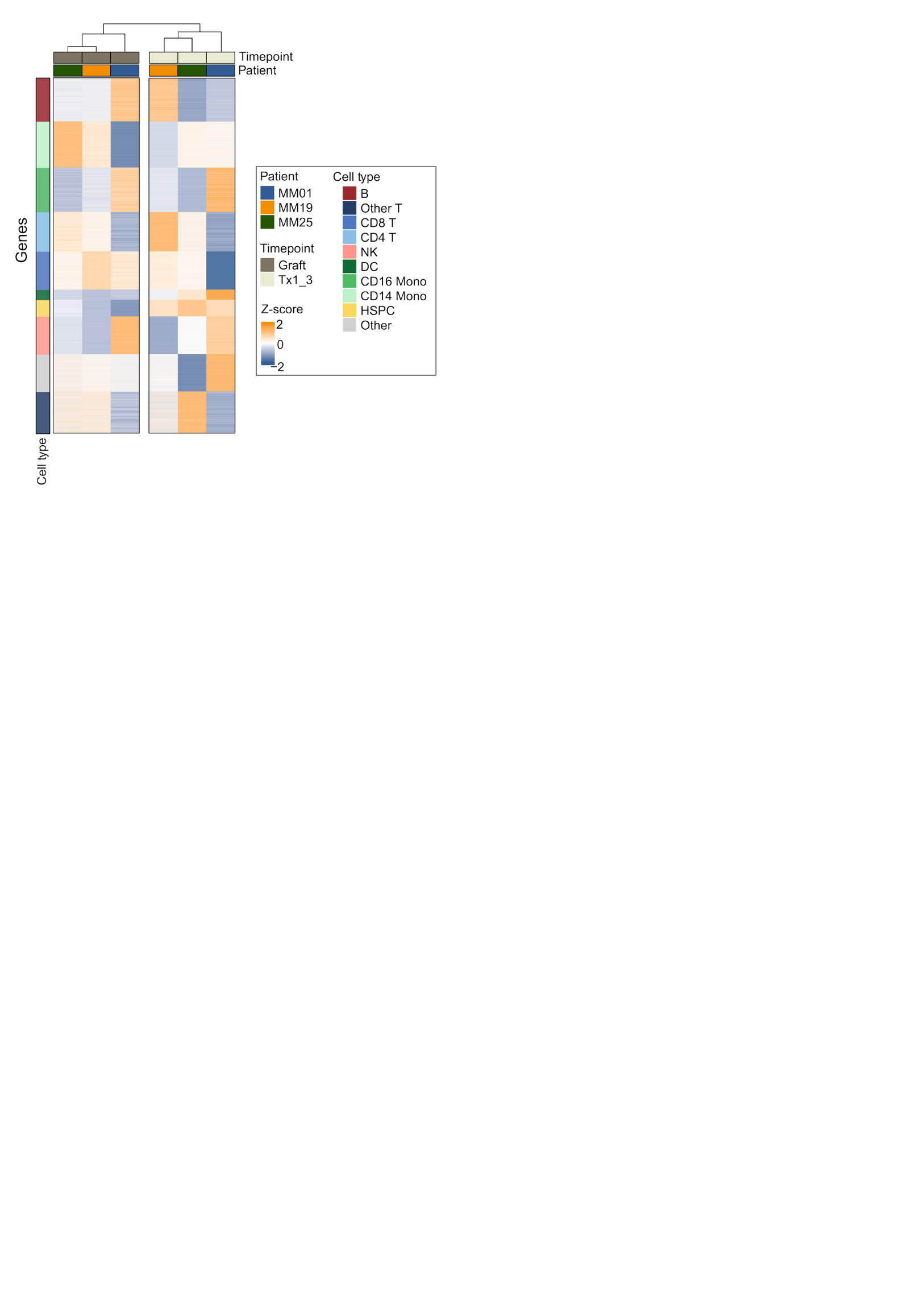
**

**Supplementary Figure S23:** Single sample gene set enrichment analysis showing normalized enrichment scores (NES) for significant pathways (p<0.01) in patient MM01. B=B-cell. CD4 T=CD4^+^ T-cell. CD8 T=CD8^+^ T-cell. CD14 Mono=CD14^+^ monocyte. CD16 Mono=CD16^+^ monocyte. DC=Dendritic cell. HSPC=Hematopoietic stem and progenitor cell. NK=Natural killer cell. Other T=Other T-cell.

**
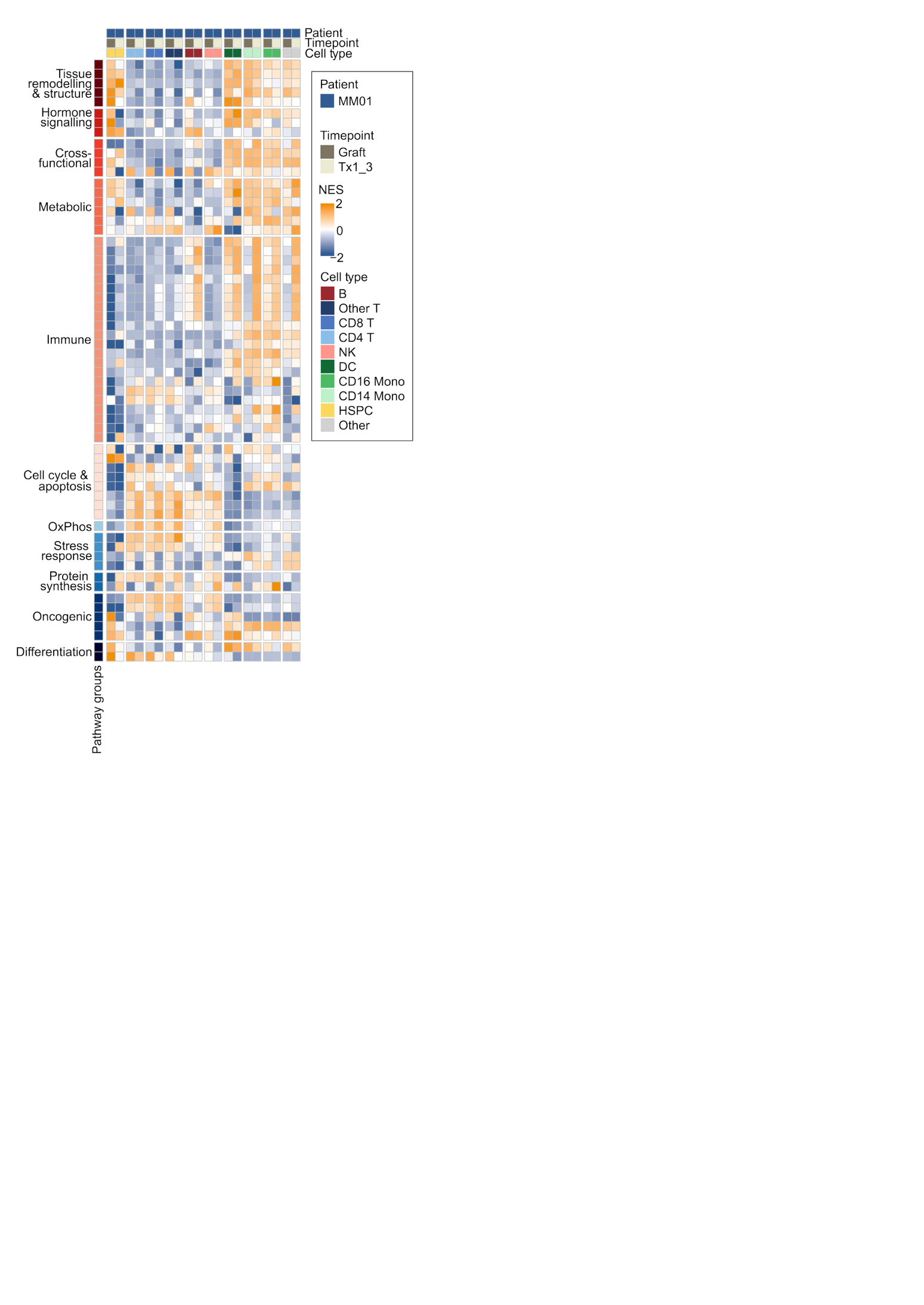
**

**Supplementary Figure S24:** Mean mitochondrial genome coverage across the mitochondrial DNA (mtDNA) chromosome/cell across all samples.


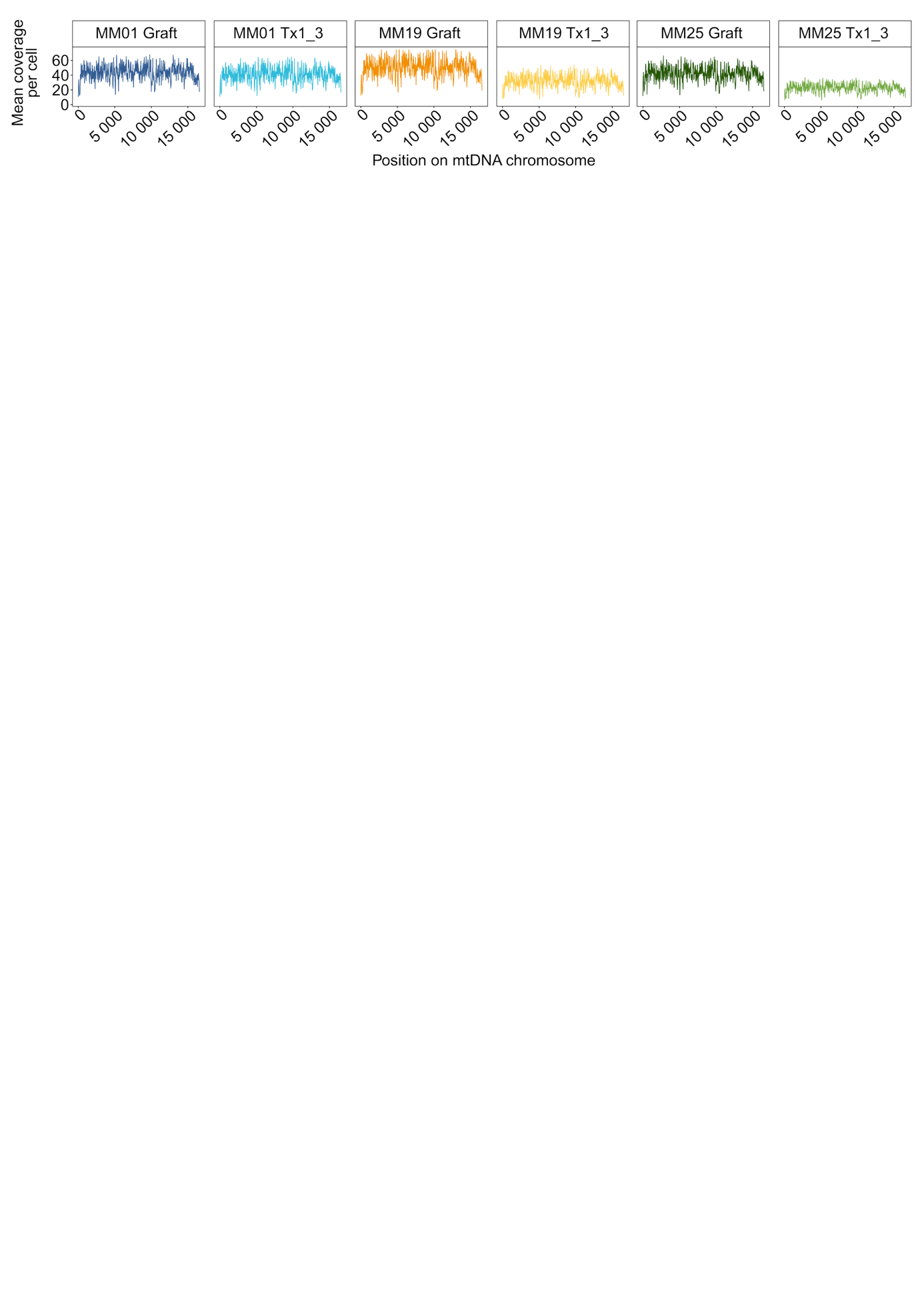


**Supplementary Figure S25:** Scatter plots identifying high-confidence mitochondrial variants with high strand concordance and high variance-mean ratio (VMR) from mitochondrial single-cell ATAC sequencing. Clonal heteroplasmic mutations are highlighted in red.

**
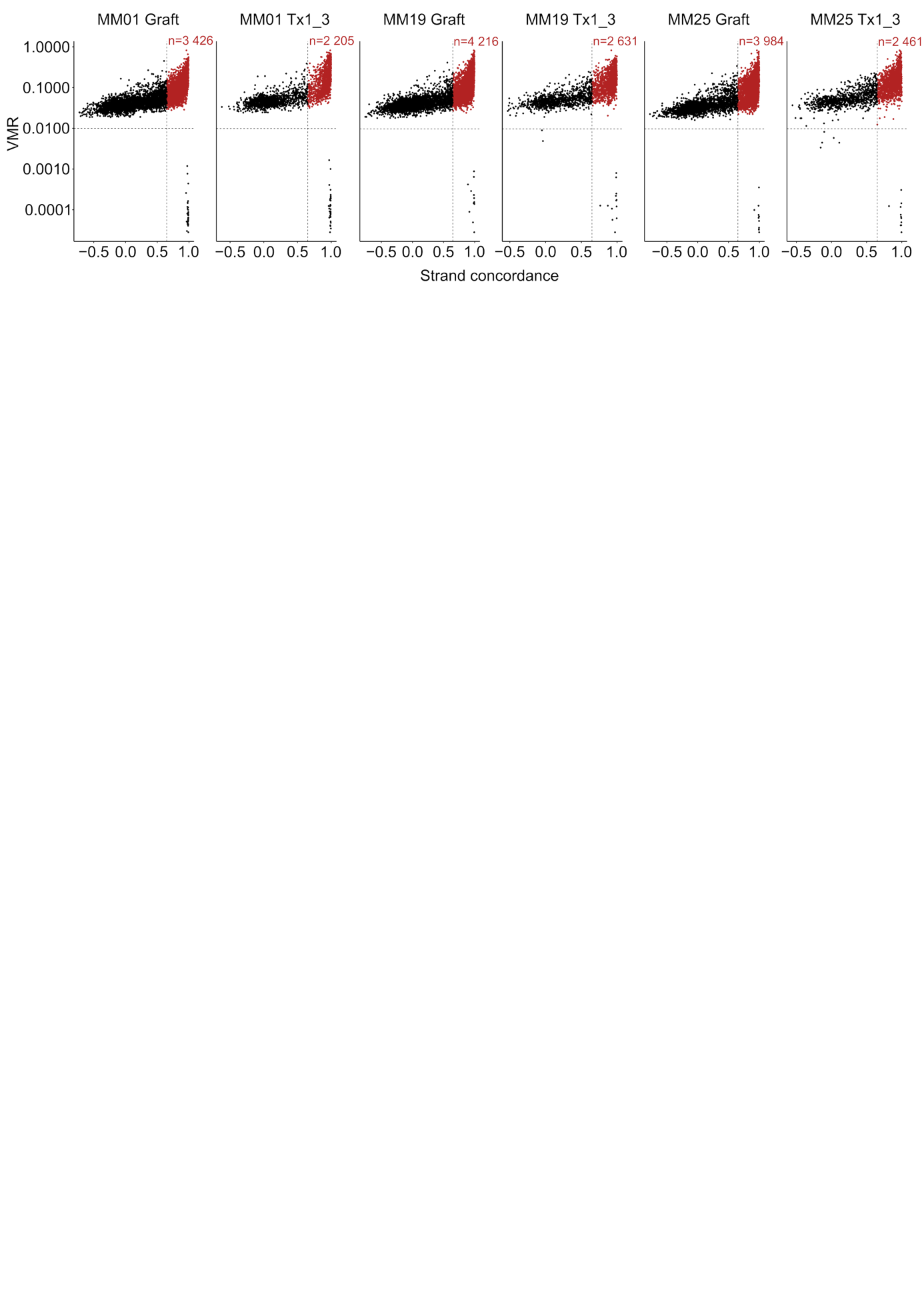
**

**Supplementary Figure S26:** Substitution profiles of all called mitochondrial mutations in the 96 trinucleotide contexts with mitochondrial single-cell ATAC sequencing. The y-axis indicates the observed-over-expected substitution rate for each class trinucleotide change, resolved by the heavy (H, orange) and light (L, blue) strands of the mitochondrial genome.

**
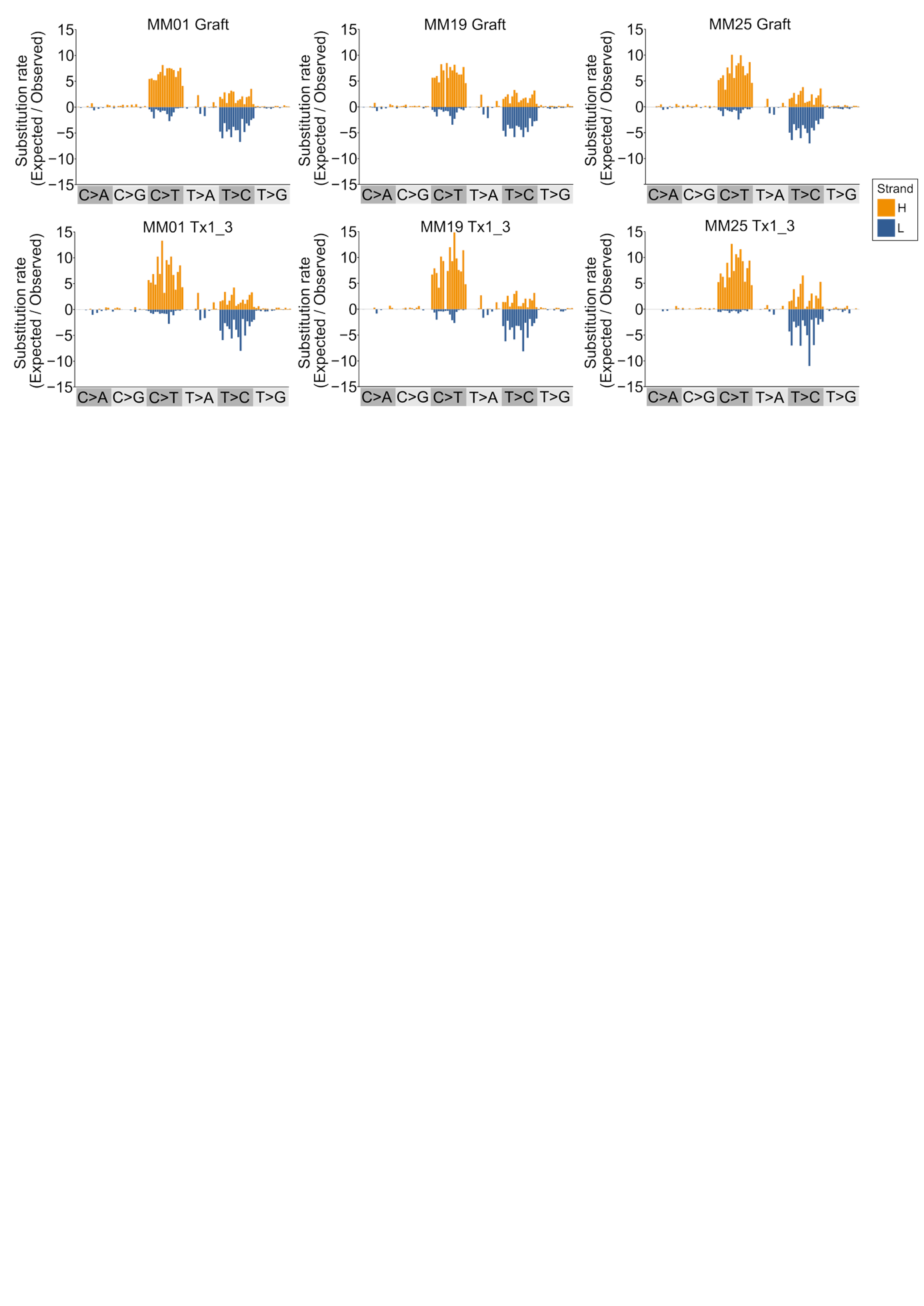
**

**Supplementary Figure S27:** Scatter plots showing the relationship between the number of cells carrying a variant and the mean heteroplasmy in positive cells across all samples.

**
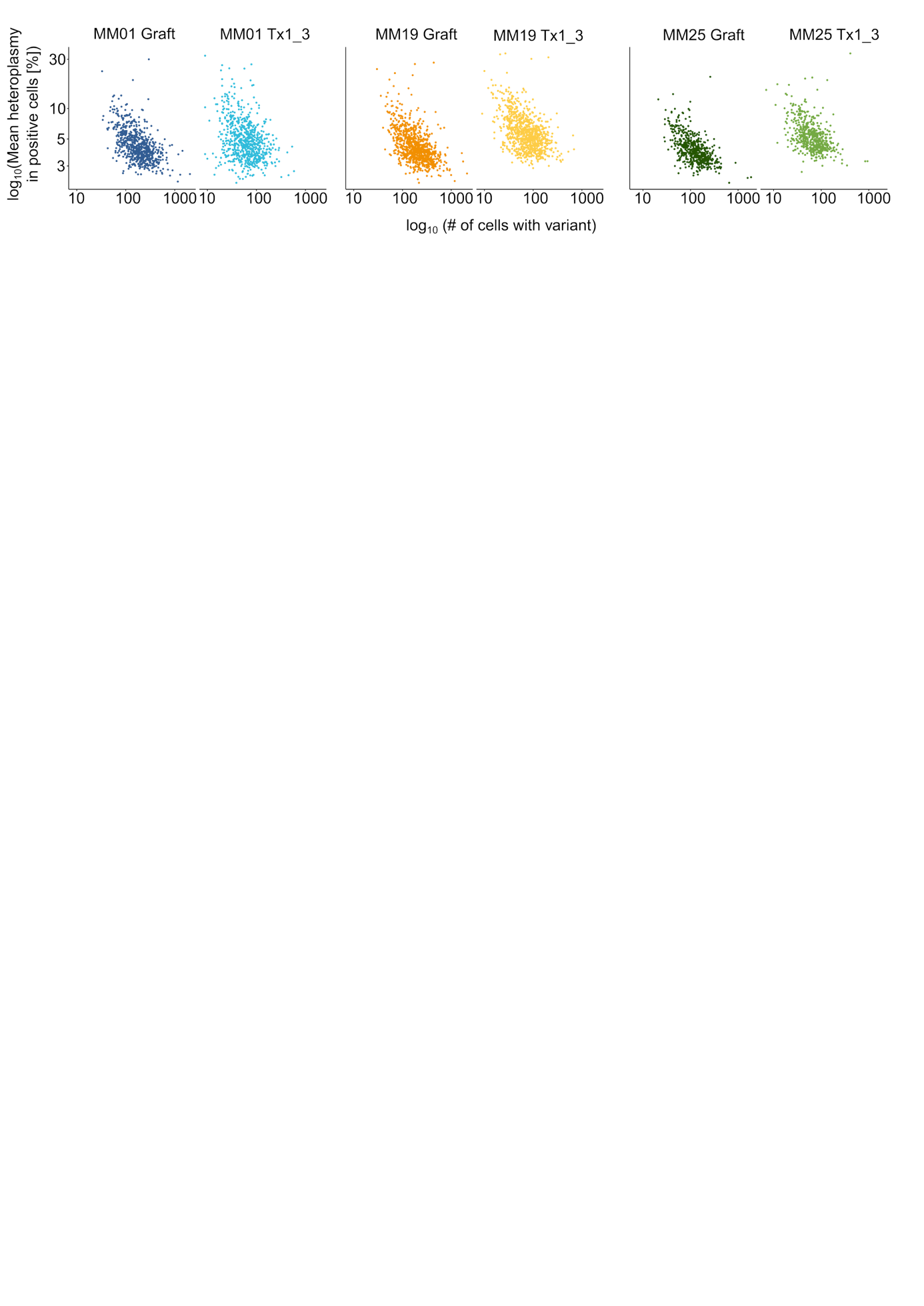
**

**Supplementary Figure S28:** Dot plot depicting mean gene activity of key T-cell markers across cells harboring mitochondrial DNA variants 5503T>C, 733T>C, and 15246G>A in MM25. The size of each dot represents the proportion of cells expressing the corresponding gene.

**
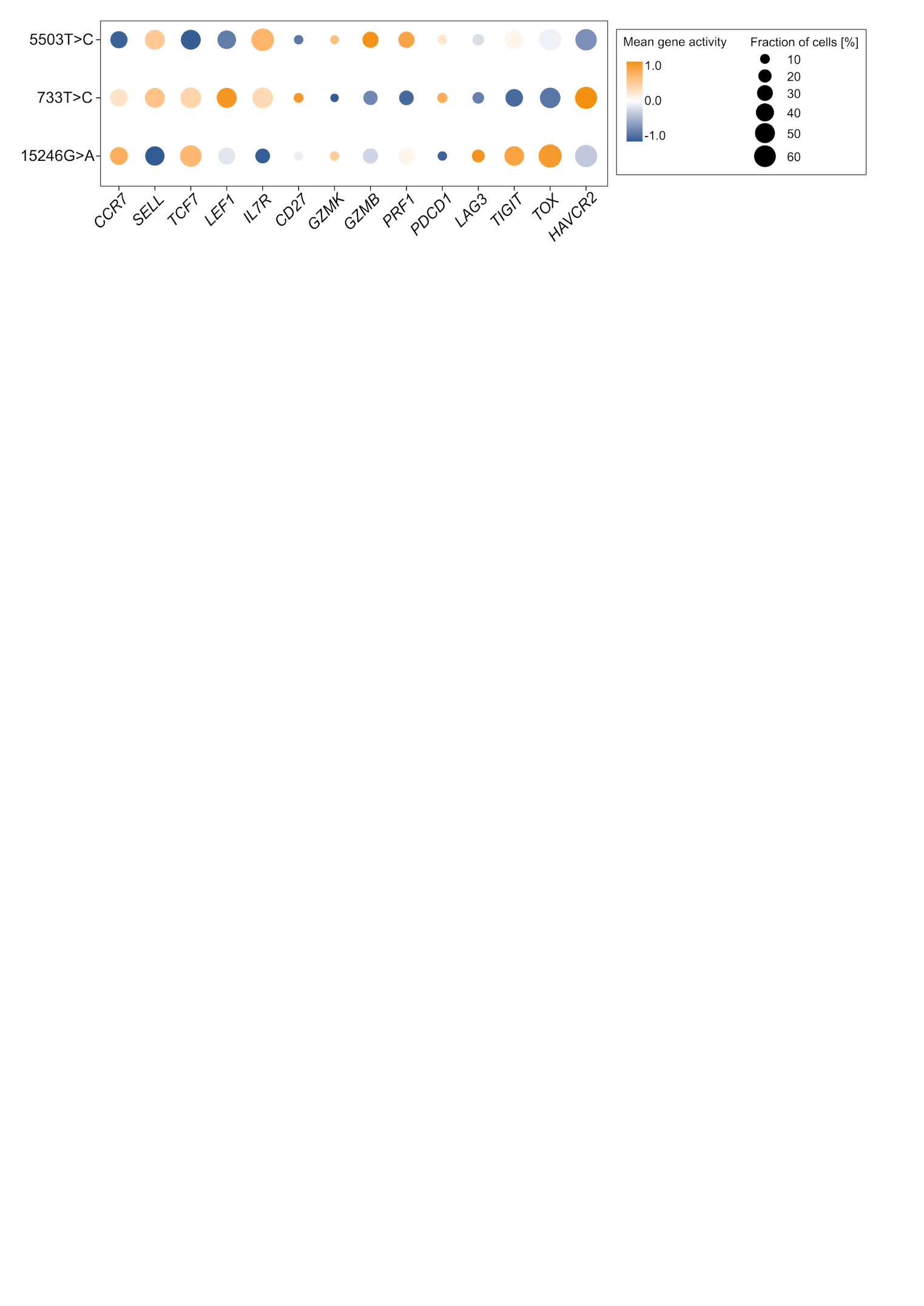
**

**Supplementary Figure S29:** Heteroplasmic mitochondrial mutation dynamics following autologous stem cell transplantation. **a)** Heatmap with hierarchical clustering showing the 40 most frequent heteroplasmic mitochondrial mutations for cells with a mean heteroplasmy >5% in MM01 Tx1_3. Only post-transplant cells harboring mitochondrial mutations originating from MM01 graft hematopoietic stem and progenitor cells (HSPCs) are shown. Mutation frequency across the entire sample is displayed as a bar plot on the right. **b)** Heatmap with hierarchical clustering showing the 40 most frequent heteroplasmic mitochondrial mutations for cells with a mean heteroplasmy per cell >5% in MM19 Tx1_3. Only post-transplant cells harboring mitochondrial mutations originating from MM19 graft HSPCs are shown. Mutation frequency across the entire sample is displayed as a bar plot on the right. B=B-cell. CD4 T=CD4^+^ T-cell. CD8 T=CD8^+^ T-cell. CD14 Mono=CD14^+^ monocyte. CD16 Mono=CD16^+^ monocyte. DC=Dendritic cell. NK=Natural killer cell. Other T=Other T-cell.


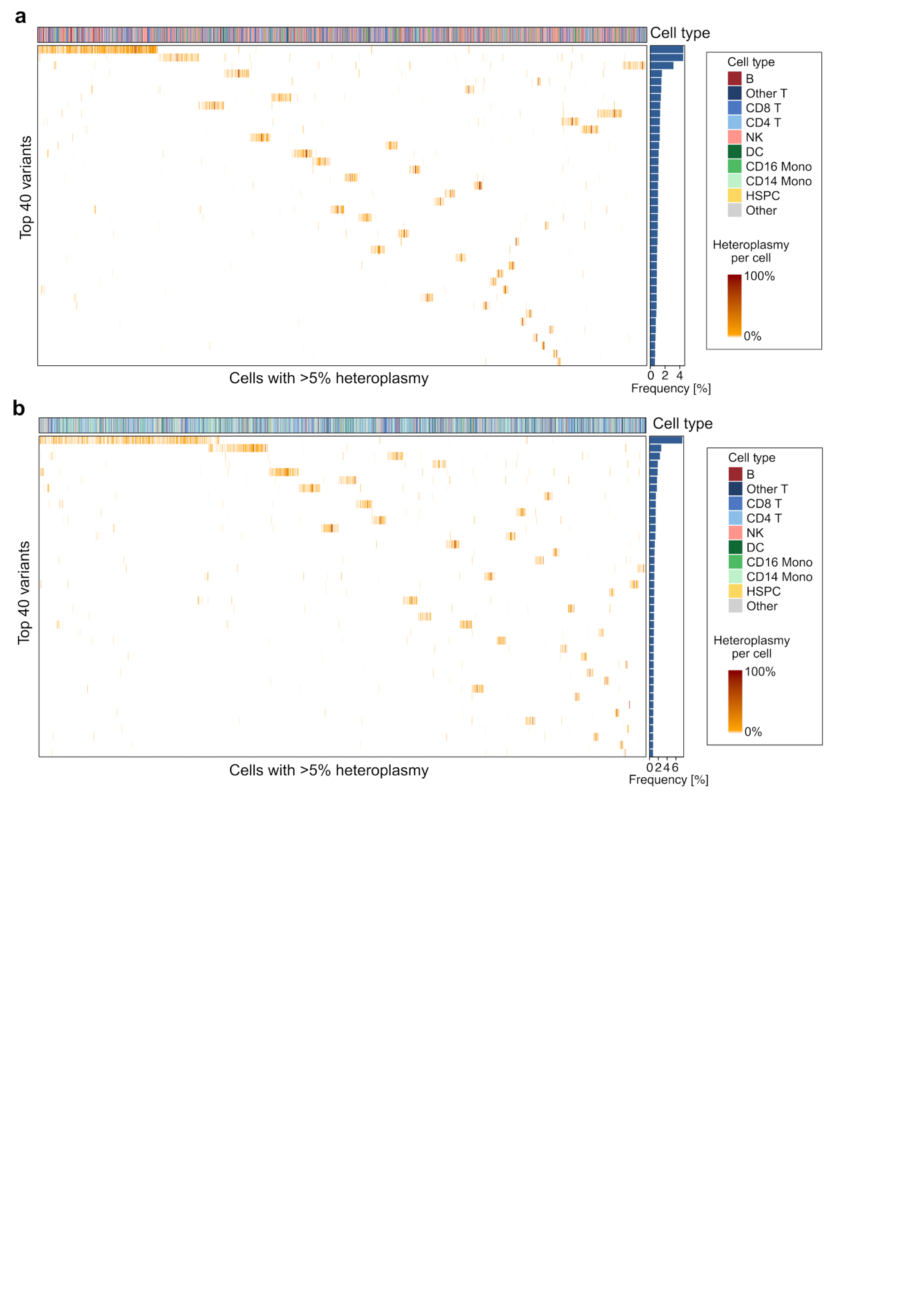


**Supplementary Figure S30:** Stacked bar plot showing the proportion of cell types per patient that harbor mitochondrial heteroplasmic mutations gained at Tx1_3 in addition to those already present from graft-derived hematopoietic stem and progenitor cells (HSPCs). B=B-cell. CD4 T=CD4^+^ T-cell. CD8 T=CD8^+^ T-cell. CD14 Mono=CD14^+^ monocyte. CD16 Mono=CD16^+^ monocyte. DC=Dendritic cell. NK=Natural killer cell. Other T=Other T-cell.


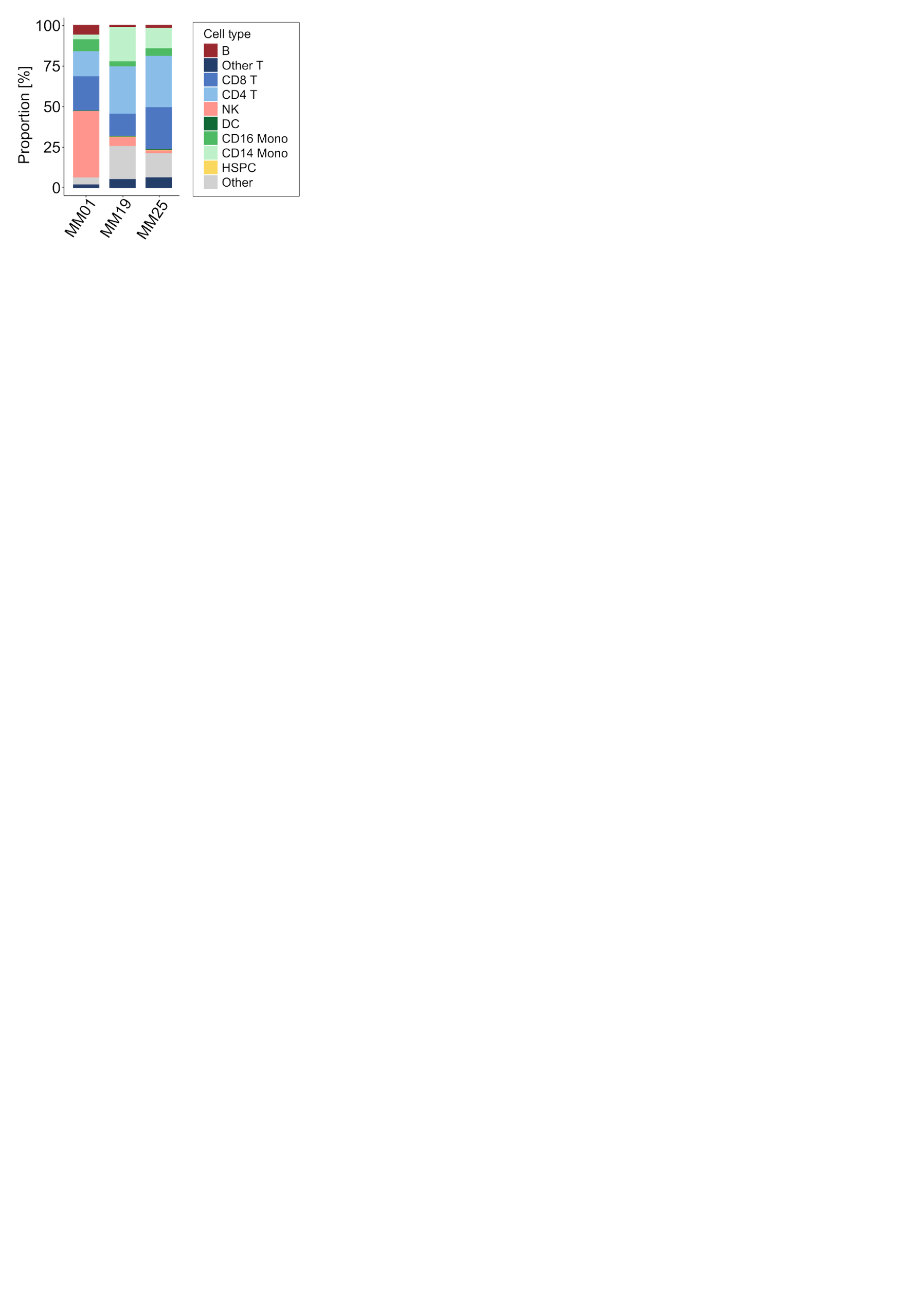


# References

1 Frick M, Chan W, Maximilian Arends C, Hablesreiter R, Halik A, Heuser M *et al.* Role of donor clonal hematopoiesis in allogeneic hematopoietic stem-cell transplantation. *J Clin Oncol* 2018; 37: 375–385.

2 Locher BN, Löwe P, Christen F, Damm F. Detection and characterization of clonal hematopoiesis. *Methods Mol Biol* 2025; 2865: 449–474.

3 Arends CM, Dimitriou S, Stahler A, Hablesreiter R, Strzelecka PM, Stein CM *et al.* Clonal hematopoiesis is associated with improved survival in patients with metastatic colorectal cancer from the FIRE-3 trial. *Blood* 2022; 139: 1593–1597.

4 Mölder F, Jablonski KP, Letcher B, Hall MB, Tomkins-Tinch CH, Sochat V *et al.* Sustainable data analysis with Snakemake. *F1000Res* 2021; 10: 33.

5 Arends CM, Liman TG, Strzelecka PM, Kufner A, Löwe P, Huo S *et al.* Associations of clonal hematopoiesis with recurrent vascular events and death in patients with incident ischemic stroke. *Blood* 2023; 141: 787–799.

6 Arends CM, Kopp K, Hablesreiter R, Estrada N, Christen F, Moll UM *et al.* Dynamics of clonal hematopoiesis under DNA-damaging treatment in patients with ovarian cancer. *Leukemia* 2024; 38: 1378–1389.

7 Panagiota V, Kerschbaum JF, Penack O, Stein CM, Arends CM, Koenecke C *et al.* Clinical implications and dynamics of clonal hematopoiesis in anti-CD19 CAR T-cell treated patients. *Hemasphere* 2023; 7: E957.

8 bcl2fastq, https://support.illumina.com/sequencing/sequencing_software/bcl2fastq-conversion-software.html. ; v2.20.0.422.

9 Fgbio Tools, https://github.com/fulcrumgenomics/fgbio. .

10 Miller C, Walker J. Modified GRCh38 genome with U2AF1 region fix (v1.2020_04_01). *Zenodo* 2021.

11 Li H, Durbin R. Fast and accurate long-read alignment with Burrows-Wheeler transform. *Bioinformatics* 2010; 26: 589–595.

12 Broad Institute, Picard Toolkit, GitHub repository. http://broadinstitutegithubio/picard/. 2019.

13 Schneider VA, Graves-Lindsay T, Howe K, Bouk N, Chen HC, Kitts PA *et al.* Evaluation of GRCh38 and de novo haploid genome assemblies demonstrates the enduring quality of the reference assembly. *Genome Res* 2017; 27: 849–864.

14 Depristo MA, Banks E, Poplin R, Garimella K V., Maguire JR, Hartl C *et al.* A framework for variation discovery and genotyping using next-generation DNA sequencing data. *Nat Genet* 2011; 43: 491–501.

15 Van der Auwera GA, O’Connor BD. Genomics in the cloud: using Docker, GATK, and WDL in Terra (1st Edition). *O’Reilly Media* 2020.

16 Lai Z, Markovets A, Ahdesmaki M, Chapman B, Hofmann O, Mcewen R *et al.* VarDict: A novel and versatile variant caller for next-generation sequencing in cancer research. *Nucleic Acids Res* 2016; 44.

17 Wang K, Li M, Hakonarson H. ANNOVAR: Functional annotation of genetic variants from high-throughput sequencing data. *Nucleic Acids Res* 2010; 38.

18 Pruitt KD, Tatusova T, Maglott DR. NCBI reference sequences (RefSeq): A curated non-redundant sequence database of genomes, transcripts and proteins. *Nucleic Acids Res* 2007; 35.

19 Landrum MJ, Lee JM, Benson M, Brown GR, Chao C, Chitipiralla S *et al.* ClinVar: Improving access to variant interpretations and supporting evidence. *Nucleic Acids Res* 2018; 46: D1062–D1067.

20 Liu X, Jian X, Boerwinkle E. dbNSFP: A lightweight database of human nonsynonymous SNPs and their functional predictions. *Hum Mutat* 2011; 32: 894–899.

21 Chen S, Francioli LC, Goodrich JK, Collins RL, Kanai M, Wang Q *et al.* A genomic mutational constraint map using variation in 76,156 human genomes. *Nature* 2024; 625: 92–100.

22 Sherry ST, Ward M, Sirotkin K. dbSNP-Database for single nucleotide polymorphisms and other classes of minor genetic variation. *Genome Res* 1999; 8: 677–679.

23 Tate JG, Bamford S, Jubb HC, Sondka Z, Beare DM, Bindal N *et al.* COSMIC: The catalogue of somatic mutations in cancer. *Nucleic Acids Res* 2019; 47: D941–D947.

24 Ioannidis NM, Rothstein JH, Pejaver V, Middha S, McDonnell SK, Baheti S *et al.* REVEL: An ensemble method for predicting the pathogenicity of rare missense variants. *Am J Hum Genet* 2016; 99: 877–885.

25 Monks A, Scudiero D, Skehan P, Shoemaker R, Paull K, Vistica D *et al.* Feasibility of a high-flux anticancer drug screen using a diverse panel of cultured human tumor cell lines. *J Natl Cancer Inst* 1991; 83: 757–766.

26 Landrum MJ, Lee JM, Riley GR, Jang W, Rubinstein WS, Church DM *et al.* ClinVar: Public archive of relationships among sequence variation and human phenotype. *Nucleic Acids Res* 2014; 42.

27 Feusier JE, Arunachalam S, Tashi T, Baker MJ, VanSant-Webb C, Ferdig A *et al.* Large-scale identification of clonal hematopoiesis and mutations recurrent in blood cancers. *Blood Cancer Discov* 2021; 2: 226–237.

28 Yannakou CK, Jones K, McBean M, Thompson ER, Ryland GL, Doig K *et al.* ASXL1 c.1934dup;p.Gly646Trpfs∗12-a true somatic alteration requiring a new approach. *Blood Cancer J* 2017; 7.

29 Robinson JT, Thorvaldsdóttir H, Winckler W, Guttman M, Lander ES, Getz G *et al.* Integrative genomics viewer. *Nat Biotechnol* 2011; 29: 24–26.

30 Wickham H, Averick M, Bryan J, Chang W, McGowan L, François R *et al.* Welcome to the Tidyverse. *J Open Source Softw* 2019; 4: 1686.

31 Wickham H, Bryan J. readxl: Read Excel Files. R package version1.4.5, https://github.com/tidyverse/readxl, https://readxl.tidyverse.org. 2025.

32 Ooms J. writexl: Export data frames to Excel ‘xlsx’ format. R package version 1.5.1. 2024.

33 Wickham H., François R., Henry L., Müller K., Vaughan D. dplyr: A grammar of data manipulation. R package version 1.1.4. 2023.

34 Lawrence M, Huber W, Pagès H, Aboyoun P, Carlson M, Gentleman R *et al.* Software for computing and annotating genomic ranges. *PLoS Comput Biol* 2013; 9.

35 Wickham H. stringr: Simple, consistent wrappers for common string operations. R package version 1.5.1. 2023.https://github.com/tidyverse/stringr/issues.

36 Fang H, Wu Y, Narzisi G, O’rawe JA, Jimenez Barrón LT, Rosenbaum J *et al.* Reducing INDEL calling errors in whole genome and exome sequencing data. *Genome Med* 2014; : 89.

37 Robertson NA, Latorre-Crespo E, Terradas-Terradas M, Lemos-Portela J, Purcell AC, Livesey BJ *et al.* Longitudinal dynamics of clonal hematopoiesis identifies gene-specific fitness effects. *Nat Med* 2022; 28: 1439–1446.

38 Lareau CA, Liu V, Muus C, Praktiknjo SD, Nitsch L, Kautz P *et al.* Mitochondrial single-cell ATAC-seq for high-throughput multi-omic detection of mitochondrial genotypes and chromatin accessibility. *Nat Protoc* 2023; 18: 1416–1440.

39 Satpathy AT, Granja JM, Yost KE, Qi Y, Meschi F, McDermott GP *et al.* Massively parallel single-cell chromatin landscapes of human immune cell development and intratumoral T cell exhaustion. *Nat Biotechnol* 2019; 37: 925–936.

40 Lareau CA, Ludwig LS, Muus C, Gohil SH, Zhao T, Chiang Z *et al.* Massively parallel single-cell mitochondrial DNA genotyping and chromatin profiling. *Nat Biotechnol* 2021; 39: 451–461.

41 Thibodeau A, Eroglu A, McGinnis CS, Lawlor N, Nehar-Belaid D, Kursawe R *et al.* AMULET: a novel read count-based method for effective multiplet detection from single nucleus ATAC-seq data. *Genome Biol* 2021; 22.

42 Stuart T, Srivastava A, Madad S, Lareau CA, Satija R. Single-cell chromatin state analysis with Signac. *Nat Methods* 2021; 18: 1333–1341.

43 Satija R, Farrell JA, Gennert D, Schier AF, Regev A. Spatial reconstruction of single-cell gene expression data. *Nat Biotechnol* 2015; 33: 495–502.

44 Yu G, Wang LG, He QY. ChIP seeker: An R/Bioconductor package for ChIP peak annotation, comparison and visualization. *Bioinformatics* 2015; 31: 2382–2383.

45 Hao Y, Stuart T, Kowalski MH, Choudhary S, Hoffman P, Hartman A *et al.* Dictionary learning for integrative, multimodal and scalable single-cell analysis. *Nat Biotechnol* 2024; 42: 293–304.

46 Hao Y, Hao S, Andersen-Nissen E, Mauck WM, Zheng S, Butler A *et al.* Integrated analysis of multimodal single-cell data. *Cell* 2021; 184: 3573-3587.e29.

47 Love MI, Huber W, Anders S. Moderated estimation of fold change and dispersion for RNA-seq data with DESeq2. *Genome Biol* 2014; 15.

48 Korotkevich G, Sukhov V, Budin N, Shpak B, Artyomov MN, Sergushichev A. Fast gene set enrichment analysis. *bioRxiv* 2021; : 060012.

49 Mootha VK, Lindgren CM, Eriksson K-F, Subramanian A, Sihag S, Lehar J *et al.* PGC-1α-responsive genes involved in oxidative phosphorylation are coordinately downregulated in human diabetes. *NATURE GENETICS VOLUME* 2003; 34: 267–273.

50 Subramanian A, Tamayo P, Mootha VK, Mukherjee S, Ebert BL, Gillette MA *et al.* Gene set enrichment analysis: A knowledge-based approach for interpreting genome-wide expression profiles. *Proc Natl Acad Sci USA* 2005; 43: 15545–15550.

51 Bengtsson H. A unifying framework for parallel and distributed processing in R using Futures. *R J* 2021; 13: 208–227.

52 Wickham H., Henry L. purrr: Functional programming tools. R package version 1.0.4, https://github.com/tidyverse/purrr, https://purrr.tidyverse.org/. 2025.

53 Hoffman P. SeuratDisk: Interfaces for HDF5-based single cell file formats. https://mojaveazure.github.io/seurat-disk/, https://github.com/mojaveazure/seurat-disk. 2023.

54 Rainer J. EnsDb.Hsapiens.v86: Ensembl based annotation package_. R package version 2.99.0. 2017.

55 Wickham H. ggplot2: Elegant graphics for data analysis. *Springer-Verlag New York* 2016.

56 van den Brand T. ggh4x: Hacks for ’ggplot2’_. R package version 0.3.0,  <https://CRAN.R-project.org/package=ggh4x. 2024.

57 Wild F. lsa: Latent semantic analysis_. R package version 0.73.3,  <https://CRAN.R-project.org/package=lsa>. 2022.

58 Zeileis A, Grothendieck G. zoo: S3 Infrastructure for regular and irregular time series. *J Stat Softw* 2005; 6: 1–27.

59 Morgan M, Obenchain V, Hester J, Pagès H. SummarizedExperiment:  SummarizedExperiment container_. R package version 1.28.0, <https://bioconductor.org/packages/SummarizedExperiment>. 2022.

60 Barrett T., Dowle M., Srinivasan A., Gorecki J., Chirico M., Hocking T. *et al.* data.table: Extension of `data.frame`_. R package version 1.17.0,  <https://CRAN.R-project.org/package=data.table>. 2025.

61 Pedersen TL. patchwork: The Composer of Plots_. R package version 1.3.0,  <https://CRAN.R-project.org/package=patchwork>. 2024.

62 Brunson JC., Read QD. ggalluvial: Alluvial Plots in ‘ggplot2’.” R package version 0.12.5, http://corybrunson.github.io/ggalluvial/. 2023.

63 Wickham H. Reshaping data with the reshape package. *J Stat Softw* 2007; 12.http://www.jstatsoft.org/.

64 Slowikowski K. ggrepel: Automatically position non-overlapping text labels with ’ggplot2’_. R package version 0.9.6, <https://CRAN.R-project.org/package=ggrepel>. 2024.

65 Conway JR, Lex A, Gehlenborg N. UpSetR: An R package for the visualization of intersecting sets and their properties. *Bioinformatics* 2017; 33: 2938–2940.

66 Gu Z, Gu L, Eils R, Schlesner M, Brors B. Circlize implements and enhances circular visualization in R. *Bioinformatics* 2014; 30: 2811–2812.

67 Müller K., Wickham H. tibble: Simple data frames_. R package version 3.2.1,  <https://CRAN.R-project.org/package=tibble>. 2023.

68 Wickham H., Vaughan D., Girlich M. tidyr: Tidy messy data_. R package version  1.3.1, <https://CRAN.R-project.org/package=tidyr>. 2024.

69 Gu Z, Eils R, Schlesner M. Complex heatmaps reveal patterns and correlations in multidimensional genomic data. *Bioinformatics* 2016; 32: 2847–2849.

70 Bengtsson H. matrixStats: Functions that apply to rows and columns of matrices  (and to vectors)_. R package version 1.5.0,  <https://CRAN.R-project.org/package=matrixStats>. 2025.
